# Differential gene expression in small and large rainbow trout derived from two seasonal spawning groups

**DOI:** 10.1186/1471-2164-15-57

**Published:** 2014-01-22

**Authors:** Andrea L Kocmarek, Moira M Ferguson, Roy G Danzmann

**Affiliations:** 1 Department of Integrative Biology, University of Guelph, 50 Stone Rd. East, Guelph, Ontario N1G 2W1, Canada

**Keywords:** Growth, Microarray, Rainbow trout, Liver, White muscle, Season

## Abstract

**Background:**

Growth in fishes is regulated via many environmental and physiological factors and is shaped by the genetic background of each individual. Previous microarray studies of salmonid growth have examined fish experiencing either muscle wastage or accelerated growth patterns following refeeding, or the influence of growth hormone and transgenesis. This study determines the gene expression profiles of genetically unmanipulated large and small fish from a domesticated salmonid strain reared on a typical feeding regime. Gene expression profiles of white muscle and liver from rainbow trout (*Oncorhynchus mykiss*) from two seasonal spawning groups (September and December lots) within a single strain were examined when the fish were 15 months of age to assess the influence of season (late fall vs. onset of spring) and body size (large vs. small).

**Results:**

Although *IGFBP1* gene expression was up-regulated in the livers of small fish in both seasonal lots, few expression differences were detected in the liver overall. Faster growing Dec. fish showed a greater number of differences in white muscle expression compared to Sept. fish. Significant differences in the GO Generic Level 3 categories ‘response to external stimulus’, ‘establishment of localization’, and ‘response to stress’ were detected in white muscle tissue between large and small fish. Larger fish showed up-regulation of cytoskeletal component genes while many genes related to myofibril components of muscle tissue were up-regulated in small fish. Most of the genes up-regulated in large fish within the ‘response to stress’ category are involved in immunity while in small fish most of these gene functions are related to apoptosis.

**Conclusions:**

A higher proportion of genes in white muscle compared to liver showed similar patterns of up- or down-regulation within the same size class across seasons supporting their utility as biomarkers for growth in rainbow trout. Differences between large and small Sept. fish in the ‘response to stress’ and ‘response to external stimulus’ categories for white muscle tissue, suggests that smaller fish have a greater inability to handle stress compared to the large fish. Sampling season had a significant impact on the expression of genes related to the growth process in rainbow trout.

## Background

Growth in fishes is a complex trait that impacts many components of fitness. Large body size provides an advantage when competing for mates and resources and can provide energy reserves during times of famine or reproduction [1,2]. The initiation of sexual maturation in certain species can also be coupled to variation in growth rate and is therefore, a size-dependent phenomenon. Unlike mammals, most species of fish continue to grow throughout their lives [3]. As skeletal muscle composes a large portion of body mass, fish growth is primarily dependent on an increase in muscle mass [4]. Skeletal muscle can be divided into two types; red muscle is used for slow continuous swimming while white muscle is used for rapid bursts of speed [5]. White muscle is utilized preferentially over red muscle as an energy reserve and exhibits wide changes in hypertrophic and hyperplastic states dependent on the physiological condition of the fish [6-8].

Fish growth is controlled by a complex array of extrinsic and intrinsic factors and their interactions. Extrinsic factors such as variation in circannual photoperiod [9], and changes in water temperature [10,11] have largely predictable effects on growth rates. Other factors are more complex and relate to interactions between extrinsic and intrinsic factors that regulate growth, such as individual responses to stress and disease [12], population density [13], social status [14], and feeding regime [15]. Variation in growth in response to these factors has a strong genetic basis as indicated by the detection of moderate to high heritability values [16-18] and chromosomal regions (quantitative trait loci, QTL) associated with variation in body size [19,20]. While it is likely that all chromosomes have genes that contribute to, or interact with growth regulation, some of these will have stronger effects than others. For example, 15 of the 29 linkage groups in rainbow trout (*Oncorhynchus mykiss*) have QTL for body weight and/or condition factor with genome-wide effects, with an additional 8 linkage groups housing QTL with chromosome-wide effects [20]. This indicates that perhaps three quarters of rainbow trout linkage groups contain genomic regions with major influences on growth. However, it is important to emphasize that our knowledge of growth-regulating regions is incomplete as genetically-based responses to environmental inputs are varied and can often produce conflicting results. For instance, microarray studies have examined fish experiencing muscle wastage [6-8] with the premise that genes down-regulated during muscle wastage will be up-regulated during muscle growth and *vice versa*. These studies have identified many candidate genes for growth including cytoskeletal, transcription/translation, protein turnover, and metabolic genes but with some conflicting results. For instance, myosin genes showed down-regulation during muscle wastage [7] and during GH-stimulated growth [21] in rainbow trout.

One of the main intrinsic regulators of growth is the somatotrophic axis, which is composed of growth hormone (GH), GH regulating factors (GH releasing factor and somatostatin), and the products GH releases from the liver by binding to GH receptors, including insulin and insulin-like growth factors (IGFs) [22]. Indeed, many of these genes (*GH, GHR2, GHR1*, *IGF2,* and *IGF1*) are up-regulated in a variety of tissues (e.g., skeletal muscle, brain, and liver) from faster growing strains or individuals within several species including Nile tilapia, *Oreochromis niloticus*[23], smooth tongued sole, *Cynoglossus semilaevis*[24], and channel catfish, *Ictalurus punctatus*[25]. Studies with GH-transgenic rainbow trout have also shown that liver-specific gene expression patterns are more similar to those observed in faster growing domesticated rainbow trout, compared to slower growing wild-type fish [26-28]. In fact, the *IGF2* gene was highly up-regulated in the faster growing GH transgenic/domestic fish compared to wild-type fish [27] suggesting that increased growth is in part regulated by changes in the GH axis. Genes with differential expression were assigned to 16 Gene Ontology (GO) categories, with those comprising response to stimulus, lipid metabolism, precursor metabolites and energy, and cell/tissue structure and development having the greatest number of differences. Thus, many of the differentially expressed genes may be interconnected in regulatory pathways stimulated by those in the somatotrophic axis.

Studies of liver and muscle gene expression in salmonids indicate that genes central to energy metabolism, carbohydrate and lipid metabolism, and cytoskeletal components are major GO categories associated with growth differences [7,27-29]. A number of muscle genes showing major expression differences between a fast (directionally selected) and a normally growing line of rainbow trout have been identified with RNAseq, and these have been verified by screening additional families with SNP variants identified within these genes [30]. Significant differences in the distribution of SNP alleles were reported for genes such as *glucose phosphate isomerase, enolase, ATP2A1*, and several structural genes including myosin binding proteins, *fast myotomal muscle actin 2, troponin C,* and *troponin T-2* between fast and slow growing rainbow trout. A large number of mitochondrial specific genes including several NADH dehydrogenase subunit genes, cytochrome b, and cytochrome c oxidase subunits, and different ATPase genes were also differentially expressed. Although it was only reported that muscle tissue was used, the finding that a large proportion of mitochondrial-specific genes were highly representative of differences between fast and slow growing trout is suggestive that a high proportion of red muscle tissue was analyzed in combination with white muscle tissue.

We utilized a 44 K feature Atlantic salmon (*Salmo salar*) microarray to examine differential mRNA levels in fast- and slow-growing rainbow trout from the same commercial strain but produced in two seasons (September and December). Previous studies show that Atlantic salmon microarrays are suitable for use in gene expression studies of other salmonid species [31-33]. Our goal was to identify genes that show similar patterns of differential expression between large and small rainbow trout in both liver and white muscle regardless of season for use as biomarkers of growth in selective breeding programs. The analysis of global gene expression in large and small fish reared under standard conditions also provides an understanding of typical growth patterns and complements previous analyses where growth in rainbow trout was altered by adjusting feeding regimes [6], domestication, or by the addition of GH [26,27]. If differences in gene expression explain a large portion of body size variation in fishes, then many of the genes identified in studies of accelerated growth or muscle wastage are also expected to show differential expression in normal growing large and small fish. Based upon previous studies we predicted that: (1) genes related to the metabolism of carbohydrates and lipids, energy production, and members of the somatotrophic axis, including GH receptors, insulin, and IGFs should be down-regulated in the liver and white muscle of small fish compared to large fish; (2) genes for cytoskeleton components including, actin, myosin, and troponin are also expected to be differentially expressed in both tissues. Specifically, genes involved in cytoskeletal structuring will be down-regulated while myostatin will be up-regulated in small fish compared to larger fish, and (3) genes for liver-specific, lipid binding, cytoplasmic components, signaling, and transcription will be up-regulated in the livers of small fish compared to large fish.

## Results

### Growth and gender

**Figure 1:**
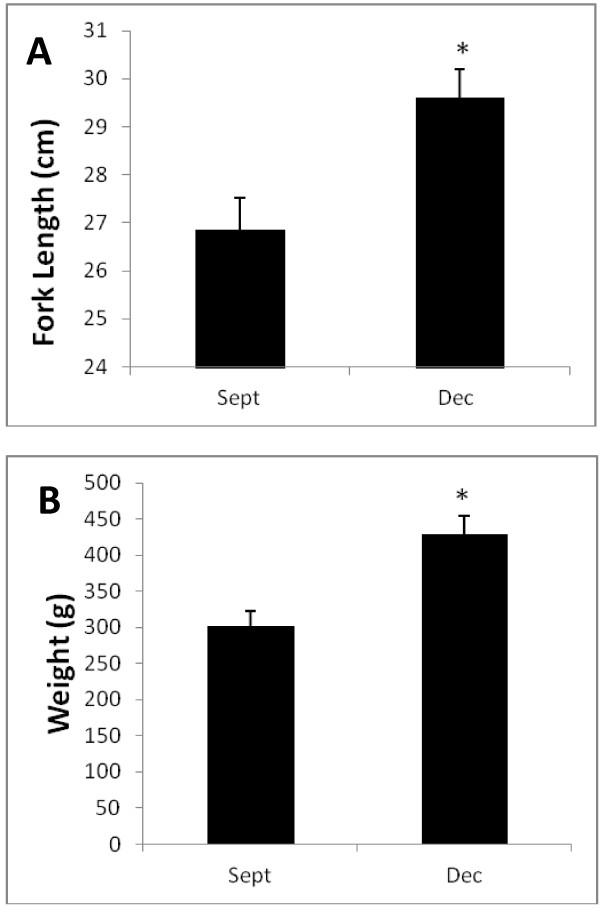
**Fork length and body weight by half-sibling lots.** Fork length **(A)** and body weight **(B)** differences between the half-sibling lots. Bars with * are significantly different from the Sept. fish, as assessed by a t-test (n =55 and 50, respectively; p ≤ 0.05).

**Figure 2:**
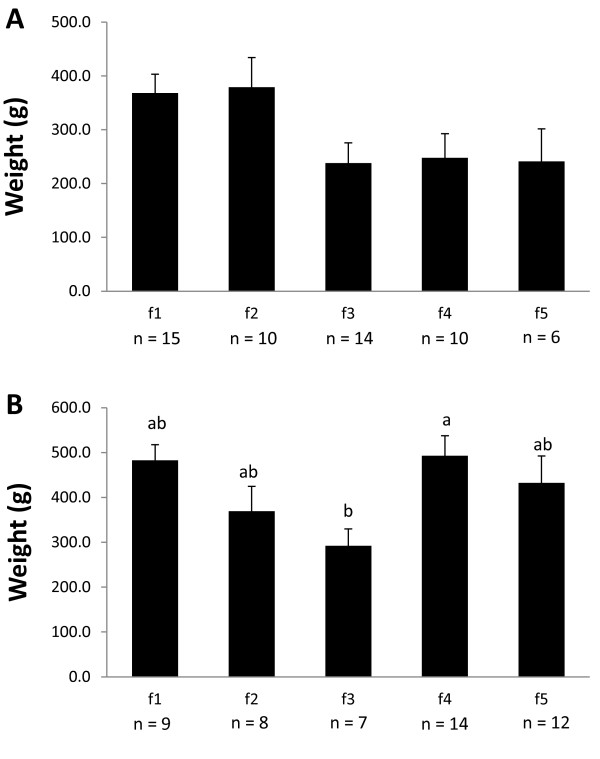
**Body weight by maternal parent.** Body weight within the **A)** Sept. and **B)** Dec. families. Bars that do not share a common letter are significantly different, as assessed by one-way ANOVA and Tukey’s test (p ≤ 0.05).

**Figure 3:**
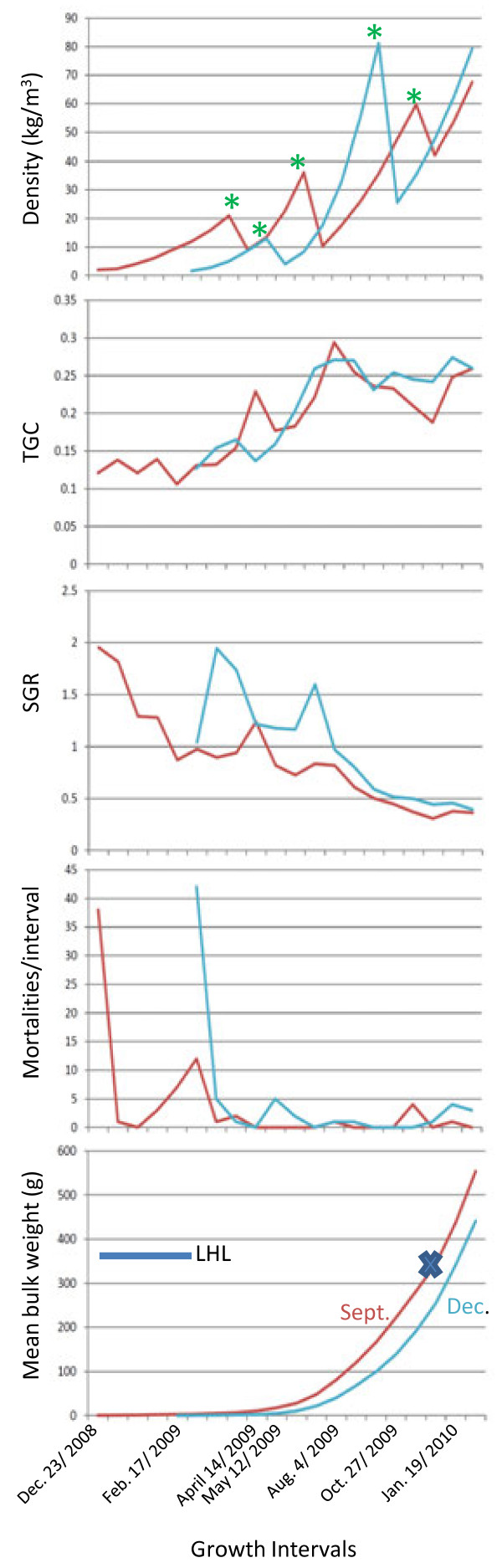
**Growth profiles of the September, 2008 (Sept.) and December, 2008 (Dec.) lots of rainbow trout reared at the Alma Aquaculture Research Station.** Density, thermal growth coefficient (TGC), specific growth rate (SGR), mortalities per measurement interval, and bulk weight are shown for the Sept. and Dec. lots and are all aligned on the same temporal scale. LHL indicates the Life History Lag of the Dec. fish compared to Sept. fish (i.e. 89 days). Growth profiles of the Dec. fish would need to shift to the ‘left’ by this distance on all the graphs to place the growth profiles of the Dec. fish on a comparable chronological scale to those of the Sept. fish. Asterisks above the density plots on the top figure indicate intervals when fish were either moved to larger tanks or were thinned to minimize density effects. Microarray sampling of the Sept. lot occurred in the interval shown with an X on the Sept. line, but growth recorded from siblings within this lot are shown for completeness. The Dec. lot was sampled on that last day shown in the graph.

The Dec. fish were significantly heavier and longer than the Sept. fish (Figure 1). When the full-sib families within each of the Sept. and Dec. half-sib families were examined for parental effects on weight, significant dam effects were found in the Dec. fish (Figure 2). Thermal growth coefficients increased during the spring and summer and plateaued or decreased during the fall and winter in both the Sept. and Dec. lots (Figure 3). Thus, TGC can be taken as an estimate of growth efficiencies in the fish during a given growth interval.

Genotyping with the sex-specific marker indicated that the microarray analysis was based on 9 males (5 = large, 4 = small) and 11 females (5 = large, 6 = small) in the Sept. lot. In the Dec. lot there were 10 males (6 = large, 4 = small) and 10 females (4 = large, 6 = small). Thus, there was no gender bias in the pools of large and small fish analyzed in either lot.

### Liver

#### Effects of body size

From the 44 K gene microarray comparing the large fish to the small fish in each lot, 575 and 151 sequences were differentially expressed (p ≤ 0.05, fold change ≥ 1.2) in the livers of the Sept. and Dec. lots, respectively (Additional file 1: Table S1, Additional file 2: Table S2, Additional file 3: Table S3 and Additional file 4: Table S4). In both lots, the majority of the genes showed up-regulation in the small fish; with 425 up-regulated in the Sept. lot and 146 up-regulated in the Dec. lot. A large portion of the sequences, 267 in the Sept. lot and 49 in the Dec. lot, were not identified by either Agilent or Blast2GO and are listed as unknown probe IDs. Throughout the remaining text, genes showing ≥2.4-fold up-regulation are referred to as **highly** or **major** up-regulated genes and represent highly significant differences in gene expression between large and small fish. Within this category, *insulin-like growth factor binding protein 1* (*IGFBP1*) showed major up-regulation in the small Dec. fish compared to the large fish, as well as significant up-regulation in the small Sept. fish (Table 1). In addition, significant up-regulation was also detected in small fish across both lots for the following genes: *protein transport sec61, glutamine synthetase*; *zinc finger protein 568,* and *novel protein human titin*. Similarly, the only gene showing major up-regulation in large Dec. fish compared to small Dec. fish expressed within both lots was *polycystic kidney disease protein 1-like 3 precursor*. However, the gene expression differences were localized to different probe IDs and therefore may represent duplicated or orthologous copies of the gene. Interestingly, the same form of *lipocalin precursor* (A_05_P414837) exhibited major up-regulation in both the small Sept. fish and the large Dec. fish. Seven probe ID sequences were associated with higher expression in both large and small fish across seasons (Additional file 1: Table S1 and Additional file 2: Table S2).

**Table 1:** Identified genes up-regulated ≥2.4-fold in the liver

**Gene name**	**Gene number**	Fold change*	**p-value†**
***Large Sept. fish***			
Complement C1q-like protein 4 precursor	A_05_P332842	2.994	7.70E-03
Trophoblast glycol	A_05_P341612	2.915	1.67E-03
***Small Sept. fish***			
Glutathione s-transferase p	A_05_P278137	4.709	7.55E-05
Terf1 -interacting nuclear factor 2	A_05_P292537	3.673	3.30E-02
Thioredoxin	A_05_P465152	3.190	1.59E-02
Hemagglutinin/amebocyte aggregation factor precursor	A_05_P250874	3.127	2.51E-02
Beta-galactosyltransferase 1	A_05_P430162	3.034	1.19E-03
Proteasome activator complex subunit 3	A_05_P254864	2.843	4.13E-02
Lipocalin precursor	A_05_P414837	2.673	1.90E-03
***Large Dec. fish***			
Nattectin precursor	A_05_P490962	6.024	4.75E-02
Lipocalin precursor	A_05_P414837	3.817	4.60E-02
Polycystic kidney disease protein 1-like 3 precursor	A_05_P488952	2.732	4.89E-02
Zinc transporter ZIP3	A_05_P372187	2.688	4.66E-02
***Small Dec. fish***			
Insulin-like growth factor binding protein 1	A_05_P489857	3.274	2.88E-04
Delta(14)-sterol reductase	A_05_P304042	2.787	2.66E-02
e3 ubiquitin-protein ligase neurl3-like	A_05_P444687	2.490	3.55E-03

*Fold change is the average difference in expression as measured by the microarray.

†Measures the significance of the difference in expression between the small and large fish.

The biological process level 2 GO category with the largest number of up-regulated sequences in both the large and small Sept. fish and the small Dec. fish was ‘cellular process’ (GO:0009987) (Additional file 5: Table S5). A list of the Agilent probe IDs that correspond to the individual GO Level 2 and Level 3 categories is provided in Additional file 6: Table S6 and Additional file 7: Table S7. Cellular process was one of three GO categories to contain identified up-regulated sequences in the large Dec. fish at biological process level 2. At biological process level 3, the ‘cellular metabolic process’ category (GO:0044237), a subcategory of the cellular process category, contained the largest number of up-regulated sequences in the both the large and small Sept. fish and the small Dec. fish (Additional file 8: Table S8). However, no significant differences in the observed number of genes assigned to either the level 2 or level 3 Blast2GO categories were detected between the large and small fish within either lot (Additional file 5: Table S5 and Additional file 8: Table S8).

#### Effects of season (lot)

**Figure 4:**
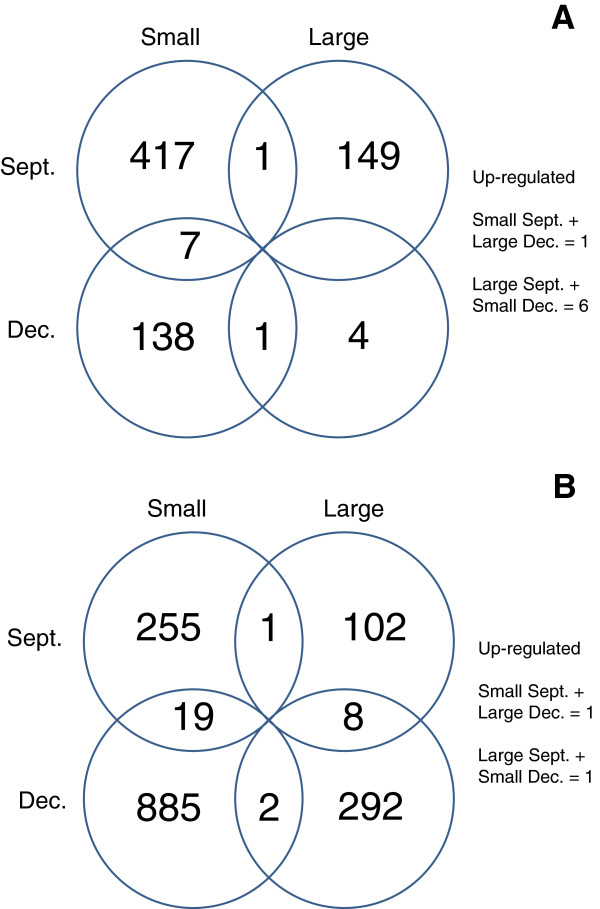
**Venn diagrams showing the number of genes up-regulated in large and small fish.** Genes up-regulated in large and small fish and common to each size class across seasons, as well as shared between size classes within a season for liver **(A)** and white muscle **(B)** tissue. Not shown is the fact that 6 genes were shared between large Sept. and small Dec. fish, and 1 gene was shared between small Sept. and large Dec. fish in liver tissue. In white muscle, 1 gene was shared between large Sept. and small Dec. fish, and 1 gene was shared between small Sept. and large Dec. fish.

None of the probe ID targets demonstrated significant up-regulation in large fish across both the Sept. and Dec. lots (Additional file 1: Table S1 and Additional file 3: Table S3), with the exception of *polycystic kidney disease protein 1-like 3 precursor* (mentioned above). However, 7 probe IDs were consistently associated with significant up-regulation across both lots within the small fish (Figure 4) (Additional file 2: Table S2 and Additional file 4: Table S4). Similarly, no significant differences in the distribution of genes assigned to various level 2 and 3 GO categories were found between the large fish in the Sept. and Dec. lots and the small fish in the Sept. and Dec. lots (Additional file 5: Table S5 and Additional file 8: Table S8).

### Muscle

#### Effects of body size

In muscle, 386 sequences were differently expressed between large and small fish in the Sept. lot and 1208 sequences were differently expressed in the Dec. lot (Additional file 9: Table S9, Additional file 10: Table S10, Additional file 11: Table S11 and Additional file 12: Table S12). The majority of genes showed up-regulation in the small fish compared to the large fish with 275 up-regulated in the small Sept. fish and 906 up-regulated in the small Dec. fish (Additional file 10: Table S10). Eighty-five and 543 sequences were unidentified in the Sept. and Dec. lots, respectively (Additional file 11: Table S11 and Additional file 12: Table S12). Representatives of the highly up-regulated genes in large fish compared to small fish in both the Dec. and Sept. lots are: *fibrinogen gamma chain* and *polypeptide* genes, *HIG1 domain family member 2a, max protein, DNA-damage-inducible transcript 4-like protein, ADP/ATP tanslocase 2,* C*1 inhibitor*, and *mgc821112 protein* (Table 2). Similarly, highly up-regulated genes in small fish compared to large fish within at least one lot are: *parvalbumin 2; parvalbumin beta-2; glyceraldehyde-3-phosphate dehydrogenase; thrombospondin 2,* and *myosin heavy chain.* In total, 8 probe ID targets demonstrated significant up-regulation in large fish in both the Sept. and Dec. lots (Additional file 9: Table S9 and Additional file 11: Table S11), and 19 probe IDs were linked to significant up-regulation in small fish across both seasonal lots (Additional file 10: Table S10 and Additional file 12: Table S12). Two unnamed probe IDs gave inconsistent size associations (i.e., associated with higher expression in both large and small fish across lots) (Additional file 11: Table S11 and Additional file 12: Table S12). Certain gene classes also exhibited significantly higher up-regulation in both large and small fish within or between seasonal lots. These genes are likely representative of different ortholgous and/or paralogous copies (i.e. they possess different probe ID designations in the large and small fish) of the same gene class and include: *ADP/ATP translocase, complement C1q-like protein 4 precursor, alpha actin*, and *middle subunit protein*.

**Table 2:** Named genes up-regulated in ≥2.4-fold the white muscle

**Gene name**	**Gene number**	Fold change*	**p-value†**
***Large Sept. fish***			
Max protein	A_05_P478412	5.618	4.14E-03
DNA-damage-inducible transcript 4-like protein	A_05_P249334	5.128	9.71E-04
ADP/ATP translocase 2	A_05_P276614	4.000	8.42E-03
Complement C1q-like protein 4 precursor	A_05_P332842	3.891	9.94E-03
HIG1 domain family member 2a	A_05_P433247	2.801	3.92E-02
Polycystic kidney disease protein 1-like 3 precursor	A_05_P299292	2.717	7.16E-03
C-type lectin	A_05_P249684	2.646	1.13E-02
Fibrinogen gamma chain	A_05_P480537	2.625	3.06E-02
Fibrinogen gamma chain	A_05_P364872	2.584	3.24E-02
40s ribosomal protein s30	A_05_P377337	2.558	7.26E-03
Cathepsin m precursor	A_05_P251889	2.525	8.39E-03
Fibrinogen alpha chain	A_05_P332052	2.506	1.24E-02
Beta-enolase-like isoform 1	A_05_P482637	2.500	4.06E-02
Retinol-binding protein 2	A_05_P254734	2.481	2.03E-02
Fibrinogen gamma chain	A_05_P464657	2.463	1.82E-02
Trichohyalin	A_05_P439982	2.433	2.17E-02
Complement C1q-like protein 2 precursor	A_05_P252174	2.404	1.23E-02
***Small Sept. fish***			
Parvalbumin 2	A_05_P423182	6.722	8.14E-04
Calcium-binding and coiled-coil domain-containing protein 1 isoform 2	A_05_P276634	5.305	3.25E-05
Parvalbumin beta-2	A_05_P429727	3.595	1.67E-03
Glyceraldehyde-3-phosphate dehydrogenase	A_05_P419277	3.542	1.31E-04
Thrombospondin 2	A_05_P322687	3.014	2.58E-02
Calcium binding and coiled-coil domain like	A_05_P276654	2.971	8.67E-04
Myosin heavy chain	A_05_P393052	2.682	1.37E-02
Alpha actin	A_05_P449267	2.550	9.65E-04
***Large Dec. fish***			
Fibrinogen gamma polypeptide	A_05_P450362	25.126	1.71E-02
C1 inhibitor	A_05_P475617	4.717	3.85E-02
HIG1 domain family member 2a	A_05_P368647	4.049	3.85E-02
mgc82112 protein	A_05_P250149	3.831	2.60E-02
Transcription factor jun-b	A_05_P266894	3.759	3.03E-02
Butyrate response factor 1	A_05_P376337	3.559	1.17E-02
Fucolectin-4 precursor	A_05_P463327	3.436	4.43E-02
Iron zinc purple acid phosphatase-like	A_05_P411342	3.344	4.52E-02
Transketolase	A_05_P414317	3.333	4.38E-02
Lysosome membrane protein 2-like	A_05_P413782	3.247	3.45E-02
Lactate dehydrogenase b	A_05_P444697	3.115	4.38E-02
Eukaryotic translation elongation factor 1 alpha 1	A_05_P362822	2.976	3.11E-02
Acidic mammalian chitinase-like	A_05_P391302	2.941	3.58E-02
Growth arrest and dna-damage- beta	A_05_P409552	2.841	1.08E-02
Lipoprotein lipase	A_05_P429962	2.778	1.58E-02
Neutral alpha-glucosidase ab-like	A_05_P259469	2.688	3.22E-02
Max protein	A_05_P478412	2.681	2.36E-02
Metallothionein	A_05_P249474	2.674	2.70E-02
Delta-6 fatty acyl desaturase	A_05_P248839	2.653	4.06E-02
Flavin reductase	A_05_P418547	2.632	2.75E-02
Eukaryotic translation elongation factor 1 alpha 1	A_05_P449262	2.618	3.06E-02
Lctacalcin	A_05_P488907	2.611	1.49E-02
Liver-expressed antimicrobial peptide 2	A_05_P475047	2.571	3.33E-02
BolA-like protein 2	A_05_P336112	2.564	1.38E-02
Annexin a1	A_05_P287152	2.506	1.91E-02
Cathepsin d	A_05_P251434	2.500	1.02E-02
Purine nucleoside phosphorylase	A_05_P415112	2.494	2.04E-02
Eukaryotic translation elongation factor 1 alpha 1	A_05_P454907	2.488	3.53E-02
Heat shock protein 90	A_05_P249734	2.457	3.06E-02
Litaf-like protein	A_05_P486262	2.457	4.47E-02
Alpha actin	A_05_P423987	2.451	1.28E-02
Xylose isomerise	A_05_P277217	2.439	1.64E-02
Middle subunit	A_05_P434332	2.427	3.47E-02
Uncharacterized protein C8orf4 homolog	A_05_P262634	2.410	2.26E-02
***Small Dec. fish***			
rRNA promoter binding protein	A_05_P425972	5.474	7.07E-03
Myosin light chain 3-like	A_05_P248844	4.690	1.29E-03
Myosin heavy chain	A_05_P277167	4.167	3.87E-03
rRNA promoter binding protein	A_05_P393707	3.572	9.75E-04
Tropomyosin 2	A_05_P488277	3.453	2.01E-02
Troponin T, cardiac muscle	A_05_P255254	3.386	1.04E-02
Troponin slow skeletal muscle	A_05_P277927	3.332	7.86E-03
Tropomyosin 3	A_05_P377692	3.075	1.33E-02
Parvalbumin 2	A_05_P423182	3.032	3.13E-02
Parvalbumin beta-2	A_05_P429727	2.982	1.82E-02
Eukaryotic translation elongation factor 1 epsilon 1	A_05_P468757	2.899	4.73E-03
Tropomyosin partial	A_05_P336882	2.894	7.12E-03
Senescence-associated protein	A_05_P262709	2.819	1.64E-03
Myosin binding protein cardiac	A_05_P277492	2.688	3.53E-03
Keratin-associated protein 10-4	A_05_P485782	2.441	3.35E-03

*Fold change is the average difference in expression as measured by the microarray.

†Measures the significance of the difference in expression between the small and large fish.

The biological process level 2 GO category with the largest number of up-regulated sequences in the large Sept. and Dec. fish was ‘metabolic process’ (GO:0008152) while ‘cellular process’ (GO:0009987) contained the highest number of up-regulated sequences in both the small Sept. and Dec. fish (Additional file 13: Table S13). A list of the Agilent probe IDs that correspond to the individual GO Level 2 and Level 3 categories is provided in Additional file 14: Table S14 and Additional file 15: Table S15. However, the category with the second largest number of up-regulated sequences in the large Sept and Dec. fish was ‘cellular process’ and in the small Sept. and Dec. fish was ‘metabolic process’ indicating similarity in function across both size groups. At biological process level 3, the two categories with the greatest number of up-regulated sequences in the large Dec. fish and the large and small Sept. fish were ‘primary metabolic process’ (GO:0044238) and ‘regulation of biological process’ (GO:0050789), respectively (Additional file 16: Table S16). The ‘regulation of biological process’ and ‘primary metabolic process’ categories contained the largest and second largest number of up-regulated sequences in the small Dec. fish.

**Figure 5:**
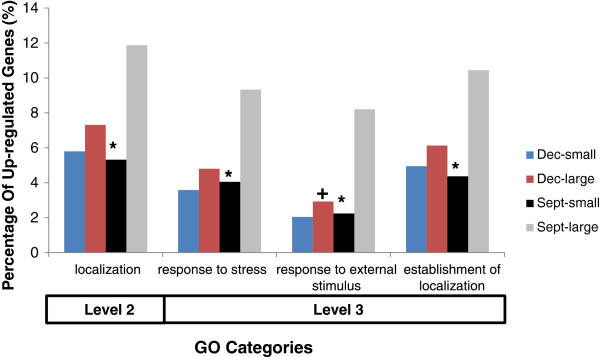
**Significant Gene Ontology term distributions in the white muscle.** GO terms in the white muscle for biological process levels 2 and 3. Significant deviation from the expected number of sequences (total sequences in a category related to total up-regulated sequences), as determined by the G-test for heterogeneity, between the two data sets are marked as significant. Only terms differing by at least 5% are considered significantly different. Asterisks indicate proportionate counts that are significantly higher in large Sept. compared to small Sept fish. Crosses indicate proportionate counts that are significantly higher in large Sept. compared to large Dec. fish. Additional GO terms corresponding to the GO ids shown are listed in Additional file 15: Table S15 and Additional file 16: Table S16.

At biological process GO level 2, significant differences in the observed distribution of GO category gene counts were detected for the ‘localization’ (GO:0051179) category, when the number of up-regulated sequences in the large Sept fish were compared to those in the small Sept. fish (Figure 5). Genes in the ‘localization’ category accounted for 11.8% and 5.3% of the total genes in the large and small Sept. fish, respectively (Additional file 13: Table S13). No differences in the observed gene distributions were found between the small and large Dec. fish or when the large and small fish were compared to those of the same size in different lots. At biological process GO level 3, significant differences in observed counts with at least a 5% difference in the proportion of gene expression were observed for the following GO categories: ‘response to stress’ (GO:0006950); ‘response to external stimulus’ (GO:0009605); and ‘establishment of localization’ (GO:0051234), when the number of sequences up-regulated in large Sept. fish were compared to the sequences up-regulated in the small Sept. fish (Figure 5). In the large Sept. fish, genes in the ‘response to stress’, ‘response to external stimuli’, and ‘establishment of localization’ made up 9.3%, 8.2%, and 10.5% of the total number of genes, respectively. In the small Sept. fish these categories contained 4.1%, 2.2%, and 4.4%, respectively, of the total number of genes assigned to GO categories in this size class. No significant difference in GO distribution was seen between the large and small Dec. fish.

#### Effects of season (lot)

The distribution of differentially expressed genes in GO category, ‘response to external stimulus’ differed significantly between the large Dec. and Sept. fish. Genes in this category made up 8.2% of the total genes in the large Sept. fish and 2.9% of the total genes in the large Dec. fish. No differences in the observed distribution of differentially regulated genes were detected between the small Sept. and Dec. fish. A list of the specific gene probe IDs and their associated gene functions for the GO level 3 categories that show significant differences between large and small Sept. fish and with the largest number of gene differences (i.e., ‘response to external stimulus’, ‘establishment of localization’, and ‘response to stress’), are shown in Additional file 17: Table S17, Additional file 18: Table S18 and Additional file 19: Table S19, respectively. Differences between large fish in Dec. and Sept. lots for ‘response to external stimulus’ are shown in Additional file 17: Table S17. Significant differences in the relative percentage of counts between level 2 and 3 GO categories at the 5% level of differentiation are shown in Figure 5.

### Validation of microarray results with qPCR

**Figure 6:**
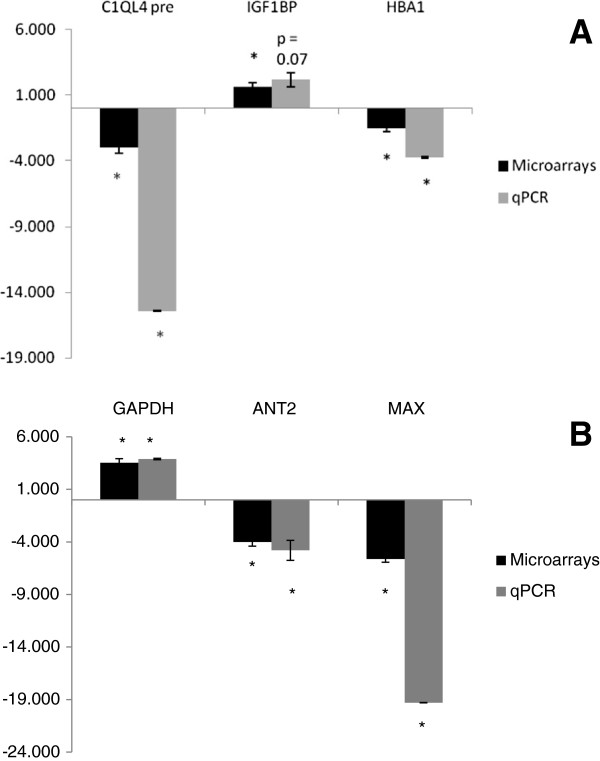
**Quantitative real-time PCR confirmation of differential expression.** In small vs. large fish (i.e. small: large expression ratios shown) in the Sept. lot for differentially expressed genes identified by microarray analysis (left panel) and by qPCR (right panel) in the **A)** liver: complement C1q-like protein 4 precursor (C1QL4), insulin-like growth factor 1 binding protein (IGF1BP), and hemoglobin subunit alpha (HBA1) and **B)** muscle: glyceraldehyde-3-phosphate dehydrogenase (GAPDH), adp/atp translocase 2 (ANT2), max protein (MAX). (*Significantly different when the large fish were compared to the small fish by a t-test, P ≤ 0.05).

The expression of the 3 genes in the muscle and 3 genes in the liver measured by qPCR confirmed the microarray results (Figure 6). Five of the six genes showed statistically significant changes in the small fish compared to the large fish. The remaining gene, *IGF1BP*, had a p value of 0.07 for the qPCR result but showed the same direction of change and was of a similar magnitude as the microarray results. Additional comparisons among other genes with significant expression differences detected in this study were further supported by RNAseq expression profiles of individual large and small fish used in the microarray analysis (unpublished data).

## Discussion

The patterns of differential gene expression between small and large rainbow trout derived from two seasonal spawning groups were more consistent in white muscle than in liver. As a result, the following discussion largely focuses on patterns seen in white muscle. Our initial prediction that genes related to carbohydrate and lipid metabolism would be more highly up-regulated in larger rainbow trout compared to smaller fish sampled in two seasons was not universally observed. Both carbohydrate and lipid metabolic processes are child terms within the primary metabolic process level 3 GO category. This category had a fairly even distribution of up-regulated genes assigned to it, across both size groups and both sampling seasons. Nonetheless, certain white muscle genes such as *lactate dehydrogenase*, were highly up-regulated in large fish from both seasons, as was *pyruvate kinase* expression in Sept. fish. Many lipid regulating genes were also more highly expressed in large fish compared to small fish and these included several apolipoproteins, fatty acid binding proteins, *adipose differentiation related protein, lipoprotein lipase*, and retinol binding protein genes. This latter observation is consistent with the finding that retinoic acid activates myogenesis in zebrafish [34].

Genes for cytoskeleton and myofibrillar components were often differentially expressed between large and small fish but the patterns did not conform to simple expectations. The proportion of differentially expressed genes assigned to cytoskeletal structure, anatomical structure development, biosynthetic process, cellular development process, and organelle organization did not differ significantly between size groupings. In fact, there was a tendency for smaller fish to have a greater proportion of white muscle genes assigned to these categories compared to large fish. The finding that myofibrillar component genes such as *titin, myosin light chain 3*, several actins, *xin-actin-binding repeat containing protein c2, actinin*, several tropomyosins, *myosin heavy chain* and troponin genes were highly up-regulated in smaller fish was unexpected. In contrast, we observed up-regulation for different copies of a *myosin heavy chain, alpha actin, troponin I,* and *myosin light chain 1* in large fish. Previous studies in fish have not determined a clear pattern in *actin* or *myosin* expression relative to growth. Both actin and myosin genes have shown down-regulation in rainbow trout treated with GH [21] and experiencing muscle wastage [7]. Connective tissue components such as keratins, collagens, fibronectins, amyloids, and ictacalcins were also more highly expressed in the larger fish. Thus, the finding that some cytoskeletal organization and assembly genes (e.g. *telethonin*) and connective tissue genes were up-regulated in large fish is consistent with predictions. However, it was also found that different members of the same gene family such as the t-complex assembly proteins were either up- or down-regulated in small fish. Hence, while the expression of individual genes demonstrated relationships with body size, the overlapping nature of their assignments within GO categories restricted the detection of significant differences between the size classes examined.

### Effect of body size across seasons

#### Liver

Contrary to predictions, we did not observe major differences in the expression of the somatotropin axis genes in liver with the singular exception that *IGFBP1* was up-regulated in small fish from both the Sept. and Dec. lots. As IGF-I stimulates muscle growth [35], the binding of a greater portion of the IGF by IGFBP1 in the small fish may have contributed to decreased growth. Indeed, overexpression of *IGFBP* in grass carp, *Ctenopharyngodon idella,* embryos causes phenotypes that resemble hypoxia-induced developmental delay and growth retardation [36]. In addition, the increased expression of IGF binding proteins might reflect increased protein catabolism as shown in Atlantic salmon, *Salmo salar*[37], which could also be a contributing factor in decreased growth in the small fish. While other studies have identified expression differences in the liver for genes in a variety of categories, including lipid and carbohydrate metabolism, immunity, transcription, and growth regulation [21,26], they compared GH-treated fish to control or GH-transgenic and domestic populations to wildtype fish. Thus, our results may not be directly comparable to these previous studies given that we only examined growth in domesticated rainbow trout reared in typical and unaltered culture conditions.

The limited number of instances where large fish showed up-regulation of genes compared to small fish in liver restricted our ability to find consistent patterns across seasons. In fact, our search for size-specific consistency in liver expression profiles across seasons was largely limited to patterns observed in smaller fish. In addition to *IGFBP1*, levels of *zinc finger protein 568* and *glutamine synthetase* (GS) expression were elevated in small fish sampled from both seasons. This supports our prediction that genes for signaling and transcriptional activity would be up-regulated in small fish. Although GS does not directly modulate RNA polymerase II activity in a fashion similar to zinc finger proteins, GS modulates glutamate to glutamine pools in muscle and liver. Glutamate:glutamine ratios can influence muscle homeostasis, via the regulation of metabolism in response to intracellular energy states, and these ratios can vary in response to stress and nutritional levels [38].

#### White muscle

Classification of gene expression profiles in muscle also revealed associations between blood and energy production, calcium metabolism, and stress and immune function between large and small fish. Genes involved in blood and energy production and immune function were up-regulated in large fish relative to small fish while the opposite was true for genes related to calcium metabolism. Genes involved in the clotting process (*fibrinogen gamma polypeptide;* 4 fibrinogen sequences*, C1 inhibitor)*[39] and energy production (*HIG1 domain family member 2a, ADP/ATP translocase 2)*[40,41] were up-regulated in large fish from at least one of the lots. Salem et al. [30] also reported that the expression patterns of several metabolic genes including, ATP and ATPase were associated with faster growth in rainbow trout muscle. The observation that a number of genes related to immune function were up-regulated in the large fish suggests that enhanced growth may be associated with enhanced immune function. *Liver-expressed antimicrobial peptide 2,* which is involved in innate immunity [42], *flavin reductase*, a broad specificity *oxidoreductase*[43], and *fucolectin-4 precursor*[44], were all highly up-regulated in the large Dec. fish. *C-type lectin* was highly-up-regulated in the large Sept. fish and belongs to a super-family of proteins with a variety of functions including involvement in cell adhesion, receptor binding, and immunity [45]. The suggestion that immune function plays an important role in growth is supported by the concordance of expression profiles of such genes between GH-transgenic and domestic rainbow trout (fast growth) when compared to wildtype fish (slower growth) [26].

In contrast, many genes related to calcium metabolism were up-regulated in small fish relative to large fish. For example, *parvalbumin 2* and *parvalbumin beta-2,* which bind calcium ions and are involved in muscle relaxation after contraction, were up-regulated in the small fish suggesting that genes controlling calcium homeostasis also regulate growth in fish. Overexpression of parvalbumins (PARV) can lead to faster quick-twitch muscle relaxation times, but paradoxically, have also been coupled to lower mitochondrial densities in slow-twitch muscle groups [46,47]. Lower PARV levels also indicate enhanced mitochondrial and metabolic flux capabilities as evidenced by inverse associations between SIRT1/PGG-1α activities and PARV levels [48]. Therefore, it is possible that the larger fish in our study have higher mitochondrial densities and metabolic capabilities than smaller fish. Increased expression of certain IGFBP genes also appear to directly elevate levels of intracellular calcium and are linked to repressed cell growth [49]. This suggests a more direct link between the elevated *IGFBP* levels we observed in liver with the elevated PARV gene expression levels detected in muscle.

Our data provide interesting insights into metabolic functional links related to some of our initial predictions. The observation that smaller fish have enhanced levels of *glyceraldehyde-3-phosphate dehydrogenase* (GAPDH) expression suggests that mTOR activity might be suppressed in smaller fish as observed in other species [50]. GAPDH is a key regulator of mTOR expression through its regulatory affinities to Rheb, an activator of the mTOR cascade [51]. Buller et al. [52] observed that GLUT1 activation can regulate glycolytic flux and noted that enhanced GAPDH expression depresses mTOR activation, presumably by diminishing the binding interactions between Rheb/mTOR when GAPDH levels are elevated. mTOR levels are also known to be lower in dwarf Icelandic Arctic charr (*Salvelinus alpinus*) compared to fish with more typical growth patterns [50]. However, the current findings are also somewhat paradoxical, as GAPDH levels may also be expected to be elevated in large fish given that carbohydrate metabolic flux is expected to be greater since type II glycolytic muscle mass (i.e., white muscle tissue) represent a greater proportion of body mass in larger fish [53]. Since GAPDH is the rate-limiting enzyme in glycolytic flux [54], enhanced carbohydrate based metabolism should be reflected in higher GAPDH levels in larger fish. GAPDH activity was observed to be significantly higher in gilthead sea bream (*Sparus aurata*) under normal growing conditions and was considered a signature gene of altered metabolism following environmental perturbations with feeding regime [55]. As mentioned previously, we did detect significantly enhanced *lactate dehydrogenase* and *pyruvate kinase* expression levels in larger fish supporting the prediction that glycolytic flux derived energy acquisition is enhanced in larger fish. It is possible that duplicated forms of GAPDH, not detected with the probes available on the current microarray, may show enhanced expression in larger fish and therefore more research is required.

### Heterogeneity among GO categories

The two seasonal lots of fish did not show significant shifts in gene ontology categories for liver tissue. While this does not mean that all genes are uniformly expressed in the two seasonal lots, it does suggest that functionally equivalent classes of liver genes are similarly regulated during times of varying photoperiod, at least within the experimental families we surveyed. This may be a consequence, in part, of temperature-induced maintenance of metabolic rates, as the fish were kept at water temperatures that fluctuated very little throughout the year. More dramatic differences would possibly be observed in nature, especially at the juvenile stage when young salmonids inhabiting a fluvial environment may experience more dramatic fluctuations in temperature.

Differential white muscle gene counts were evident between large and small fish within 4 different GO categories. Small and large Sept. fish showed significant differences in the level 2 ‘localization’ category. At level 3 the number of genes assigned to GO categories within the ‘response to external stimulus’, ‘establishment of localization’ and ‘response to stress’ level 3 GO groupings showed the greatest number of significant differences between large and small fish in the Sept. lot. Significant differences between large fish from the two seasonal lots were also evident within the ‘response to external stimulus’ category. These findings suggest that differences in expression levels between large and small fish may be more pronounced during periods of growth retardation compared to periods of acceleration, given that most of the significant GO categories were evident in the Sept. lot. The fish from the Sept. lot were sampled during a period of low specific growth rate at the winter solstice time period (Figure 3). The following discussion will be restricted to these level 3 categories.

#### Response to external stimulus - level 3

As predicted, large fish in the Sept. lot can be characterized as having a greater number of up-regulated genes related to cytoskeletal structure compared to small Sept. fish and these include two *telethonin* sequences*, fibronectin precursor,* and *beta-enolase. Telethonin* is a muscle regulating factor [56], *beta-enolase* has a function in striated muscle development [57], and *fibronectin* is involved in muscle growth [58]. Large Sept. fish also had a greater number of genes related to angiogenesis and haemopoeisis up-regulated in this category (e.g., *fibrinogen alpha chain*, *fibrinogen gamma chain* (3 probe IDs), *coagulation factor ii precursor*, *albumin precursor*, and *antithrombin-iii precursor* (2 probe IDs), compared to small Sept. fish (*platelet glycoprotein 4*).

#### Establishment of localization – level 3

We also observed differential expression patterns of *calmodulin* and the calmodulin induced kinases that were contrary to expectations. *Calmodulin, calcium calmodulin-dependent protein kinase ii delta 2*, and *calcium calmodulin-dependent protein kinase iv* were all up-regulated in the small Sept. fish. Typically, c*almodulin* genes and their associated kinases are related to increased growth and anabolic functions and thus their expression levels are expected to be higher in faster growing organisms. For instance, c*alcium calmodulin-dependent protein kinase i delta* is highly expressed in the skeletal muscle of zebrafish embryos [59] and c*alcium calmodulin-dependent protein kinase ii delta 2* is involved in skeletal muscle regeneration [60] and the regulation of vascular smooth muscle migration [61,62] in mammalian models. Moreover, *calcium calmodulin-dependent protein kinase iv* has been reported to stimulate hypertrophic muscle growth through the regulation of transcription factors associated with the expression of red muscle and the biogenesis of skeletal muscle mitochondria [63,64]. The finding that the calcium calmodulin dependent kinases are also more highly up-regulated in small fish is in accordance with the observation of increased calcium-dependent metabolism in small fish as evidenced by their elevated levels of PARV.

Expression of *calcium calmodulin-dependent protein kinase iv* within the skeletal muscle remains controversial. During zebrafish embryogenesis *calcium calmodulin-dependent protein kinase iv* was not expressed in the skeletal muscle but was instead involved in the development of the brain and neurons [65]. In humans, *calcium calmodulin-dependent protein kinase iv* is undetectable [66] but it is detectable in mice [67]. Strict anabolic functions for this complex are also controversial as *calmodulin* has also been shown to be a regulator of apoptosis [68], with *calcium calmodulin-dependent protein kinase ii* identified as being important for apoptosis maintenance [69]. Seales *et al.*[70] have also shown that calmodulin signaling also regulates increased osteoclastogenesis. Current evidence does not suggest that *calcium calmodulin-dependent protein kinase iv* is linked to apoptotic processes. Nevertheless, the pleiotropic nature of this complex clearly requires more research, and linking the activity of the genes to reduced growth may indicate coupling to increased apoptotic activation.

The majority of genes related to transcription, translation, and protein production were up-regulated in the small Sept. fish. These included *transcription factor*, *membrane-bound transcription factor site 1*, *splicing arginine serine-rich 11*, *protein fat-free homolog*, *60s ribosomal export protein nmd3, 60s ribosomal protein l11*, and *ras-related protein rab-2a.* These findings support prediction (3), albeit based upon gene expression levels in white muscle tissue, whereas the up-regulation of signaling genes in small fish were previously based upon liver microarray findings [29]. Genes involved in protein degradation such as *selenoprotein S, der1-like domain member 2,* and *ubiquitin C* also showed up-regulation in the small Sept. fish. This suggests that the increased protein production indicated in the small fish may not translate into finished proteins (see Discussion in following section). In contrast, genes related to protein production or assembly showed up-regulation in the large Sept. fish (e.g., *Ap-1 complex subunit gamma-1,* which is involved in protein sorting at the golgi [71], and *ribosomal protein s13* which is involved in protein biosynthesis [72], *telethonin* which is involved in sarcomere assembly [56]), as did genes related to lipid metabolism (e.g., retinol binding proteins, fatty acid binding proteins, and *apolipoprotein a-i*) and blood production (e.g., hemoglobin alpha and beta units). These findings support prediction (1) and partially support prediction (2), given that the myofibrillar component genes of muscle assembly were more up-regulated in the small fish (see Discussion above) which was not expected.

#### Response to stress - level 3

Small Sept. fish show up-regulation for a higher number of genes related to apoptosis compared to large fish despite the fact that as a portion of total genes differentially expressed, the ‘response to stress’ category contained a higher proportion of genes in large fish. Differences were apparent in the make-up of genes contributing to this GO category between the size classes. Genes up-regulated in large fish could be categorized as enhancers of innate and acquired immune function (e.g., *Complement component c3, complement c3-like*, *complement component c9,* and Ca^2+^-dependent *complex c1r c1s subunit*[73]), transcriptional activation, such as *max protein* and *pyruvate kinase*, and regulators of oxidative stress (e.g., *glutathione peroxidase*). Additionally, inspection of Additional file 9: Table S9 reveals that genes such as *thioredoxin* and *glutathione-s-transferase* were up-regulated in the large but not the small fish supporting the contention that large fish are better able to handle oxidative stress. Conversely, genes up-regulated in the small Sept. fish may be characterized as having enhanced apoptotic responses to stress (i.e., increased expression of *proteasome subunit alpha type-6*, *proteasome subunit alpha type-5*, *ubiquinone biosynthesis protein coq7 homolog*, *26 s proteasome non-ATPase regulatory subunit 3*, *ubiquitin C*, *apoptosis enhancing nuclease, proteasome (macropain) 26 s 6*, *26 s protease regulatory subunit 8*, and *DNA damage-binding protein-1* [DDBP1]). However, with DDBP1 its associated substrate ligand is linked to the degradation of a cell cycle inhibitor [74], and thus up-regulation of this gene would be expected to increase cell growth. Inhibition of *26s proteasome* promotes muscle growth in rats [75] and therefore higher activation in smaller fish may be coupled to decreased growth which is consistent with our findings.

Genes of mixed function, such as those related to protein degradation regulation such as *heat shock protein hsp 90-alpha* (hsp90a), *selanoprotein s* (SelS), and *der1-like domain member 2,* are also up-regulated in small Sept. fish. Interestingly all three of these proteins may have regulatory interactions in that hsp90a is required for SelS and general selanoprotein synthesis, and SelS interacts with der1 in shuttling mis-folded proteins across the endoplasmic reticulum membrane towards eventual ubiquination and proteasome degradation [76]. The observation that large Sept. fish express higher levels of *HIG1 domain family member 2a*, *glutathione peroxidase*, and C*1 inhibitor,* which all act to prevent apoptosis and protease degradation, supports the idea that small fish are experiencing more protein turnover, possibly as a response to stress, than large fish. In this regard, however, it is also important to highlight that large fish also express much higher levels of genes related to catabolism such as *cathepsins b, d, m,* and *l*, and several genes related to chitinase, collagenase, aminopeptidase, serine protease, lysozyme, and matrix metalloproteinase activities. This indicates that catabolic activities appear to be somewhat higher in large fish and may reflect upon their increased metabolic activity. These results are similar to the up-regulation of genes related to carbohydrate, lipid, and amino acid metabolism coupled with the up-regulation of proteolysis in GH-transgenic and domestic fish compared to wildtype fish [27].

### Effect of season (lots)

The larger Dec. fish showed differential expression of more genes in white muscle and fewer genes in the liver than the Sept. fish. While underlying genetic differences between the parents used to produce the two lots could contribute to the observed differences in gene expression, it is also important to consider environmental effects. While the lots were exposed to the same water, temperature, dissolved oxygen, and nutrition, they experienced a different photoperiod regime at the time tissues were sampled for RNA analysis. The Sept. lot was sampled at a time period approaching winter solstice, a period of declining growth, while the Dec. lot was experiencing increasing daylight approaching spring equinox, a period of increasing growth. The effect of season on the different growth rates between the lots is supported by the TGC profiles (Figure 3). As expected, the TGC values were either constant (Dec. lot) or declining (Sept. lot) at the time of the December 2009 sampling despite the fact that fish numbers had recently been reduced through culling. All previous samplings when tank densities were reduced were followed by a large increase in TGC values in the subsequent sampling period, simply because the fish had more environmental space in which to grow. This was not observed in the December sampling in either lot, and therefore photoperiod clearly had a major influence on the growth rates in these fish at that time of the year.

Photoperiod regimes have major influences on growth rates in fishes but these are not always in predictable directions and may be species-specific. Although increased light exposure produces increased growth [77] in rainbow trout, varied effects (increase or decrease) have been observed in Atlantic cod (*Gadus morhua*) [9,78]. Our findings indicate that the induced changes in liver gene expression may be more multifarious between large and small fish and may be confounded by varying photoperiod regime. In contrast, the vast majority (93%) of gene probe IDs that demonstrated significant up-regulation in white muscle were consistent in either small or large fish across seasons. Large and small fish from the Sept. lot showed a greater number of differences in gene expression in the liver compared to the Dec. lot, while the converse was true for white muscle. This could be explained by the initiation of spring growth in the Dec. lot, as suggested by changes in the TGC at the time of sampling, leading to increased variability in white muscle expression levels between fish of different sizes. This indicates that expression levels differ on a seasonal basis with fewer differences between large and small fish during reduced growth in the fall, and greater differences coupled to increasing photoperiod in the spring.

## Conclusion

These results indicate that sampling season can have a significant impact on the expression of genes related to the growth process in rainbow trout, with the TGC showing seasonal changes in both lots. In muscle, the large fish from the Dec. lot showed increased expression of genes related to muscle and connective tissue growth while genes related to blood production and the immune system were up-regulated in the large Sept. fish. This pattern may correspond to the differences in the growth profiles of the two lots. The Dec. fish were entering a period of increased growth while the Sept. fish were entering a phase of lower growth and possibly increased stress when they were sampled. These differences highlight the importance of sampling season in interpreting findings from gene expression studies.

A greater number of genes in white muscle demonstrated consistent associations with fish size regardless of sampling season, suggesting they may be better predictors of the growth response in rainbow trout compared to those in liver. Differences between large and small Sept. fish in the ‘response to stress’ category for white muscle tissue, indicate an up regulation of genes related to innate and acquired immunity in the large fish, while genes related to apoptosis have higher expression levels in the small fish. Depression of immune function genes and lower levels of oxidative stress genes in small fish suggest diminished stress handling capabilities. Genes within the ‘response to external stimulus’ and ‘establishment of localization’ categories also showed major expression differences between large and small Sept. fish. These categories show up-regulation of genes related to transcription, translation, and protein production in the small fish. However, since genes related to apoptosis were also up-regulated in the small fish, this suggests that higher cellular turnover rates may diminish the expected physiological effects of increased transcription, translation, and protein production. Genes related to blood and energy production showed up-regulation in the large fish compared to the small fish indicating an increased energy demand in the muscle of the large fish. In contrast to muscle tissue, *IGFBP1* was the only gene with highly significant and consistent up-regulation in the liver across seasons.

## Method

### Fish husbandry

**Figure 7:**
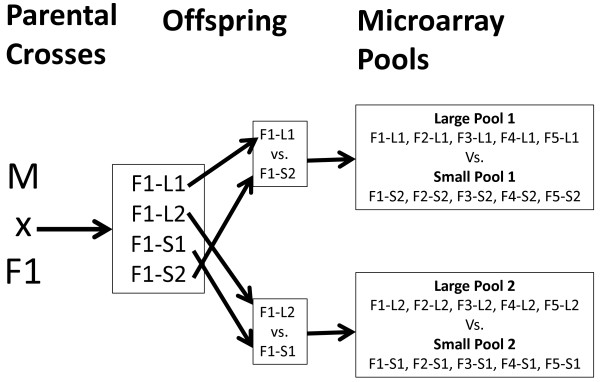
**Parental crosses used to produce the families used for the microarrays.** In each season a single male (M) was crossed with five females (F); a single cross is depicted. The two largest (L) and two smallest fish (S) were selected from each female’s offspring pool. The largest fish (L1) from each female parent was pooled and compared to a pool of the second smallest fish (S2). A pool of the second largest fish from each female was compared a pool of the smallest fish from each female.

Rainbow trout from the Lyndon commercial strain of rainbow trout (Lyndon Fish Hatcheries Inc., R.R. #1, New Dundee, ON) were reared at the Alma Aquaculture Research Station (AARS) using a protocol approved by the Animal Care Committee at the University of Guelph following the guidelines of the Canadian Council of Animal Care. Adults from the same strain but spawning in different seasons were selected as parents for the seasonal lots. Five paternal half-sib families were created by crossing 5 females with a single male on September 18, 2008 (referred to as Sept. lot) (Figure 7). A second set of paternal half-sib families was created using 5 later spawning females and a different male on December 10, 2008 (referred to as Dec. lot). Paternal half-sib families were created to facilitate a concurrent quantitative trait locus (QTL) study for growth-related traits.

All the progeny within a lot were reared together in a common environment at 8 to 10°C under a natural photoperiod from hatching to the end of the experiment. They were fed a commercial salmonid ration containing 44-55% protein and 15-22% fat depending on life stage, which corresponded to the thermal growth coefficients devised for rainbow trout (1.5-3% of body weight daily) [79]. Tank densities were adjusted periodically according to optimal rearing densities and feeding schedules were regulated by the bulk biomass of the fish (Figure 3). Mortalities were recorded daily and cumulative numbers for each weighing interval are depicted in Figure 3. The numbers of fish in the Sept. and Dec. lots prior to the first mortality screening on Dec. 2, 2008 and Feb. 3, 2009, respectively, were 438 and 309. Fish were bulk weighed biweekly during the initial growth period until April 14, 2009, and then every 28 days until completion of the experiment. Feed rations were adjusted according to fish average weights and water temperature. Water temperatures ranged from 8-11°C throughout the experimental period (averaging 8.5°C) and water came from deep underground wells. Fish from each lot were re-located to tanks of increasing size (i.e., 0.7 m diameter; 1 m; and 2 m) as they grew. Mortalities/interval, weight-specific growth rates (SGR),

$$\mathrm{SGR}=\left(\mathrm{lnwt}2-\mathrm{lnwt}1\right)/\left(t2-t1\right)\times 100$$

where wt2 = mean weight of the fish at time t2 (days) and wt1 = mean weight of the fish at time t1 (days)

thermal growth co-efficient (TGC) [79], and tank densities were calculated throughout the experimental period (Figure 3) and no disease outbreaks occurred. Mortalities were minimal throughout with the largest percentage occurring during the early rearing stages. Both the Sept. and Dec. lots had similar mortality profiles with the largest values observed following the first bulk weighing period. Fish were fasted for two days before weighing. Fork length (nearest mm) and weight (nearest g) were measured at approximately 15 months of age - December 2, 2009 for the Sept. lot and February 23, 2010 for the Dec. lot. All fish were reproductively immature at the time of sampling. Gonadal inspection revealed only thin gonadal strands in the head kidney region and thus all the fish were actively growing juveniles. Weight differences among the half-sib families within each lot were compared using an ANOVA and Tukey’s post-hoc test based on the results of a Levene’s test for equality of variance.

### Tissue collection

Samples of liver and white muscle were collected from 50 to 55 fish within each half-sib lot which were purposefully size-selected to represent the fastest and slowest growing fish in each lot. The tissues were placed in an RNA preserving solution (3.75 M (NH_4_)_2_SO_4_, 10 mM EDTA, 25 mM Na_3_C_6_H_5_O_7_, adjusted to pH 5.2) and then stored at −80°C until use. White muscle was excised immediately below the dorsal fin and well above the lateral line to avoid contamination with red muscle. A small square plug measuring less than a 1 cm was excised and the surface skin removed. Small fragments of tissue (~100 mg sections) from below the dermal connective tissue bundle (i.e., muscle myofibrils only) were placed in the RNA preserving solution.

### Parentage and sex determination

DNA was extracted from the liver samples using a standard phenol chloroform protocol [80]. The purity and concentration of the DNA was quantified using a Nanodrop 8000 spectrophotometer (Thermo Scientific, Waltham, MA) and samples were stored at −20°C. The archived parental tissue was genotyped for variation in microsatellite loci to identify the parentage of the progeny. Markers BX887563, Omm1220, Omm5147, and Omy1212UW were used to identify the parentage of the progeny in the Sept. lot and markers Omm1054, Omm1087, Omm1088, and Omm5156 were used to identify parentage in the Dec. lot [81]. Polymerase chain reaction (PCR) mixtures were made in 7 μL volumes (2.6 ng genomic DNA · μL^-1^, 1× PCR buffer, 0.125 mM dNTP, 1.5 mM MgCl_2_, 1.5 nM BSA, 0.3 μM of each forward or reverse marker primers labeled with tetrachloro-6-carboxy-flourescent, 0.021 U μL^-1^ Taq DNA polymerase). PCR conditions began with initial denaturation (95°C for 10 min), followed by 35 cycles of denaturation (95°C for 1 min), annealing (30 s), and extension (72°C for 30 s), and concluded with final extension (72°C for 5 min). An annealing temperature of 58°C was used for all primers except BX887563, which used 54°C.

The sex of the progeny was determined using the rainbow trout Y-specific marker OmyY1 [82]. Polymerase chain reaction (PCR) mixtures were made in 20 μL volumes (3 ng genomic DNA · μL^-1^, 1× PCR buffer, 0.2 mM dNTP, 1.75 mM MgCl_2_, 0.2 μM of each forward or reverse marker primers, 0.01 U μL^-1^ Taq DNA polymerase). PCR conditions began with initial denaturation (95°C for 5 min), followed by 30 cycles of denaturation (95°C for 30 s), annealing (58°C for 1 min), and extension (72°C for 1 min), and concluded with final extension (72°C for 10 min).

### RNA Isolation

Total RNA was isolated from the liver and white muscle of the 2 largest and 2 smallest fish by weight in each half-sib family for both the Sept. and Dec. lots. TRIzol (Invitrogen, Carlsbad, CA) extractions were performed according to the manufacturer’s instructions and the resulting RNA concentrations were determined using a Nanodrop8000 spectrophotometer. The samples were stored at −80°C until future use. The presence of distinct 18S and 28S rRNA bands after agarose gel electrophoresis was used to confirm that the RNA was not degraded. The extraction process was repeated until a minimum of 40 μg of RNA was collected from each sample. Since large and small fish are growing at different rates it is possible that rRNA makes up a different proportion of the total RNA in the small and large fish. This would result in different amounts of mRNA within a unit volume of total RNA. Therefore, we avoided this potential bias by using purified mRNA. Prior to cDNA synthesis, mRNA was purified from the total RNA using μMACS mRNA Isolation Kits – Small Scale (Miltenyi Biotec Inc, Auburn, CA) according to the manufacturer’s instructions.

### Gene expression

Gene expression levels were profiled for liver and muscle using 0.5 μg pools of mRNA composed of 0.1 μg of mRNA from each of five individuals (Figure 7). Because of the size differences between the full-sib families within lots we could not compare the largest fish to the smallest fish within each lot. This would have resulted in the majority of large fish coming from one or two families and the same would be true of the small fish. To avoid family bias, one mRNA pool was created by pooling mRNA from the largest fish in each of the 5 half-sib families within the Sept lot (L1). This process was repeated for the second largest (L2), smallest (S1), and next to smallest (S2) fish in each of the Sept lot half-sib families. Weight differences between the sets of large and small fish that made up the pools are shown in Table 3. Gene expression levels of the L1 fish were compared to those of the S2 fish and L2 fish were compared to S1 fish to provide approximately equal weight differences between the large and small fish in the two comparison groups. The same pooling strategy and comparisons were made for the Dec. lot.

**Table 3:** Average weights of the pooled fish used for the microarray (g)

	**Sept.**			**Dec.**		
	**Large**	**Small**	**Difference**	**Large**	**Small**	**Difference**
L1 vs. S2	489	125	364	665	262	403
L2 vs. S1	467	78	389	560	183	377

Gene expression was profiled using an Agilent salmonid microarray (catalog number - G2519F-020938) containing cDNAs from 43,663 genes selected from Atlantic salmon expressed sequence tag databases (Aglient Technologies, Mississauga, ON). This chip allows quantification in expression of genes involved in muscle growth such as *GH, IGF-I* and *II*, *actin,* and *myosin.* Approximately 0.2-0.3 μg of cDNA was prepared from the mRNA using a SuperScript® Plus Direct cDNA Labeling System with Alexa Fluor® aha-dUTPs (Invitrogen, Burlington, ON) and labeled with either Alexa Fluor 555 or Alexa Fluor 647. Briefly, the mRNA was reverse transcribed using an anchored oligod(T)20 primer and random hexamers in cDNA synthesis reactions that incorporated Alexa Fluor-labeled nucleotides following the manufacturer’s specifications. The concentration and dye incorporation rates of the cDNA were calculated using a Nanodrop 8000 spectrophotometer. Samples were stored at −20°C until hybridization, which occurred on either the same or the following day.

The cDNA was hybridized overnight to the microarrays according to the hybridization protocol described in Two-Color Microarray-Based Gene Expression Analysis (Agilent Technologies). Dye flips were included to compensate for any dye effects. Because air ozone levels could affect the Alexa Fluor 555 signal, we adopted the Agilent Ozone protection protocol following the manufacturers’ specifications to prevent degradation. This was followed by washing to remove any cDNA that had not hybridized to the array. The microarrays were scanned on a GenePix 4200A scanner (Axon Instruments, Weatherford Texas). Genepix Pro 6.1 software was used to process images, align the spots, integrate the Genepix Array List file with the microarray images, and quantify the spots. The arrays were subject to manual review and all unacceptable spots were flagged and excluded.

Genespring GX 7.3.1 (http://www.genomics.agilent.com) software was used to analyze the microarray data. Data were preprocessed by removing data points where the signal intensities ratio of both channels was less than a baseline threshold value of 0.1. Lowess (locally weighted scatter plot smoothing) normalization was applied to the data to normalize the spot intensity from various replicates. Spots that did not have a value for all of the replicates within a lot were removed from the analysis. Cross-array lowess normalizations were performed by the software and for each spot, t-statistic, p-value (probability), and the ratio between the two dyes were calculated by the Genespring software. Following corrections spots with a minimum of 1.2-fold change or more were considered differentially expressed (p ≤ 0.05). Microarray data were submitted to the NCBI Gene Expression Omnibus [83] under the submission number GSE42584.

Target gene lists obtained from the significance analysis were re-annotated using Blast2GO (http://www.blast2go.com/b2ghome) to assign GO terms to the differentially expressed microarray sequences. This software applies an automated BlastX alignment of the sequences to the NCBI non-redundant database. The BlastX expectation value threshold was set to 1.0E-6 whereas all other parameters were set to the default values. If Blast2GO was unable to assign GO terms to a sequence, manual evaluations were considered using AmiGO (http://amigo.geneontology.org/cgi-bin/amigo/go.cgi) assignments. A heterogeneity G-test (http://www.uoguelph.ca/~rdanzman with links to the software module) was used to compare the number of genes differently expressed in the GO categories at GO biological process levels 2 and 3 (GOslim generic categories) between groups using a backwards elimination procedure, using counts assigned within the Blast2GO program to the various level 2 and 3 GOslim categories. If significance was detected, the software reported the tabular category with the greatest heterogeneity. This category was removed from the analysis and the remaining categories were re-analyzed in a stepwise fashion until no significant differences remained. The GO categories removed at each step (i.e., containing significant differences in gene expression levels between the groups compared), are reported in Additional files provided. However, in several cases, the number of genes assigned to a grouping was small in number, and often with an absence of gene assignments within 1 of the 2 cells being compared. Therefore, only GO categories that differed by more than 5% (i.e. total proportion of all GO terms assigned) of all the genes assigned across all groupings were considered and discussed. The comparisons evaluated by the heterogeneity G-tests were: large vs. small fish within each seasonal lot; large vs. large fish from different seasonal lots, and small vs. small fish from different seasonal lots. This yielded 4 contrasts where two tested for the effects of size within a season and two tested for seasonal effects on fish of the same size category.

### Real time PCR

Real time PCR was used to validate the expression of three genes in each of liver and muscle that were differently expressed in both the Sept. and Dec. lots. The one exception was *complement C1q-like protein 4 precursor,* which showed differential expression in the Sept. lot. It was included because other complement C1q-like protein genes showed differential expression in the Dec. lot. mRNA (0.1 μg/fish) from the fish in the Sept. lot used in the microarray experiment was used to make cDNA using multiscribe reverse transcriptase (ABI, Burlington, ON). Twenty fish were used for the liver analysis and 18 fish for the muscle analysis. Real-time PCR primers were designed from gene sequences (Table 4) using Primer Express 3.0 software (ABI). *Beta-actin* was chosen as an endogenous control for normalization of the real-time PCR analysis as this gene did not show differential regulation in the microarray experiments. Quantitative PCR was performed in triplicate for each cDNA sample on a StepOne Plus Real Time PCR System (ABI) using PerfeCTa^(R)^ SYBR^(R)^GreenFastMix^(R)^ (Quanta Bioscience, Gaithersburg, MD) with 15 μl reaction volumes containing 200 nM of each primer. Threshold lines were adjusted to intersect amplification lines in the linear portion of the amplification curve and cycles to threshold (Ct) were recorded. Standard curves for each gene and the reference gene were constructed using serial dilutions based on pools of mRNA from eight of the individual fish used for qPCR. PCR data were analyzed using the method described by Bookout and Mangelsdorf [84]. Briefly, the amount of target gene was determined from the appropriate standard curve and was divided by the amount of reference gene to obtain a normalized target value. Mean differences in expression levels were reported as relative fold changes. This was done by designating the control group (large fish) as a calibrator and dividing the mean of treatment group (small fish) by the mean of the calibrator. Outliers, determined as being greater or less than 1.5x the inter-quartile range from the upper or lower quartiles respectively, were removed. Two individuals were removed from each of the liver samples and one individual was removed from each of the muscle samples. A t-test was used to determine if the large and small fish within each lot showed significant differences in mean gene expression levels.

**Table 4:** Genes used for qPCR in the liver and white muscle to confirm the results of the microarray

**Liver**		
**Gene name**	**Forward primer**	**Reverse primer**
IGF-binding protein 1 precursor (IGF1BP)	5′-CCAAGCAGTGTGAGTCGTCTCT-3′	5′-CCGGAATCTTCTTCCCATT -3′
Complement C1q-like protein 4 precursor (C1QL4 pre)	5′-GCAGGCACTGAAAGACATTCC-3′	5′-TGCCTTTTGGAGTCCATTGC-3′
Hemoglobin subunit alpha-1 (HBA1)	5′-TGGATTGTAAACACATCGTTCGT-3′	5′-CCACTATCAGTAGCACTGTCAAAGC-3′
**Muscle**		
Max protein (MAX)	5′-TGTCGATGGTCTGGGACAACT-3′	5′-TCCCCGCCCTGGATTT-3′
Glyceraldehyde-3-phosphate dehydrogenase (GAPDH)	5′-TATGACTCCACCCACGGTGTT-3′	5′-TGCCAATGATCAGCTTTCCA-3′
ADP/ATP translocase 2 (ANT2)	5′-TTTCCATTGAGCCCCTTTCA-3′	5′-ACGACCGTGGAAATGTTTGTAA-3′
**Control**		
Beta-actin*	5′-CAGCCCTCCTTCCTCGGTAR-3′	5′-AGCACCGTGTTGGCGTACA-3′

*Beta-actin serves as the control gene for both the liver and muscle genes.

## Competing interests

The authors state this research is free of conflicts of interests.

## Authors’ contributions

This study was conceptualized by RGD and MMF. ALK conducted the microarray and PCR studies and analysis with advice from RGD and MMF. ALK drafted the manuscript with input from MMF and RGD. All authors read and approved the final manuscript.

## Supplementary Material

Additional file 1: Table S1 Genes up-regulated in the liver of large rainbow trout compared to small rainbow trout. Click here for file

Additional file 2: Table S2 Genes up-regulated in the liver of small rainbow trout compared to large rainbow trout. Click here for file

Additional file 3: Table S3 Genes of unknown function up-regulated in the liver of large rainbow trout compared to small rainbow trout. Click here for file

Additional file 4: Table S4 Genes of unknown function up-regulated in the liver of small rainbow trout compared to large rainbow trout. Click here for file

Additional file 5: Table S5 Gh-test Results for the liver at GO level 2. Click here for file

Additional file 6: Table S6 Agilent Sequences Corresponding To Each GO ID For GO Levels 2 And 3 In The Liver Of The Sept. Fish. Click here for file

Additional file 7: Table S7 Agilent Sequences Corresponding To Each GO ID For GO Levels 2 And 3 In The Liver Of The Dec. Fish. Click here for file

Additional file 8: Table S8 Gh-test Results for the liver at GO level 3. Click here for file

Additional file 9: Table S9 Genes up-regulated in the white muscle of large rainbow trout compared to small rainbow trout. Click here for file

Additional file 10: Table S10 Genes up-regulated in the white muscle of small rainbow trout compared to large rainbow trout. Click here for file

Additional file 11: Table S11 Genes of unknown function up-regulated in the white muscle of large rainbow trout compared to small rainbow trout. Click here for file

Additional file 12: Table S12 Genes of unknown function up-regulated in the white muscle of small rainbow trout compared to large rainbow trout. Click here for file

Additional file 13: Table S13 Gh-test Results for white muscle at GO level 2. Click here for file

Additional file 14: Table S14 Agilent Sequences Corresponding To Each GO ID For GO Levels 2 And 3 In The White Muscle Of The Sept. Fish. Click here for file

Additional file 15: Table S15 Agilent Sequences Corresponding To Each GO ID For GO Levels 2 And 3 In The White Muscle Of The Dec. Fish. Click here for file

Additional file 16: Table S16 Gh-test Results for white muscle at GO level 3. Click here for file

Additional file 17: Table S17 Genes Up-regulated in White Muscle in Large Rainbow Trout compared to Small Rainbow Trout within the GO ‘response to external stimulus’ category. Click here for file

Additional file 18: Table S18 Genes Up-regulated in White Muscle in Small Rainbow Trout compared to Large Rainbow Trout within the GO ‘establishment of localization’ category. Click here for file

Additional file 19: Table S19 Genes Up-regulated in White Muscle in Large Rainbow Trout compared to Small Rainbow Trout within GO ‘response to stress’ category. Click here for file
