# Supplementary material for: Differential gene expression in small and large rainbow trout derived from two seasonal spawning groups
Source: BMC Genomics. 2014 Jan 22;15:57. doi: 10.1186/1471-2164-15-57 (PMC3931318; doi:10.1186/1471-2164-15-57)
Supplement: Additional file 1: Table S1 — Genes up-regulated in the liver of large rainbow trout compared to small rainbow trout. [file 1471-2164-15-57-S1.docx]

| **Supplementary Table 1: Genes up-regulated in the liver of large rainbow trout compared to small rainbow trout** | | | |
| --- | --- | --- | --- |
| **Gene Name** | **Gene Number** | **Fold change^a^** | **p-value^b^** |
| ***Sept Fish*** |  |  |  |
| complement C1q-like protein 4 precursor^c^ | A_05_P332842 | 2.994 | 7.70E-03 |
| trophoblast glycoprotein | A_05_P341612 | 2.915 | 1.67E-03 |
| protein bat5 | A_05_P421177 | 2.083 | 2.54E-02 |
| phytanoyl- hydroxylase-interacting | A_05_P421337 | 1.996 | 1.92E-02 |
| daz associated protein 2 | A_05_P493877 | 1.934 | 1.03E-02 |
| 39s ribosomal protein mitochondrial-like | A_05_P472317 | 1.901 | 1.53E-02 |
| hydroxymethylglutaryl- mitochondrial-like | A_05_P404207 | 1.859 | 4.69E-03 |
| zona pellucida glycoprotein | A_05_P456902 | 1.835 | 1.52E-02 |
| topoisomerase II-associated protein PAT1^c^ | A_05_P487287 | 1.815 | 2.14E-02 |
| fast myotomal muscle troponin-t-1 | A_05_P470352 | 1.779 | 2.91E-02 |
| methionine adenosyltransferase alpha^e^ | A_05_P425092 | 1.767 | 1.31E-02 |
| betaine--homocysteine s-methyltransferase 1 | A_05_P254689 | 1.715 | 7.04E-03 |
| senescence-associated protein^e^ | A_05_P393707 | 1.704 | 1.23E-02 |
| single-stranded dna binding protein 3 | A_05_P301527 | 1.695 | 1.18E-02 |
| zinc finger protein zic 3 | A_05_P331487 | 1.695 | 9.61E-03 |
| copper chaperone for superoxide dismutase | A_05_P285792 | 1.672 | 2.47 E-02 |
| sjchgc09650 protein | A_05_P417617 | 1.669 | 1.40E-02 |
| polycystic kidney disease protein 1-like 3 precursor^c^ | A_05_P299292 | 1.658 | 3.46E-02 |
| branched-chain-amino-acid cytosolic | A_05_P265754 | 1.639 | 1.45E-02 |
| golgi snap receptor complex member 2 | A_05_P341937 | 1.637 | 1.12E-02 |
| sarcosine mitochondrial | A_05_P266337 | 1.608 | 2.12E-02 |
| serine threonine-protein kinase 17a-like | A_05_P489187 | 1.608 | 4.54E-02 |
| enolase 3-2^e^ | A_05_P473007 | 1.592 | 8.47E-03 |
| septin-7 isoform 1 | A_05_P437742 | 1.582 | 2.50E-02 |
| myozenin-2 | A_05_P277187 | 1.560 | 2.02E-02 |
| novel protein | A_05_P269860 | 1.555 | 1.92E-02 |
| transforming growth beta- 68kda | A_05_P421617 | 1.546 | 3.07E-02 |
| helicase mov-10 | A_05_P255869 | 1.541 | 1.47E-02 |
| diacylglycerol o-acyltransferase 2 | A_05_P277787 | 1.538 | 4.45E-02 |
| hemoglobin subunit alpha^e^ | A_05_P249524 | 1.531 | 9.15E-03 |
| Legumain | A_05_P382032 | 1.531 | 4.32E-02 |
| hemoglobin subunit alpha | A_05_P441202 | 1.529 | 1.34E-02 |
| hemoglobin subunit beta-1 | A_05_P453042 | 1.527 | 7.97E-03 |
| class e basic helix-loop-helix protein 40 | A_05_P271931 | 1.522 | 2.35E-02 |
| trypsin domain containing 1^e^ | A_05_P468512 | 1.520 | 1.85E-02 |
| trna-splicing ligase homolog^e^ | A_05_P416447 | 1.513 | 3.01E-02 |
| spen transcriptional regulator^e^ | A_05_P333977 | 1.504 | 4.11E-02 |
| uncharacterized protein C21orf63 homolog precursor^c^ | A_05_P305517 | 1.502 | 2.68E-02 |
| protein FAM100A^c^ | A_05_P379837 | 1.486 | 3.45E-02 |
| RNA-binding protein 45 | A_05_P432127 | 1.484 | 4.25E-02 |
| sun domain-containing protein 1^e^ | A_05_P413772 | 1.477 | 2.61E-02 |
| pleckstrin homology domain-containing family g member 4b-like | A_05_P486407 | 1.466 | 2.02E-02 |
| ist1 homolog^e^ | A_05_P320332 | 1.458 | 1.08E-02 |
| hemoglobin subunit alpha^e^ | A_05_P449312 | 1.456 | 1.80E-02 |
| 5-aminolevulinate mitochondrial precursor | A_05_P275069 | 1.451 | 3.64E-02 |
| protein red | A_05_P310967 | 1.451 | 1.79E-02 |
| bruton agammaglobulinemia tyrosine kinase^e^ | A_05_P470042 | 1.451 | 1.24E-02 |
| toll-interacting protein | A_05_P467047 | 1.437 | 1.68E-02 |
| translation initiation factor if- mitochondrial-like | A_05_P300912 | 1.433 | 3.13E-02 |
| hemoglobin subunit alpha | A_05_P491417 | 1.433 | 2.63E-02 |
| flavin containing monooxygenase 5^e^ | A_05_P377187 | 1.431 | 4.22E-02 |
| methyltransferase-like protein 21a^e^ | A_05_P377727 | 1.431 | 2.11E-02 |
| Protein | A_05_P400232 | 1.429 | 1.54E-02 |
| glycerol-3-phosphate dehydrogenase | A_05_P422337 | 1.427 | 4.07E-02 |
| c-c motif chemokine 13 precursor | A_05_P476022 | 1.425 | 3.88E-02 |
| c-type lectin | A_05_P249684 | 1.418 | 2.84E-02 |
| very long-chain specific acyl- mitochondrial isoform 1 precursor | A_05_P439167 | 1.406 | 3.21E-02 |
| RNA-binding protein 4 | A_05_P331017 | 1.401 | 1.51E-02 |
| ubtf protein^e^ | A_05_P485787 | 1.397 | 2.67E-02 |
| gtp-binding nuclear protein ran | A_05_P370122 | 1.389 | 4.57E-02 |
| nuclear transcription factor gamma | A_05_P292702 | 1.374 | 3.51E-02 |
| protein fam49a-like | A_05_P397522 | 1.370 | 3.40E-02 |
| RNA-directed dna polymerase from mobile element jockey-like | A_05_P297012 | 1.368 | 2.54E-02 |
| jumonji domain-containing 3 | A_05_P255334 | 1.366 | 1.62E-02 |
| decorin precursor | A_05_P249764 | 1.361 | 3.16E-02 |
| cell division control protein 2 homolog | A_05_P311652 | 1.361 | 2.90E-02 |
| pumilio domain-containing protein kiaa0020-like^e^ | A_05_P253379 | 1.353 | 3.61E-02 |
| synaptophysin | A_05_P439457 | 1.353 | 2.08E-02 |
| s-adenosylhomocysteine hydrolase^e^ | A_05_P254399 | 1.346 | 4.86E-02 |
| proactivator polypeptide precursor | A_05_P421792 | 1.344 | 4.21E-02 |
| ubiquitin-conjugating enzyme e2 variant 1 | A_05_P406767 | 1.342 | 3.74E-02 |
| ankyrin repeat family a protein 2 | A_05_P329802 | 1.332 | 3.11E-02 |
| dual specificity protein phosphatase cdc14a | A_05_P483362 | 1.332 | 4.13E-02 |
| multidrug and toxin extrusion protein 1-like | A_05_P485307 | 1.332 | 4.29E-02 |
| s100 calcium binding protein v2-like | A_05_P272881 | 1.330 | 3.09E-02 |
| pyruvate kinase | A_05_P390772 | 1.325 | 3.41E-02 |
| ras-related and estrogen-regulated growth inhibitor | A_05_P343227 | 1.307 | 3.30E-02 |
| nicotinamide phosphoribosyltransferase | A_05_P355253 | 1.302 | 4.13E-02 |
| down syndrome cell adhesion molecule-like protein cg42256-like | A_05_P424367 | 1.282 | 3.99E-02 |
| apolipoprotein l3-like^e^ | A_05_P451048 | 1.267 | 3.34E-02 |
| ***Dec Fish*** |  |  |  |
| nattectin precursor | A_05_P490962 | 6.024 | 4.75E-02 |
| lipocalin precursor | A_05_P414837 | 3.817 | 4.60E-02^d^ |
| polycystic kidney disease protein 1-like 3 precursor | A_05_P488952 | 2.732 | 4.89E-02^d^ |
| zinc transporter ZIP3 | A_05_P372187 | 2.688 | 4.66E-02^d^ |

^a^Fold change is the average difference in expression as measured by the microarray

^b^ Measures the significance of the difference in expression between the small and large fish.

^c^ Sequence was unnamed by Blast2go but named by Agilent

**^d^** Guassian p-value < 0.05, t-test p-value > 0.05

^e^ Identified as a different gene by Agilent

Genes that are up-regulated in both large & small fish across seasons are highlighted in red

Genes with significant up-regulation in large fish (but different probe Ids) across seasons are highlighted in yellow
