# Supplementary material for: Differential gene expression in small and large rainbow trout derived from two seasonal spawning groups
Source: BMC Genomics. 2014 Jan 22;15:57. doi: 10.1186/1471-2164-15-57 (PMC3931318; doi:10.1186/1471-2164-15-57)
Supplement: Additional file 2: Table S2 — Genes up-regulated in the liver of small rainbow trout compared to large rainbow trout. [file 1471-2164-15-57-S2.docx]

| **Supplementary Table 2: Genes up-regulated in the liver of small rainbow trout compared to large rainbow trout** | | | |
| --- | --- | --- | --- |
| **Gene Name** | **Gene Number** | **Fold change^a^** | **p-value^b^** |
| ***Sept Fish*** |  |  |  |
| glutathione s-transferase p | A_05_P278137 | 4.709 | 7.55E-05 |
| terf1 -interacting nuclear factor 2 | A_05_P292537 | 3.673 | 3.30E-02 |
| thioredoxin | A_05_P465152 | 3.190 | 1.59E-02 |
| hemagglutinin/amebocyte aggregation factor precursor^c^ | A_05_P250874 | 3.127 | 2.51E-02 |
| beta-galactosyltransferase 1 | A_05_P430162 | 3.034 | 1.19E-03 |
| proteasome activator complex subunit 3 | A_05_P254864 | 2.843 | 4.13E-02 |
| lipocalin precursor | A_05_P414837 | 2.673 | 1.90E-03 |
| ras-related protein rab-10 | A_05_P415607 | 2.234 | 8.60E-05 |
| RNA-binding protein 28 isoform 2 | A_05_P488912 | 2.231 | 1.86E-02 |
| troponin I, slow skeletal muscle^c^ | A_05_P250684 | 2.212 | 1.88E-04 |
| thymosin beta-a | A_05_P261819 | 2.146 | 2.83E-04 |
| defensin beta 3 | A_05_P267859 | 2.079 | 3.37E-05 |
| leukotriene a-4 hydrolase^e^ | A_05_P375747 | 2.041 | 1.72E-04 |
| complement c1q tumor necrosis factor-related protein 3-like | A_05_P461537 | 1.963 | 4.57E-02 |
| gig2-like protein | A_05_P406932 | 1.936 | 3.95E-03 |
| glutathione peroxidase 3 precursor | A_05_P388822 | 1.936 | 9.19E-03 |
| isopentenyl-diphosphate delta-isomerase 1 | A_05_P443322 | 1.898 | 1.50E-02 |
| general transcription factor 3c polypeptide 6 | A_05_P436002 | 1.892 | 3.69E-05 |
| nuclease harbi1-like^e^ | A_05_P419577 | 1.870 | 4.08E-03 |
| lysyl oxidase | A_05_P439417 | 1.840 | 3.45E-02 |
| toll-like receptor 5 membrane form | A_05_P249089 | 1.836 | 2.57E-06 |
| lymphatic vessel endothelial hyaluronic acid receptor 1 precursor | A_05_P273604 | 1.831 | 2.40E-02 |
| protein transport protein sec61 subunit gamma | A_05_P471987 | 1.816 | 2.24E-02 |
| retinoblastoma binding protein 9^e^ | A_05_P316672 | 1.787 | 1.48E-03 |
| cold inducible rna binding protein | A_05_P395152 | 1.744 | 3.39E-02 |
| ornithine aminotransferase^e^ | A_05_P422847 | 1.729 | 2.07E-02 |
| myosin regulatory light chain 2, smooth muscle isoform^c^ | A_05_P425467 | 1.712 | 1.74E-02 |
| uridine phosphorylase 2 | A_05_P482647 | 1.689 | 2.84E-02 |
| transcription factor jun-b | A_05_P266894 | 1.656 | 5.95E-03 |
| deleted in malignant brain tumors 1 partial | A_05_P391642 | 1.620 | 1.98E-03 |
| insulin-like growth factor binding protein 1 | A_05_P489857 | 1.620 | 8.13E-03 |
| uncharacterized protein K02A2.6^c^ | A_05_P348202 | 1.620 | 3.78E-02 |
| 39s ribosomal protein mitochondrial-like | A_05_P298312 | 1.605 | 3.28E-03 |
| lathosterol oxidase-like | A_05_P251024 | 1.605 | 1.57E-02 |
| 7-dehydrocholesterol reductase | A_05_P472497 | 1.604 | 2.47E-03 |
| heme oxygenase | A_05_P413672 | 1.597 | 5.34E-03 |
| proteasome subunit alpha type-3 | A_05_P406597 | 1.595 | 2.86E-03 |
| androgen-dependent tpf1-regulating protein^e^ | A_05_P442202 | 1.594 | 4.20E-02 |
| transcription termination factor Rho; provisional^c^ | A_05_P399407 | 1.564 | 5.00E-02 |
| ADP-ribosylation factor-like protein 3 | A_05_P414547 | 1.557 | 2.99E-02 |
| lathosterol oxidase-like | A_05_P371977 | 1.554 | 1.42E-02 |
| growth arrest and DNA-damage-inducible protein gadd45 beta | A_05_P443342 | 1.548 | 1.37E-02 |
| orf2^e^ | A_05_P404212 | 1.544 | 1.08E-02 |
| isocitrate dehydrogenase | A_05_P265984 | 1.544 | 4.73E-02 |
| inactive rhomboid protein 1-like^e^ | A_05_P426507 | 1.533 | 7.79E-03 |
| tousled-like kinase | A_05_P374977 | 1.524 | 6.41E-03 |
| elongation of very long chain fatty acids protein 1 | A_05_P457892 | 1.520 | 3.67E-02 |
| 40S ribosomal protein S27^c^ | A_05_P475507 | 1.503 | 6.12E-03 |
| uncharacterized protein C7orf44^c^ | A_05_P414537 | 1.500 | 1.35E-03 |
| si:ch211-147a11.3^c^ | A_05_P477527 | 1.497 | 2.81E-02 |
| Ictacalcin | A_05_P488907 | 1.490 | 2.48E-02 |
| uncharacterized protein C2orf47 homolog,mitochondrial precursor^c^ | A_05_P418477 | 1.489 | 4.33E-02 |
| heme oxygenase | A_05_P275014 | 1.488 | 4.82E-02 |
| elongation of very long chain fatty acids protein 1 like | A_05_P321262 | 1.487 | 4.72E-03 |
| purine nucleoside phosphorylase | A_05_P415112 | 1.483 | 3.92E-02 |
| gamma-interferon-inducible lysosomal thiol reductase precursor^c^ | A_05_P270919 | 1.481 | 1.43E-02 |
| 7-dehydrocholesterol reductase | A_05_P377532 | 1.473 | 3.92E-02 |
| tripartite motif-containing 8 | A_05_P371862 | 1.456 | 4.92E-02 |
| rab11 family-interacting protein 2 | A_05_P385962 | 1.451 | 3.43E-04 |
| torsin family protein c9orf167 homolog | A_05_P293732 | 1.451 | 3.37E-02 |
| lysosomal protective protein precursor | A_05_P366227 | 1.445 | 3.70E-04 |
| sorting nexin-14 | A_05_P456942 | 1.445 | 4.06E-03 |
| cdgsh iron sulfur domain 1 | A_05_P301727 | 1.444 | 1.45E-02 |
| protein NLRC3^c^ | A_05_P479342 | 1.441 | 1.32E-03 |
| zinc finger protein dpf3 | A_05_P436152 | 1.430 | 2.42E-02 |
| uracil phosphoribosyltransferase homolog | A_05_P308352 | 1.424 | 3.37E-02 |
| stress-associated endoplasmic reticulum protein 1 | A_05_P368117 | 1.421 | 2.28E-02 |
| dna damage-binding protein 1 | A_05_P453112 | 1.420 | 8.66E-03 |
| si:rp71-1g18.9^c^ | A_05_P388852 | 1.419 | 3.44E-02 |
| dead (asp-glu-ala-asp) box polypeptide 47 | A_05_P458287 | 1.418 | 5.93E-03 |
| transmembrane protein 180 | A_05_P308337 | 1.418 | 4.61E-02 |
| protein phosphatase 1 regulatory subunit 3d | A_05_P352332 | 1.414 | 5.31E-04 |
| cyclin-dependent kinase 7 | A_05_P429322 | 1.409 | 2.04E-02 |
| cerebellin-1 precursor^e^ | A_05_P256104 | 1.408 | 3.99E-02 |
| sestrin 1 | A_05_P273169 | 1.407 | 1.77E-03 |
| proto-oncogene serine threonine-protein kinase pim-1 | A_05_P271659 | 1.403 | 6.70E-03 |
| ribosome biogenesis protein bms1 homolog | A_05_P424637 | 1.399 | 4.13E-02 |
| DNA-binding protein inhibitor id-2 | A_05_P364502 | 1.398 | 8.84E-03 |
| nucleoporin nup37 | A_05_P274404 | 1.397 | 1.01E-03 |
| ADP-ribosylation factor-like protein 4d | A_05_P429187 | 1.396 | 2.93E-02 |
| mps one binder kinase activator-like 1b | A_05_P410142 | 1.396 | 9.23E-03 |
| caldesmon^c^ | A_05_P345307 | 1.392 | 7.15E-04 |
| ADP-ribosylation factor-like protein 3 | A_05_P265169 | 1.386 | 3.91E-02 |
| synaptotagmin cytoplasmic rna interacting protein^e^ | A_05_P302797 | 1.385 | 5.24E-03 |
| transmembrane protein 147 | A_05_P408342 | 1.385 | 1.53E-02 |
| solute carrier family 35 member b1 | A_05_P448242 | 1.384 | 4.90E-02 |
| RNA-directed DNA polymerase from mobile element jockey^c^ | A_05_P314887 | 1.378 | 4.50E-03 |
| ras-related protein rab-37-like | A_05_P418632 | 1.378 | 4.06E-02 |
| B-cell receptor CD22 precursor^c^ | A_05_P267034 | 1.377 | 5.55E-03 |
| gpi mannosyltransferase 1 | A_05_P330447 | 1.368 | 2.18E-03 |
| protein strawberry notch homolog 1 | A_05_P275549 | 1.365 | 1.51E-03 |
| zinc finger protein partial | A_05_P281607 | 1.365 | 4.55E-03 |
| sterol-c4-methyl oxidase-like | A_05_P371933 | 1.364 | 1.70E-02 |
| spermine synthase | A_05_P378927 | 1.361 | 2.04E-02 |
| acyl- dehydrogenase family member 10^e^ | A_05_P430707 | 1.359 | 2.32E-02 |
| member ras oncogene family | A_05_P275884 | 1.359 | 5.72E-03 |
| non-lysosomal glucosylceramidase | A_05_P373217 | 1.358 | 1.58E-03 |
| proteasome ( macropain) 26s 6 | A_05_P430207 | 1.358 | 2.89E-02 |
| 6-phosphofructo-2-kinase fructose- -biphosphatase 2 | A_05_P296412 | 1.357 | 3.91E-03 |
| g-protein coupled receptor 4 | A_05_P475167 | 1.356 | 2.93E-02 |
| guanine nucleotide binding protein (g protein) alpha inhibiting activity polypeptide 2 | A_05_P457457 | 1.356 | 3.73E-02 |
| zinc finger protein 502-like^e^ | A_05_P403505 | 1.355 | 1.08E-02 |
| transcription factor jun-b | A_05_P410847 | 1.352 | 5.48E-03 |
| kinesin-associated protein 3 | A_05_P333172 | 1.351 | 2.00E-02 |
| protein las1 homolog | A_05_P488567 | 1.349 | 4.76E-02 |
| protein FAM111A^c^ | A_05_P300512 | 1.348 | 9.16E-03 |
| class member 1^e^ | A_05_P341377 | 1.347 | 1.57E-02 |
| nuclear receptor subfamily 0 group b member 2 | A_05_P371227 | 1.346 | 4.47E-02 |
| carbohydrate sulfotransferase 15^e^ | A_05_P332757 | 1.345 | 5.46E-03 |
| dihydroorotate dehydrogenase | A_05_P397537 | 1.341 | 1.13E-02 |
| sfrs12ip1 protein | A_05_P351412 | 1.337 | 6.00E-03 |
| uncharacterized protein C14orf28 homolog^c^ | A_05_P435532 | 1.334 | 1.70E-02 |
| angiopoietin-related protein 4 precursor | A_05_P450957 | 1.334 | 2.99E-02 |
| cytochrome c | A_05_P365597 | 1.332 | 4.80E-02 |
| neutrophil cytosolic factor 4 | A_05_P258719 | 1.331 | 2.26E-02 |
| copper transport protein atox1 | A_05_P364682 | 1.331 | 2.66E-02 |
| tyrosyl-trna synthetase | A_05_P327312 | 1.330 | 1.34E-02 |
| growth arrest and dna-damage- beta | A_05_P409552 | 1.329 | 5.12E-03 |
| origin recognition complex subunit 4 | A_05_P258059 | 1.329 | 4.32E-03 |
| voltage-dependent anion-selective channel protein 1 | A_05_P409492 | 1.329 | 4.58E-02 |
| NADH dehydrogenase subunit 4l | A_05_P460457 | 1.327 | 4.86E-02 |
| glyoxalase domain-containing protein 5^c^ | A_05_P288622 | 1.324 | 2.06E-02 |
| transducer of 1^e^ | A_05_P275989 | 1.320 | 3.44E-03 |
| serine threonine-protein kinase 17a | A_05_P375877 | 1.320 | 4.43E-02 |
| zinc finger protein partial | A_05_P483017 | 1.318 | 4.85E-02 |
| cd9 antigen | A_05_P448732 | 1.318 | 1.73E-02 |
| cyclin-dependent kinase 2-associated protein 1 | A_05_P461307 | 1.318 | 4.23E-02 |
| nuclear factor nf-kappa-b p105 subunit | A_05_P254299 | 1.317 | 4.24E-02 |
| mannoside acetylglucosaminyltransferase isoform cra_a | A_05_P393322 | 1.316 | 4.62E-02 |
| signal transducer and activator of transcription 3 | A_05_P390597 | 1.315 | 4.26E-02 |
| upf0551 protein c8orf38 mitochondrial-like^e^ | A_05_P407372 | 1.314 | 3.89E-03 |
| prothymosin, prothymosin/parathymosin family^c^ | A_05_P275074 | 1.314 | 4.37E-02 |
| egf-like module-containing mucin-like hormone receptor-like 1-like | A_05_P365887 | 1.313 | 4.23E-02 |
| transmembrane protein 114 | A_05_P329542 | 1.313 | 4.07E-02 |
| lamina-associated polypeptide isoforms beta gamma-like | A_05_P275319 | 1.312 | 2.09E-02 |
| NADH dehydrogenase | A_05_P397872 | 1.311 | 1.52E-02 |
| mucin, mucin-like glycoprotein^c^ | A_05_P463472 | 1.311 | 4.69E-02 |
| biorientation of chromosomes in cell division protein 1^e^ | A_05_P439197 | 1.310 | 9.86E-03 |
| ras-related protein rab-27b | A_05_P348947 | 1.308 | 4.72E-02 |
| 2-aminoethanethiol dioxygenase | A_05_P398067 | 1.308 | 3.46E-02 |
| coiled-coil-helix-coiled-coil-helix domain-containingn protein 6^c^ | A_05_P439372 | 1.308 | 2.75E-02 |
| f11 receptor^e^ | A_05_P420202 | 1.308 | 1.87E-02 |
| myosin ic | A_05_P492767 | 1.308 | 3.38E-02 |
| hydroxysteroid (17-beta) dehydrogenase 8 | A_05_P265514 | 1.305 | 2.83E-02 |
| spermatogenesis-associated protein 2 | A_05_P462937 | 1.304 | 3.36E-02 |
| rho gtpase-activating protein 1 | A_05_P439712 | 1.303 | 3.21E-02 |
| kruppel-like factor 2 | A_05_P409818 | 1.302 | 2.29E-02 |
| vesicle-trafficking protein sec22b-like | A_05_P400162 | 1.299 | 3.02E-02 |
| kinesin-like protein kif2a | A_05_P323012 | 1.298 | 1.17E-02 |
| sterol-c4-methyl oxidase-like | A_05_P477862 | 1.298 | 4.15E-02 |
| spindlin 1 | A_05_P268134 | 1.297 | 2.82E-02 |
| riken cdna 2810453i06 gene | A_05_P410217 | 1.296 | 3.90E-02 |
| acyl-protein thioesterase 2 | A_05_P410252 | 1.295 | 4.44E-02 |
| t-complex protein 1 subunit beta | A_05_P418142 | 1.294 | 3.39E-02 |
| transmembrane protein 198-b-like | A_05_P329512 | 1.294 | 4.81E-02^d^ |
| cap-gly domain-containing linker protein 1 | A_05_P257189 | 1.290 | 4.68E-02^d^ |
| glutamine synthetase | A_05_P429307 | 1.289 | 2.06E-02 |
| squalene monooxygenase | A_05_P376672 | 1.286 | 4.15E-02 |
| ATP-binding cassette sub-family e member 1 | A_05_P444272 | 1.286 | 4.17E-02 |
| ADP-ribosylation factor 1 | A_05_P366457 | 1.281 | 1.34E-02 |
| olfactomedin 2^e^ | A_05_P255884 | 1.281 | 7.40E-03 |
| epigen precursor | A_05_P355932 | 1.278 | 3.52E-02 |
| papilin precursor^c^ | A_05_P433482 | 1.277 | 2.79E-02 |
| s-adenosylmethionine synthase isoform type-2 | A_05_P425317 | 1.277 | 3.34E-02 |
| yrdc domain-containing mitochondrial-like^e^ | A_05_P333832 | 1.277 | 4.87E-02 |
| protein arginine n-methyltransferase 5 | A_05_P417412 | 1.276 | 1.77E-02 |
| ribosomal protein l13a | A_05_P394232 | 1.275 | 4.69E-02^d^ |
| zinc finger protein 2 homolog | A_05_P386640 | 1.274 | 4.59E-02 |
| wd repeat-containing protein 26 | A_05_P271304 | 1.274 | 2.15E-02 |
| nucleolar protein 16-like^e^ | A_05_P412172 | 1.272 | 4.49E-02 |
| transmembrane 9 superfamily member 2 | A_05_P386262 | 1.271 | 3.14E-02 |
| pancreas duodenum homeobox protein 1 | A_05_P338287 | 1.269 | 3.43E-02 |
| UDP-n-acetylhexosamine pyrophosphorylase-like protein 1 | A_05_P304822 | 1.269 | 4.75E-02 |
| phd finger-like domain-containing protein 5a | A_05_P335447 | 1.267 | 2.03E-02 |
| jumping translocation breakpoint | A_05_P300882 | 1.266 | 3.89E-02 |
| myosin if^e^ | A_05_P305457 | 1.266 | 3.69E-02 |
| molybdenum cofactor synthesis 1 | A_05_P303482 | 1.264 | 3.48E-02 |
| phosphatidylinositol glycan anchor class u | A_05_P346472 | 1.261 | 2.62E-02 |
| zinc finger protein 658b-like^e^ | A_05_P354037 | 1.260 | 8.13E-03 |
| zinc finger protein 568 isoform partial | A_05_P282062 | 1.260 | 4.99E-02^d^ |
| protein prune homolog 2^e^ | A_05_P406537 | 1.260 | 4.02E-02 |
| myosin-11 isoform 2 | A_05_P369302 | 1.259 | 1.72E-02 |
| high mobility group protein b3 | A_05_P312192 | 1.258 | 2.21E-02 |
| pantothenate kinase 1 | A_05_P429077 | 1.258 | 2.92E-02 |
| ring finger and spry domain containing 1 | A_05_P459662 | 1.258 | 3.13E-02 |
| nedd8-activating enzyme e1 catalytic subunit | A_05_P318407 | 1.257 | 4.80E-02 |
| histone deacetylase complex subunit sap30l | A_05_P300927 | 1.256 | 2.58E-02 |
| dynactin subunit 6 | A_05_P309537 | 1.254 | 2.82E-02 |
| unc93-like protein mfsd11 isoform 1 | A_05_P310947 | 1.254 | 3.21E-02 |
| serine threonine-protein kinase plk2 | A_05_P291852 | 1.253 | 2.15E-02 |
| calcineurin-binding protein cabin-1 | A_05_P445417 | 1.253 | 4.17E-02 |
| pre-mRNA-splicing factor rbm22 | A_05_P316597 | 1.252 | 4.68E-02 |
| tissue factor pathway inhibitor-like | A_05_P416057 | 1.252 | 4.79E-02 |
| protein fam134c-like | A_05_P389097 | 1.250 | 1.19E-02 |
| smc4 protein | A_05_P337517 | 1.248 | 4.35E-02 |
| brain protein 44-like protein^c^ | A_05_P425832 | 1.247 | 4.38E-02 |
| ankyrin repeat domain-containing protein 1^e^ | A_05_P344042 | 1.246 | 1.57E-02 |
| n-glycanase 1^e^ | A_05_P295512 | 1.245 | 3.75E-02 |
| group xiia secretory phospholipase a2 precursor | A_05_P253794 | 1.241 | 3.48E-02 |
| e3 ubiquitin-protein ligase rnf8 | A_05_P316697 | 1.240 | 1.09E-02 |
| serine threonine-protein phosphatase 6 regulatory ankyrin repeat subunit b^e^ | A_05_P486502 | 1.240 | 1.50E-02 |
| retinol dehydrogenase 12 | A_05_P487192 | 1.239 | 3.89E-02 |
| complement c1q tumor necrosis factor-related protein 5-like | A_05_P428642 | 1.237 | 3.94E-02 |
| lymphocyte-specific protein tyrosine kinase | A_05_P322837 | 1.236 | 2.36E-02 |
| immunoglobulin V-set domain^c^ | A_05_P333317 | 1.236 | 4.35E-02 |
| zinc finger protein partial | A_05_P487787 | 1.235 | 2.01E-02 |
| novel protein human titin | A_05_P448672 | 1.235 | 2.10E-02 |
| krab box and zinc finger c2h2 type domain containing protein^e^ | A_05_P307152 | 1.233 | 4.99E-02 |
| cytochrome p450 2u1 | A_05_P488342 | 1.233 | 3.17E-02 |
| coiled-coil domain-containing protein 43^c^ | A_05_P425624 | 1.229 | 4.84E-02 |
| allograft inflammatory factor 1 | A_05_P383672 | 1.228 | 2.50E-02 |
| transport-associated protein^e^ | A_05_P417477 | 1.227 | 4.47E-02^d^ |
| arf-gap with coiled- ank repeat and ph domain-containing protein 2-like | A_05_P431317 | 1.226 | 3.51E-02 |
| RNA-binding motif x-linked 2 | A_05_P473782 | 1.226 | 4.88E-02^d^ |
| alpha- -mannosyl-glycoprotein 4-beta-n-acetylglucosaminyltransferase b | A_05_P404917 | 1.225 | 3.82E-02 |
| serum albumin precursor | A_05_P249414 | 1.225 | 4.02E-02 |
| protein wntless homolog isoform 1^e^ | A_05_P375912 | 1.225 | 3.67E-02 |
| set and mynd domain-containing protein 3 | A_05_P487912 | 1.221 | 1.46E-02 |
| acidic leucine-rich nuclear phosphoprotein 32 family member b | A_05_P326487 | 1.219 | 3.98E-02 |
| methionine aminopeptidase 1 | A_05_P459832 | 1.218 | 2.74E-02 |
| nad-dependent epimerase dehydratase^e^ | A_05_P355107 | 1.215 | 4.75E-02 |
| g-protein coupled receptor 183^e^ | A_05_P320972 | 1.211 | 3.74E-02 |
| transmembrane protein 30a^e^ | A_05_P261264 | 1.211 | 4.76E-02 |
| cell division cycle 42 (gtp binding 25kda) | A_05_P394482 | 1.210 | 5.00E-02^d^ |
| expressed sequence aw146154^e^ | A_05_P344917 | 1.209 | 4.75E-02 |
| guanine nucleotide-binding protein g g g subunit gamma-t2 | A_05_P390452 | 1.207 | 2.28E-02 |
| synaptosomal-associated 91kda homolog^e^ | A_05_P304607 | 1.206 | 2.39E-02 |
| glutamyl-trna amidotransferase subunit a homolog | A_05_P440712 | 1.206 | 4.47E-02^d^ |
| ***Dec Fish*** |  |  |  |
| insulin-like growth factor binding protein 1 | A_05_P489857 | 3.274 | 2.88E-04 |
| delta (14)-sterol reductase | A_05_P304042 | 2.787 | 2.66E-02 |
| e3 ubiquitin-protein ligase neurl3-like | A_05_P444687 | 2.490 | 3.55E-03 |
| hemoglobin subunit beta | A_05_P488772 | 2.162 | 3.40E-02 |
| mucolipin 3 | A_05_P473797 | 2.113 | 9.12E-03 |
| aconitate mitochondrial-like | A_05_P379862 | 2.112 | 3.92E-03 |
| t-cell immunoglobulin and mucin domain containing protein 4 precursor | A_05_P369897 | 1.986 | 2.56E-02 |
| leucine-rich repeat-containing protein 31^c^ | A_05_P446667 | 1.966 | 4.21E-03 |
| squalene monooxygenase | A_05_P424717 | 1.932 | 3.24E-02 |
| snurportin 1 | A_05_P285382 | 1.918 | 4.18E-02 |
| glycerate kinase-like | A_05_P301037 | 1.881 | 1.93E-02 |
| enolase 3-2^e^ | A_05_P466287 | 1.880 | 1.38E-02 |
| apoptosis regulator bax | A_05_P261324 | 1.862 | 3.54E-03 |
| g-protein-signaling modulator 2 | A_05_P471202 | 1.860 | 3.42E-02 |
| zinc finger protein 182 | A_05_P399542 | 1.857 | 2.88E-03 |
| malate dehydrogenase 1b | A_05_P294817 | 1.834 | 4.22E-02 |
| hemoglobin subunit alpha^e^ | A_05_P480657 | 1.827 | 8.94E-03 |
| ras-related protein rab-37-like | A_05_P304637 | 1.810 | 4.52E-02 |
| protein transport protein sec61 subunit gamma | A_05_P471987 | 1.782 | 2.98E-02 |
| tetratricopeptide repeat protein 23 | A_05_P450727 | 1.776 | 3.63E-03 |
| vascular endothelial growth factor c | A_05_P452257 | 1.740 | 2.84E-02 |
| farnesyl pyrophosphate synthetase | A_05_P266579 | 1.740 | 3.07E-02 |
| senescence-associated protein | A_05_P262709 | 1.732 | 4.20E-02 |
| family with sequence similarity 19 (chemokine (c-c motif)-like) member a5 | A_05_P449432 | 1.719 | 3.12E-02 |
| hemoglobin subunit alpha | A_05_P491417 | 1.716 | 3.59E-03 |
| 39s ribosomal protein mitochondrial precursor | A_05_P334607 | 1.698 | 3.52E-02 |
| senescence-associated protein^e^ | A_05_P476718 | 1.692 | 2.00E-02 |
| protein fam49a-like | A_05_P397522 | 1.689 | 4.20E-03 |
| cytochrome c oxidase subunit vic | A_05_P276699 | 1.687 | 2.21E-02 |
| glutamine synthetase | A_05_P429307 | 1.685 | 3.71E-02 |
| monoglyceride lipase | A_05_P392532 | 1.684 | 6.73E-03 |
| phosducin-like protein 3 | A_05_P430992 | 1.674 | 9.56E-03 |
| ES1 protein homolog, mitochondrial precursor^c^ | A_05_P425787 | 1.670 | 3.85E-02 |
| hemoglobin subunit alpha^e^ | A_05_P449312 | 1.660 | 1.15E-02 |
| peroxisome proliferator-activated receptor gamma | A_05_P248874 | 1.656 | 3.67E-02 |
| cyclin-dependent kinase inhibitor 3 | A_05_P385997 | 1.646 | 2.07E-02 |
| hemoglobin subunit beta^c^ | A_05_P463622 | 1.636 | 2.16E-02 |
| hemoglobin subunit beta-1 | A_05_P453042 | 1.632 | 4.58E-03 |
| phosphoribosylaminoimidazole phosphoribosylaminoimidazole succinocarboxamide synthetase^e^ | A_05_P368142 | 1.627 | 4.51E-02 |
| phosphoglycerate mutase 1 | A_05_P252889 | 1.625 | 2.01E-02 |
| calcipressin-2-like isoform 2 | A_05_P288242 | 1.615 | 4.39E-02^d^ |
| zinc finger protein 341 | A_05_P339332 | 1.604 | 4.15E-02 |
| claudin-4 | A_05_P274739 | 1.599 | 1.19E-02 |
| hemoglobin subunit alpha^e^ | A_05_P249524 | 1.583 | 1.75E-02 |
| lactate dehydrogenase b | A_05_P274809 | 1.574 | 1.52E-02 |
| cartilage intermediate layer nucleotide pyrophosphohydrolase | A_05_P355452 | 1.569 | 1.10E-02 |
| RNA binding protein with multiple splicing 2 | A_05_P255199 | 1.561 | 4.68E-02 |
| protein arginine n-methyltransferase 7-like | A_05_P375442 | 1.560 | 4.33E-02 |
| sjchgc07628 protein | A_05_P343637 | 1.555 | 4.61E-02 |
| talin-1 | A_05_P301887 | 1.552 | 1.25E-02 |
| zinc finger protein 568 isoform partial | A_05_P282062 | 1.546 | 2.90E-02 |
| ribosomal protein s13 | A_05_P471392 | 1.544 | 1.20E-02 |
| cell division control protein 2 homolog | A_05_P311652 | 1.535 | 4.20E-02 |
| glutamine synthetase | A_05_P330397 | 1.527 | 1.82E-02 |
| Ig kappa chain V-III region IARC/BL41 precursor^c^ | A_05_P453132 | 1.526 | 2.97E-02 |
| laminin subunit beta-2 | A_05_P397982 | 1.525 | 1.86E-02 |
| DNA-binding protein inhibitor id-1 | A_05_P273294 | 1.521 | 1.60E-02 |
| tetranectin precursor | A_05_P389242 | 1.515 | 4.02E-02 |
| cytohesin 1 | A_05_P311307 | 1.511 | 3.84E-02 |
| Nucleolin | A_05_P265374 | 1.511 | 4.07E-02^d^ |
| serine threonine-protein kinase ick | A_05_P374562 | 1.510 | 3.94E-02 |
| novel protein vertebrate udp-glycosyltransferase family^e^ | A_05_P255934 | 1.505 | 1.22E-02 |
| methyltransferase-like protein 4-like | A_05_P459427 | 1.503 | 3.19E-02 |
| microfibril-associated glycoprotein 4 precursor^c^ | A_05_P319957 | 1.502 | 2.40E-02 |
| beta-ureidopropionase | A_05_P326612 | 1.492 | 2.42E-02 |
| novel protein human titin | A_05_P448672 | 1.492 | 4.74E-02 |
| band 3 anion exchange protein | A_05_P269349 | 1.486 | 4.19E-02 |
| glutamine synthetase | A_05_P388642 | 1.475 | 3.22E-02 |
| brefeldin a-inhibited guanine nucleotide-exchange protein 1 | A_05_P330597 | 1.472 | 4.22E-02 |
| apoptosis-inducing factor 3-like | A_05_P400182 | 1.467 | 4.22E-02^d^ |
| integrin alpha-x | A_05_P326252 | 1.464 | 2.33E-02 |
| 7-dehydrocholesterol reductase | A_05_P259754 | 1.463 | 4.36E-02 |
| DNA repair protein complementing xp-a cells | A_05_P450437 | 1.461 | 3.96E-02 |
| sestrin 1 | A_05_P439562 | 1.450 | 2.34E-02 |
| integrase core domain^c^ | A_05_P347262 | 1.449 | 3.90E-02 |
| uncharacterized protein C10orf27^c^ | A_05_P456702 | 1.444 | 4.87E-02^d^ |
| ox-2 membrane glycol | A_05_P442772 | 1.443 | 3.91E-02 |
| protein hexim1-like | A_05_P435547 | 1.442 | 4.97E-02 |
| protein Ag2 homolog^c^ | A_05_P375127 | 1.436 | 4.96E-02^d^ |
| mitochondrial ribosomal protein s16^e^ | A_05_P443957 | 1.435 | 3.14E-02 |
| transitional endoplasmic reticulum atpase | A_05_P400542 | 1.434 | 4.74E-02 |
| activating signal cointegrator 1 complex subunit 2^c^ | A_05_P297227 | 1.420 | 3.54E-02 |
| xpo6 partial | A_05_P267629 | 1.420 | 2.65E-02 |
| acetyl- cytosolic | A_05_P417942 | 1.419 | 3.78E-02 |
| probable g-protein coupled receptor 132 | A_05_P414137 | 1.417 | 3.43E-02 |
| a kinase anchor protein 7 | A_05_P432387 | 1.415 | 3.77E-02 |
| protein fam134a-like | A_05_P346307 | 1.414 | 4.71E-02 |
| zinc finger protein 22^e^ | A_05_P395942 | 1.413 | 3.17E-02 |
| bruton agammaglobulinemia tyrosine kinase^e^ | A_05_P481757 | 1.412 | 4.30E-02 |
| insulin-like growth factor binding protein 1 | A_05_P249019 | 1.399 | 3.27E-02 |
| trophoblast glycoprotein | A_05_P486127 | 1.389 | 4.61E-02 |
| selenoprotein w | A_05_P253264 | 1.387 | 4.60E-02 |
| cd9 antigen^e^ | A_05_P294587 | 1.384 | 4.60E-02 |
| ATP synthase subunit mitochondrial | A_05_P466172 | 1.383 | 4.74E-02 |
| NAD-dependent ADP-ribosyltransferase sirtuin-4-like | A_05_P471972 | 1.379 | 4.51E-02 |
| probable carboxypeptidase pm20d1 precursor | A_05_P371434 | 1.371 | 4.96E-02^d^ |
| ectonucleoside triphosphate diphosphohydrolase 8 | A_05_P272139 | 1.362 | 4.64E-02 |
| ubiquitin-conjugating enzyme e2 g2 | A_05_P289367 | 1.353 | 4.52E-02^d^ |

^a^Fold change is the average difference in expression as measured by the microarray

^b^ Measures the significance of the difference in expression between the small and large fish.

^c^ Sequence was unnamed by Blast2go but named by Agilent

**^d^** Guassian p-value < 0.05, t-test p-value > 0.05

^e^ Identified as a different gene by Agilent

Genes with significant up-regulation in small fish across seasons are highlighted in green

Genes that are up-regulated in both large & small fish across seasons are highlighted in red
