# Supplementary material for: Differential gene expression in small and large rainbow trout derived from two seasonal spawning groups
Source: BMC Genomics. 2014 Jan 22;15:57. doi: 10.1186/1471-2164-15-57 (PMC3931318; doi:10.1186/1471-2164-15-57)
Supplement: Additional file 3: Table S3 — Genes of unknown function up-regulated in the liver of large rainbow trout compared to small rainbow trout. [file 1471-2164-15-57-S3.docx]

| **Supplementary Table 3: Genes of unknown function up-regulated in the liver of large rainbow trout compared to small rainbow trout** | | | | | |
| --- | --- | --- | --- | --- | --- |
| **Gene Number** | **Fold change^a^** | **P value^b^** | **Gene Number** | **Fold change^a^** | **P value^b^** |
| ***Sept Fish*** |  |  | ***Sept Fish*** |  |  |
| A_05_P329377 | 3.279 | 1.84E-02 | A_05_P462867 | 2.688 | 6.02E-03 |
| A_05_P493007 | 2.646 | 3.58E-02 | A_05_P316022 | 2.545 | 1.63E-02 |
| A_05_P422962 | 2.427 | 1.28E-02 | A_05_P492002 | 2.364 | 2.47E-02 |
| A_05_P357857 | 2.101 | 5.33E-03 | A_05_P308297 | 2.066 | 8.38E-03 |
| A_05_P250624 | 1.938 | 1.95E-02 | A_05_P259164 | 1.919 | 9.56E-03 |
| A_05_P317717 | 1.916 | 7.52E-03 | A_05_P285137 | 1.887 | 7.41E-03 |
| A_05_P250629 | 1.883 | 2.58E-02 | A_05_P427432 | 1.818 | 8.30E-03 |
| A_05_P390222 | 1.727 | 2.63E-02 | A_05_P301967 | 1.721 | 7.51E-03 |
| A_05_P412947 | 1.709 | 3.37E-02 | A_05_P490392 | 1.701 | 4.74E-02 |
| A_05_P447492 | 1.692 | 9.99E-03 | A_05_P313972 | 1.658 | 2.35E-02 |
| A_05_P478587 | 1.650 | 3.21E-02 | A_05_P267154 | 1.634 | 1.03E-02 |
| A_05_P456972 | 1.631 | 1.58E-02 | A_05_P288237 | 1.623 | 2.16E-02 |
| A_05_P405117 | 1.621 | 4.43E-02 | A_05_P266724 | 1.618 | 4.40E-02 |
| A_05_P393862 | 1.616 | 1.73E-02 | A_05_P386002 | 1.610 | 2.01E-02 |
| A_05_P311282 | 1.603 | 3.60E-02 | A_05_P326927 | 1.592 | 4.15E-02 |
| A_05_P402837 | 1.585 | 3.67E-02 | A_05_P383407 | 1.565 | 2.84E-02 |
| A_05_P343127 | 1.560 | 3.53E-02 | A_05_P336577 | 1.558 | 2.23E-02 |
| A_05_P484422 | 1.541 | 3.89E-02 | A_05_P316047 | 1.504 | 1.50E-02 |
| A_05_P359287 | 1.504 | 1.93E-02 | A_05_P469522 | 1.493 | 1.54E-02 |
| A_05_P357097 | 1.490 | 1.91E-02 | A_05_P353447 | 1.488 | 3.46E-02 |
| A_05_P341327 | 1.456 | 2.84E-02 | A_05_P325228 | 1.445 | 2.67E-02 |
| A_05_P463662 | 1.439 | 1.63E-02 | A_05_P286252 | 1.439 | 1.82E-02 |
| A_05_P316217 | 1.433 | 2.56E-02 | A_05_P463522 | 1.427 | 2.76E-02 |
| A_05_P311357 | 1.422 | 3.35E-02 | A_05_P422072 | 1.406 | 2.14E-02 |
| A_05_P352158 | 1.387 | 2.35E-02 | A_05_P299007 | 1.379 | 2.82E-02 |
| A_05_P275009 | 1.376 | 4.74E-02 | A_05_P314357 | 1.376 | 2.09E-02 |
| A_05_P411657 | 1.366 | 3.24E-02 | A_05_P478417 | 1.364 | 3.76E-02 |
| A_05_P267169 | 1.364 | 4.60E-02 | A_05_P295682 | 1.351 | 4.79E-02 |
| A_05_P310532 | 1.350 | 2.79E-02 | A_05_P338922 | 1.346 | 3.21E-02 |
| A_05_P327612 | 1.340 | 3.25E-02 | A_05_P419637 | 1.333 | 3.69E-02 |
| A_05_P281722 | 1.332 | 2.74E-02 | A_05_P278762 | 1.325 | 3.60E-02 |
| A_05_P461777 | 1.316 | 3.62E-02 | A_05_P257894 | 1.297 | 4.97E-02 |
| A_05_P454432 | 1.292 | 4.26E-02 | A_05_P317597 | 1.287 | 4.59E-02 |
| A_05_P362922 | 1.274 | 4.65E-02 | A_05_P288917 | 1.271 | 4.18E-02 |
| A_05_P412697 | 1.269 | 3.47E-02 | A_05_P264809 | 1.252 | 4.45E-02 |
| ***Dec Fish*** |  |  | ***Dec Fish*** |  |  |
| A_05_P481067 | 2.778 | 0.91E-02 |  |  |  |

^a^Fold change is the average difference in expression as measured by the microarray

^b^ Measures the significance of the difference in expression between the small and large fish with a t-test
