# Supplementary material for: Differential gene expression in small and large rainbow trout derived from two seasonal spawning groups
Source: BMC Genomics. 2014 Jan 22;15:57. doi: 10.1186/1471-2164-15-57 (PMC3931318; doi:10.1186/1471-2164-15-57)
Supplement: Additional file 4: Table S4 — Genes of unknown function up-regulated in the liver of small rainbow trout compared to large rainbow trout. [file 1471-2164-15-57-S4.docx]

| **Supplementary Table 4: Genes of unknown function up-regulated in the liver of small rainbow trout compared to large rainbow trout** | | | | | |
| --- | --- | --- | --- | --- | --- |
| **Gene Number** | **Fold change^a^** | **P value^b^** | **Gene Number** | **Fold change^a^** | **P value^b^** |
| ***Sept Fish*** |  |  | ***Sept Fish*** |  |  |
| A_05_P340332 | 3.234 | 3.31E-02 | A_05_P280122 | 2.783 | 1.65E-02 |
| A_05_P353557 | 2.680 | 4.66E-02 | A_05_P335672 | 2.477 | 1.81E-02 |
| A_05_P299612 | 2.192 | 7.92E-05 | A_05_P326117 | 2.169 | 2.72E-03 |
| A_05_P324942 | 2.164 | 9.39E-03 | A_05_P314332 | 2.020 | 2.11E-03 |
| A_05_P414267 | 1.980 | 2.66E-02 | A_05_P362352 | 1.955 | 2.01E-03 |
| A_05_P347862 | 1.951 | 6.72E-03 | A_05_P445857 | 1.941 | 2.55E-03 |
| A_05_P350857 | 1.936 | 1.13E-04 | A_05_P483507 | 1.896 | 1.86E-03 |
| A_05_P423122 | 1.888 | 1.13E-05 | A_05_P454612 | 1.863 | 5.24E-03 |
| A_05_P293227 | 1.851 | 1.54E-02 | A_05_P264229 | 1.850 | 1.37E-02 |
| A_05_P264794 | 1.847 | 8.76E-04 | A_05_P416642 | 1.790 | 2.47E-02 |
| A_05_P337122 | 1.794 | 2.02E-03 | A_05_P477552 | 1.767 | 3.73E-03 |
| A_05_P326237 | 1.729 | 3.16E-03 | A_05_P486327 | 1.720 | 4.73E-02 |
| A_05_P463222 | 1.696 | 1.85E-04 | A_05_P462992 | 1.695 | 3.19E-02 |
| A_05_P276499 | 1.673 | 5.64E-03 | A_05_P336107 | 1.672 | 4.21E-03 |
| A_05_P421692 | 1.661 | 7.94E-05 | A_05_P290582 | 1.655 | 1.49E-03 |
| A_05_P353858 | 1.649 | 7.49E-04 | A_05_P338282 | 1.647 | 1.57E-02 |
| A_05_P307492 | 1.620 | 1.05E-02 | A_05_P488899 | 1.614 | 1.35E-02 |
| A_05_P384407 | 1.600 | 1.28E-03 | A_05_P433987 | 1.595 | 1.16E-03 |
| A_05_P462092 | 1.584 | 1.19E-04 | A_05_P291017 | 1.566 | 4.66E-02 |
| A_05_P327417 | 1.549 | 3.10E-04 | A_05_P291497 | 1.529 | 1.39E-03 |
| A_05_P473232 | 1.529 | 8.87E-03 | A_05_P399627 | 1.513 | 5.52E-03 |
| A_05_P356747 | 1.512 | 6.49E-03 | A_05_P357362 | 1.505 | 9.34E-04 |
| A_05_P453332 | 1.488 | 3.79E-02 | A_05_P301307 | 1.480 | 4.09E-02 |
| A_05_P295817 | 1.480 | 1.55E-02 | A_05_P265399 | 1.479 | 1.92E-02 |
| A_05_P340567 | 1.470 | 2.99E-02 | A_05_P353767 | 1.467 | 3.41E-03 |
| A_05_P261824 | 1.465 | 4.72E-03 | A_05_P399247 | 1.462 | 4.11E-04 |
| A_05_P275724 | 1.462 | 4.72E-02 | A_05_P356952 | 1.455 | 3.21E-04 |
| A_05_P307607 | 1.455 | 1.35E-03 | A_05_P472557 | 1.452 | 3.30E-02 |
| A_05_P343517 | 1.447 | 3.84E-02 | A_05_P324717 | 1.446 | 4.69E-02 |
| A_05_P315217 | 1.444 | 5.91E-03 | A_05_P268729 | 1.436 | 1.32E-02 |
| A_05_P301957 | 1.435 | 8.14E-04 | A_05_P259784 | 1.426 | 3.07E-02 |
| A_05_P256814 | 1.425 | 4.57E-03 | A_05_P306612 | 1.422 | 1.81E-03 |
| A_05_P383462 | 1.421 | 9.16E-03 | A_05_P420902 | 1.419 | 3.43E-02 |
| A_05_P258749 | 1.418 | 8.62E-03 | A_05_P307302 | 1.407 | 2.16E-03 |
| A_05_P331882 | 1.415 | 8.83E-03 | A_05_P285622 | 1.404 | 9.85E-03 |
| A_05_P338367 | 1.406 | 3.18E-02 | A_05_P421637 | 1.396 | 2.03E-02 |
| A_05_P295757 | 1.403 | 3.87E-03 | A_05_P451007 | 1.385 | 2.10E-03 |
| A_05_P302827 | 1.394 | 4.11E-02^c^ | A_05_P431077 | 1.380 | 1.75E-02 |
| A_05_P446612 | 1.382 | 3.81E-02 | A_05_P387132 | 1.378 | 2.93E-02 |
| A_05_P252544 | 1.375 | 8.05E-03 | A_05_P442882 | 1.369 | 1.57E-02 |
| A_05_P441232 | 1.369 | 1.49E-02 | A_05_P343142 | 1.367 | 7.54E-03 |
| A_05_P486372 | 1.362 | 8.83E-03 | A_05_P487737 | 1.361 | 9.35E-03 |
| A_05_P357077 | 1.359 | 1.12E-03 | A_05_P352617 | 1.358 | 1.17E-02 |
| A_05_P320917 | 1.357 | 1.04E-02 | A_05_P287052 | 1.356 | 1.72E-03 |
| A_05_P458277 | 1.353 | 4.56E-02 | A_05_P431877 | 1.352 | 7.77E-03 |
| A_05_P260814 | 1.350 | 2.89E-02 | A_05_P481572 | 1.350 | 3.93E-02 |
| A_05_P289452 | 1.346 | 2.55E-03 | A_05_P395272 | 1.344 | 4.73E-02 |
| A_05_P472407 | 1.343 | 4.29E-02 | A_05_P494372 | 1.339 | 1.02E-02 |
| A_05_P294117 | 1.339 | 1.48E-02 | A_05_P362712 | 1.334 | 4.91E-02 |
| A_05_P353922 | 1.332 | 1.36E-02 | A_05_P417802 | 1.331 | 1.73E-02 |
| A_05_P440982 | 1.330 | 4.81E-02 | A_05_P262424 | 1.328 | 9.74E-03 |
| A_05_P358632 | 1.328 | 2.50E-02 | A_05_P476477 | 1.327 | 1.82E-02 |
| A_05_P412952 | 1.327 | 4.25E-02 | A_05_P341467 | 1.327 | 3.34E-02 |
| A_05_P325182 | 1.327 | 3.78E-02 | A_05_P396032 | 1.326 | 4.63E-03 |
| A_05_P376437 | 1.320 | 3.29E-02 | A_05_P489392 | 1.322 | 4.38E-02 |
| A_05_P426982 | 1.318 | 1.16E-02 | A_05_P342562 | 1.318 | 1.46E-02 |
| A_05_P396022 | 1.318 | 1.45E-02 | A_05_P469707 | 1.316 | 1.37E-02 |
| A_05_P411172 | 1.316 | 3.57E-02 | A_05_P363497 | 1.316 | 4.91E-02 |
| A_05_P493432 | 1.313 | 4.70E-03 | A_05_P256144 | 1.312 | 7.81E-03 |
| A_05_P321017 | 1.312 | 4.20E-02 | A_05_P379487 | 1.310 | 2.07E-02 |
| A_05_P486962 | 1.309 | 3.83E-02 | A_05_P314597 | 1.309 | 3.04E-02 |
| A_05_P481252 | 1.306 | 3.56E-02 | A_05_P427022 | 1.303 | 2.64E-02 |
| A_05_P425232 | 1.303 | 4.99E-02 | A_05_P290137 | 1.302 | 1.02E-02 |
| A_05_P262569 | 1.300 | 2.12E-02 | A_05_P425547 | 1.300 | 3.68E-02 |
| A_05_P338597 | 1.299 | 2.87E-02 | A_05_P420382 | 1.298 | 1.48E-02 |
| A_05_P403012 | 1.297 | 8.22E-03 | A_05_P438487 | 1.291 | 7.74E-03 |
| A_05_P476322 | 1.290 | 1.28E-02 | A_05_P402592 | 1.290 | 5.00E-02 |
| A_05_P280937 | 1.289 | 6.69E-03 | A_05_P437907 | 1.288 | 1.17E-02 |
| A_05_P403107 | 1.287 | 6.97E-03 | A_05_P278567 | 1.287 | 3.89E-02 |
| A_05_P347542 | 1.286 | 3.49E-02 | A_05_P397432 | 1.285 | 9.92E-03 |
| A_05_P274929 | 1.283 | 2.44E-02 | A_05_P437067 | 1.282 | 1.84E-02 |
| A_05_P427952 | 1.282 | 1.13E-02 | A_05_P400507 | 1.281 | 1.74E-02 |
| A_05_P363152 | 1.281 | 2.30E-02 | A_05_P303327 | 1.279 | 1.99E-02 |
| A_05_P387262 | 1.279 | 1.45E-02 | A_05_P334292 | 1.277 | 4.08E-02 |
| A_05_P320717 | 1.277 | 2.31E-02 | A_05_P298442 | 1.274 | 2.64E-02 |
| A_05_P416362 | 1.272 | 4.78E-02 | A_05_P349362 | 1.272 | 2.52E-02 |
| A_05_P354612 | 1.271 | 3.35E-02 | A_05_P343527 | 1.271 | 2.87E-02 |
| A_05_P409922 | 1.270 | 2.36E-02 | A_05_P313967 | 1.269 | 3.29E-02 |
| A_05_P381147 | 1.269 | 3.91E-02 | A_05_P323637 | 1.268 | 8.68E-03 |
| A_05_P351362 | 1.267 | 2.64E-02 | A_05_P385592 | 1.267 | 2.81E-02 |
| A_05_P475452 | 1.266 | 1.72E-02 | A_05_P431492 | 1.265 | 2.24E-02 |
| A_05_P494702 | 1.264 | 4.45E-02 | A_05_P361297 | 1.264 | 4.92E-02 |
| A_05_P347452 | 1.263 | 3.19E-02 | A_05_P446332 | 1.260 | 4.29E-02 |
| A_05_P353507 | 1.260 | 4.99E-02 | A_05_P306252 | 1.259 | 1.71E-02 |
| A_05_P416679 | 1.377 | 5.69E-03 | A_05_P321732 | 1.258 | 2.54E-02 |
| A_05_P301762 | 1.256 | 2.51E-02 | A_05_P301952 | 1.255 | 8.62E-03 |
| A_05_P464522 | 1.252 | 4.42E-02 | A_05_P362077 | 1.250 | 2.10E-02 |
| A_05_P341117 | 1.250 | 3.91E-02 | A_05_P284617 | 1.249 | 9.65E-03 |
| A_05_P256404 | 1.249 | 2.71E-02 | A_05_P390037 | 1.248 | 1.02E-02 |
| A_05_P426247 | 1.248 | 2.57E-02 | A_05_P356042 | 1.245 | 2.89E-02 |
| A_05_P477307 | 1.245 | 3.08E-02 | A_05_P380707 | 1.242 | 1.85E-02 |
| A_05_P394462 | 1.241 | 4.17E-02 | A_05_P332482 | 1.240 | 4.64E-02 |
| A_05_P404842 | 1.240 | 4.98E-02 | A_05_P398387 | 1.238 | 4.61E-02 |
| A_05_P446067 | 1.235 | 1.72E-02 | A_05_P300292 | 1.232 | 4.48E-02 |
| A_05_P458227 | 1.221 | 3.90E-02 | A_05_P279122 | 1.221 | 3.87E-02 |
| A_05_P326137 | 1.217 | 2.18E-02 | A_05_P346737 | 1.215 | 4.96E-02 |
| A_05_P446742 | 1.213 | 2.70E-02 | A_05_P416727 | 1.212 | 4.93E-02 |
| A_05_P425862 | 1.211 | 4.29E-02 | A_05_P472102 | 1.204 | 4.90E-02 |
| A_05_P492857 | 1.257 | 1.36E-02 |  |  |  |
| ***Dec Fish*** |  |  | ***Dec Fish*** |  |  |
| A_05_P475697 | 1.89 | 3.33E-02 | A_05_P348167 | 1.877 | 2.72E-02^c^ |
| A_05_P454612 | 1.864 | 3.19E-02 | A_05_P323672 | 1.858 | 4.31E-03 |
| A_05_P446242 | 1.846 | 1.07E-02 | A_05_P489142 | 1.766 | 1.45E-02 |
| A_05_P302427 | 1.738 | 3.80E-02 | A_05_P337842 | 1.716 | 4.63E-02 |
| A_05_P316827 | 1.700 | 3.82E-02 | A_05_P298102 | 1.696 | 4.78E-02 |
| A_05_P359332 | 1.679 | 3.77E-02 | A_05_P479022 | 1.673 | 4.65E-02 |
| A_05_P405962 | 1.642 | 3.67E-02 | A_05_P269824 | 1.642 | 2.82E-02 |
| A_05_P293452 | 1.634 | 1.82E-02 | A_05_P460467 | 1.633 | 1.18E-02 |
| A_05_P393517 | 1.602 | 4.91E-02 | A_05_P432912 | 1.575 | 4.51E-02 |
| A_05_P364662 | 1.571 | 1.25E-02 | A_05_P341132 | 1.570 | 2.69E-02 |
| A_05_P335122 | 1.560 | 7.11E-03 | A_05_P354417 | 1.555 | 4.09E-02 |
| A_05_P427477 | 1.552 | 3.60E-02 | A_05_P450263 | 1.547 | 4.83E-02 |
| A_05_P394442 | 1.533 | 3.08E-02 | A_05_P327417 | 1.520 | 1.86E-02 |
| A_05_P486157 | 1.508 | 1.51E-02 | A_05_P318357 | 1.500 | 2.70E-02 |
| A_05_P384412 | 1.490 | 3.50E-02 | A_05_P291722 | 1.487 | 2.78E-02 |
| A_05_P319207 | 1.485 | 3.09E-02 | A_05_P354567 | 1.484 | 2.02E-02 |
| A_05_P311402 | 1.482 | 3.18E-02 | A_05_P336632 | 1.460 | 2.85E-02 |
| A_05_P492452 | 1.456 | 3.17E-02 | A_05_P472897 | 1.447 | 2.81E-02 |
| A_05_P290752 | 1.433 | 4.00E-02 | A_05_P405072 | 1.425 | 3.46E-02 |
| A_05_P347867 | 1.419 | 3.20E-02 | A_05_P269744 | 1.419 | 3.72E-02 |
| A_05_P479927 | 1.406 | 3.96E-02 | A_05_P396652 | 1.405 | 3.80E-02 |
| A_05_P432792 | 1.403 | 4.48E-02 | A_05_P435507 | 1.388 | 4.15E-02 |
| A_05_P288037 | 1.378 | 4.76E-02^c^ | A_05_P447852 | 1.377 | 4.88E-02 |
| A_05_P335272 | 1.366 | 4.89E-02 | A_05_P287092 | 1.362 | 4.80E-02^c^ |

^a^Fold change is the average difference in expression as measured by the microarray

^b^ Measures the significance of the difference in expression between the small and large fish with a t-test

**c** Guassian p-value < 0.05, t-test p-value > 0.05

Genes with significant up-regulation in small fish across seasons are highlighted in green
