# Supplementary material for: Differential gene expression in small and large rainbow trout derived from two seasonal spawning groups
Source: BMC Genomics. 2014 Jan 22;15:57. doi: 10.1186/1471-2164-15-57 (PMC3931318; doi:10.1186/1471-2164-15-57)
Supplement: Additional file 9: Table S9 — Genes up-regulated in the white muscle of large rainbow trout compared to small rainbow trout. [file 1471-2164-15-57-S9.docx]

| **Supplementary Table 9: Genes up-regulated in the white muscle of large rainbow trout compared to small rainbow trout** | | | |
| --- | --- | --- | --- |
| **Gene Name** | **Gene Number** | **Fold change^a^** | **p-value^b^** |
| ***Sept fish*** |  |  |  |
| max protein^e^ | A_05_P478412 | 5.618 | 4.14E-03 |
| DNA-damage-inducible transcript 4-like protein | A_05_P249334 | 5.128 | 9.71E-04 |
| ADP/ATP translocase 2 | A_05_P276614 | 4.000 | 8.42E-03 |
| complement C1q-like protein 4 precursor^c^ | A_05_P332842 | 3.891 | 9.94E-03 |
| hig1 domain family member 2a^d^ | A_05_P433247 | 2.801 | 3.92E-02 |
| polycystic kidney disease protein 1-like 3 precursor^c^ | A_05_P299292 | 2.717 | 7.16E-03 |
| c-type lectin | A_05_P249684 | 2.646 | 1.13E-02 |
| fibrinogen gamma chain | A_05_P480537 | 2.625 | 3.06E-02 |
| fibrinogen gamma chain | A_05_P364872 | 2.584 | 3.24E-02 |
| 40s ribosomal protein s30^d^ | A_05_P377337 | 2.558 | 7.26E-03 |
| cathepsin m precursor | A_05_P251889 | 2.525 | 8.39E-03 |
| fibrinogen alpha chain | A_05_P332052 | 2.506 | 1.24E-02 |
| beta-enolase-like isoform 1 | A_05_P482637 | 2.500 | 4.06E-02 |
| retinol-binding protein 2 | A_05_P254734 | 2.481 | 2.03E-02 |
| fibrinogen gamma chain | A_05_P464657 | 2.463 | 1.82E-02 |
| trichohyalin^c^ | A_05_P439982 | 2.433 | 2.17E-02 |
| complement C1q-like protein 2 precursor^c^ | A_05_P252174 | 2.404 | 1.23E-02 |
| pyruvate kinase | A_05_P253514 | 2.381 | 2.45E-02 |
| inter-alpha-trypsin inhibitor heavy chain h3-like | A_05_P267179 | 2.315 | 7.17E-03 |
| zinc-binding protein a33-like | A_05_P254724 | 2.309 | 5.51E-03 |
| C-reactive protein precursor^c^ | A_05_P268064 | 2.278 | 1.96E-02 |
| myosin-2 heavy chain, non muscle^c^ | A_05_P277062 | 2.268 | 1.23E-02 |
| warm temperature acclimation-related 65 kda protein | A_05_P420737 | 2.227 | 5.84E-03 |
| mannose-binding lectin-associated serine protease-3b^d^ | A_05_P475232 | 2.188 | 1.95E-02 |
| alpha-1-microglobulin bikunin precursor | A_05_P332632 | 2.137 | 3.93E-02 |
| serum amyloid P-component precursor^c^ | A_05_P453307 | 2.114 | 9.13E-03 |
| liver-type fatty acid-binding protein | A_05_P454457 | 2.066 | 2.85E-02 |
| secreted phosphoprotein 24 precursor | A_05_P248944 | 2.062 | 4.09E-02 |
| fibrinogen gamma polypeptide | A_05_P450362 | 2.045 | 4.47E-02 |
| complement component c3 | A_05_P433922 | 2.024 | 2.44E-02 |
| pyruvate kinase | A_05_P390772 | 2.004 | 2.52E-02 |
| warm temperature acclimation-related 65 kda protein^d^ | A_05_P251959 | 1.949 | 1.01E-02 |
| liver-type fatty acid-binding protein | A_05_P263874 | 1.949 | 4.58E-02 |
| serum amyloid P-component precursor^c^ | A_05_P492482 | 1.938 | 1.43E-02 |
| serum albumin precursor | A_05_P368112 | 1.934 | 3.96E-02 |
| coagulation factor ii precursor^d^ | A_05_P364912 | 1.919 | 2.10E-02 |
| Ca2+-dependent complex c1r c1s subunit | A_05_P457602 | 1.919 | 3.93E-02 |
| beta-2-glycoprotein 1-like | A_05_P275734 | 1.890 | 3.57E-02 |
| retinol binding protein cellular | A_05_P486737 | 1.890 | 2.23E-02 |
| liver basic fatty acid binding protein | A_05_P455512 | 1.887 | 4.92E-02 |
| troponin I, fast skeletal muscle^c^ | A_05_P249354 | 1.883 | 2.29E-02 |
| cytochrome b | A_05_P486612 | 1.883 | 2.76E-02 |
| hp protein | A_05_P365477 | 1.859 | 3.25E-02 |
| DNA replication licensing factor mcm7 | A_05_P266444 | 1.838 | 1.63E-02 |
| complement c4 | A_05_P252169 | 1.832 | 1.91E-02 |
| apolipoprotein a-i | A_05_P249389 | 1.786 | 1.51E-02 |
| hemoglobin subunit alpha^c^ | A_05_P491417 | 1.776 | 1.43E-02 |
| extracellular matrix protein 1 | A_05_P268474 | 1.773 | 1.03E-02 |
| apolipoprotein b-100 | A_05_P486052 | 1.764 | 1.71E-02 |
| complement component c9 | A_05_P485237 | 1.748 | 4.63E-02 |
| hemoglobin subunit alpha^d^ | A_05_P449312 | 1.745 | 1.45E-02 |
| myosin light chain 1 | A_05_P485207 | 1.742 | 4.83E-02 |
| t-complex protein 1 subunit epsilon | A_05_P249199 | 1.712 | 3.78E-02 |
| hemopexin precursor^c^ | A_05_P275779 | 1.709 | 1.14E-02 |
| c1 inhibitor^d^ | A_05_P475617 | 1.709 | 2.09E-02 |
| Telethonin | A_05_P263889 | 1.695 | 1.79E-02 |
| ap-1 complex subunit gamma-1 | A_05_P427757 | 1.686 | 3.83E-02 |
| hemoglobin subunit beta-1 | A_05_P453042 | 1.686 | 1.69E-02 |
| myozenin-2 | A_05_P265999 | 1.684 | 2.09E-02 |
| hemoglobin subunit alpha^d^ | A_05_P249524 | 1.664 | 3.55E-02 |
| apolipoprotein b 100 | A_05_P255119 | 1.658 | 2.66E-02 |
| enolase 3-2^d^ | A_05_P473402 | 1.656 | 2.50E-02 |
| alpha 2a pancreatic | A_05_P249709 | 1.645 | 3.73E-02 |
| transmembrane protein 100-like | A_05_P318762 | 1.639 | 2.56E-02 |
| vacuolar protein sorting 52 | A_05_P292107 | 1.637 | 3.30E-02 |
| Telethonin | A_05_P413737 | 1.616 | 3.15E-02 |
| lactate dehydrogenase-a | A_05_P469692 | 1.616 | 2.02E-02 |
| formiminotransferase cyclodeaminase | A_05_P252304 | 1.592 | 2.42E-02 |
| glutathione peroxidase | A_05_P365217 | 1.585 | 2.46E-02 |
| alpha-2-hs-glycoprotein precursor | A_05_P364887 | 1.580 | 4.72E-02 |
| beta-2-glycoprotein 1 precursor^c^ | A_05_P442607 | 1.555 | 2.87E-02 |
| lon peptidase n-terminal domain and ring finger protein 2 | A_05_P482212 | 1.553 | 4.37E-02 |
| hemoglobin subunit beta-1 | A_05_P365862 | 1.550 | 4.28E-02 |
| c1 inhibitor^d^ | A_05_P265959 | 1.548 | 4.31E-02 |
| Ceruloplasmin | A_05_P365482 | 1.536 | 4.43E-02 |
| antithrombin-iii precursor | A_05_P445252 | 1.527 | 4.36E-02 |
| circumsporozoite protein precursor^c^ | A_05_P482807 | 1.490 | 4.44E-02 |
| rap1 gtpase-activating protein 2-like | A_05_P324407 | 1.479 | 3.46E-02 |
| mgc82112 protein^d^ | A_05_P250149 | 1.477 | 4.91E-02 |
| cysteine-rich motor neuron 2 protein precursor^c^ | A_05_P310592 | 1.473 | 4.34E-02 |
| fibronectin precursor | A_05_P263834 | 1.458 | 2.50E-02 |
| alpha-l-iduronidase precursor | A_05_P457567 | 1.445 | 2.67E-02 |
| complement c3-like | A_05_P490817 | 1.439 | 4.24E-02 |
| ribosomal protein s13 | A_05_P471392 | 1.437 | 3.20E-02 |
| mid1-interacting protein 1^c^ | A_05_P364592 | 1.429 | 3.41E-02 |
| interferon-induced 6-16 family^c^ | A_05_P270409 | 1.420 | 3.47E-02 |
| angiotensinogen precursor | A_05_P333067 | 1.420 | 3.73E-02 |
| zona pellucida sperm-binding protein 3 precursor^c^ | A_05_P377297 | 1.387 | 4.37E-02 |
| antithrombin-iii precursor | A_05_P249134 | 1.346 | 2.91E-02 |
| ***Dec Fish*** |  |  |  |
| fibrinogen gamma polypeptide | A_05_P450362 | 25.126 | 1.71E-02 |
| c1 inhibitor^e^ | A_05_P475617 | 4.717 | 3.85E-02 |
| hig1 domain family member 2a^d^ | A_05_P368647 | 4.049 | 3.85E-02 |
| mgc82112 protein^e^ | A_05_P250149 | 3.831 | 2.60E-02 |
| transcription factor jun-b | A_05_P266894 | 3.759 | 3.03E-02 |
| butyrate response factor 1^d^ | A_05_P376337 | 3.559 | 1.17E-02 |
| fucolectin-4 precursor^d^ | A_05_P463327 | 3.436 | 4.43E-02 |
| iron zinc purple acid phosphatase-like | A_05_P411342 | 3.344 | 4.52E-02 |
| Transketolase | A_05_P414317 | 3.333 | 4.38E-02 |
| lysosome membrane protein 2-like | A_05_P413782 | 3.247 | 3.45E-02 |
| lactate dehydrogenase b | A_05_P444697 | 3.115 | 4.38E-02 |
| eukaryotic translation elongation factor 1 alpha 1 | A_05_P362822 | 2.976 | 3.11E-02 |
| acidic mammalian chitinase-like | A_05_P391302 | 2.941 | 3.58E-02 |
| growth arrest and dna-damage- beta | A_05_P409552 | 2.841 | 1.08E-02 |
| lipoprotein lipase | A_05_P429962 | 2.778 | 1.58E-02 |
| neutral alpha-glucosidase ab-like | A_05_P259469 | 2.688 | 3.22E-02 |
| max protein^d^ | A_05_P478412 | 2.681 | 2.36E-02 |
| metallothionein^c^ | A_05_P249474 | 2.674 | 2.70E-02 |
| delta-6 fatty acyl desaturase^d^ | A_05_P248839 | 2.653 | 4.06E-02 |
| flavin reductase | A_05_P418547 | 2.632 | 2.75E-02 |
| eukaryotic translation elongation factor 1 alpha 1 | A_05_P449262 | 2.618 | 3.06E-02 |
| Ictacalcin | A_05_P488907 | 2.611 | 1.49E-02 |
| liver-expressed antimicrobial peptide 2 | A_05_P475047 | 2.571 | 3.33E-02 |
| bolA-like protein 2^c^ | A_05_P336112 | 2.564 | 1.38E-02 |
| annexin a1 | A_05_P287152 | 2.506 | 1.91E-02 |
| cathepsin d | A_05_P251434 | 2.500 | 1.02E-02 |
| purine nucleoside phosphorylase | A_05_P415112 | 2.494 | 2.04E-02 |
| eukaryotic translation elongation factor 1 alpha 1 | A_05_P454907 | 2.488 | 3.53E-02 |
| heat shock protein 90 | A_05_P249734 | 2.457 | 3.06E-02 |
| litaf-like protein | A_05_P486262 | 2.457 | 4.47E-02 |
| alpha actin | A_05_P423987 | 2.451 | 1.28E-02 |
| xylose isomerase | A_05_P277217 | 2.439 | 1.64E-02 |
| middle subunit | A_05_P434332 | 2.427 | 3.47E-02 |
| uncharacterized protein C8orf4 homolog^c^ | A_05_P262634 | 2.410 | 2.26E-02 |
| arylacetamide deacetylase-like 1 | A_05_P414357 | 2.387 | 1.58E-02 |
| kelch-like protein 20 | A_05_P345882 | 2.375 | 1.77E-02 |
| heat shock protein 90 | A_05_P409047 | 2.375 | 3.56E-02 |
| small cytokines (intecrine/chemokine), interleukin-8 like^c^ | A_05_P412367 | 2.358 | 3.35E-02 |
| early nodulin 75 protein^c^ | A_05_P465727 | 2.353 | 3.49E-02 |
| formin homology region 1^c^ | A_05_P272774 | 2.309 | 2.53E-02 |
| glutamate dehydrogenase | A_05_P489712 | 2.304 | 4.60E-02 |
| growth factor receptor-bound protein 14 | A_05_P434562 | 2.299 | 2.92E-02 |
| adipocyte plasma membrane-associated protein | A_05_P431137 | 2.288 | 1.67E-02 |
| annexin a11 | A_05_P369052 | 2.273 | 3.45E-02 |
| aspartyl asparaginyl beta-hydroxylase-like | A_05_P383437 | 2.268 | 4.33E-02 |
| keratin 18 | A_05_P407317 | 2.268 | 2.58E-02 |
| actin-related protein 2 3 complex subunit 1b | A_05_P250804 | 2.262 | 3.14E-02 |
| putative serine protease K12H4.7 precursor^c^ | A_05_P303397 | 2.232 | 4.43E-02 |
| dna-damage-inducible transcript 4-like protein | A_05_P249334 | 2.227 | 2.26E-02 |
| beta-1-syntrophin | A_05_P264564 | 2.227 | 4.33E-02 |
| collagen type x alpha partial | A_05_P336802 | 2.227 | 2.53E-02 |
| type i keratin e7^d^ | A_05_P367169 | 2.227 | 4.99E-02 |
| adipose differentiation-related protein^d^ | A_05_P427297 | 2.222 | 3.39E-02 |
| glutathione s-transferase theta-1 | A_05_P408522 | 2.203 | 4.04E-02 |
| transcription factor jun-b | A_05_P410847 | 2.203 | 1.95E-02 |
| keratin 19^d^ | A_05_P454752 | 2.198 | 2.22E-02 |
| cd302 antigen precursor | A_05_P371252 | 2.193 | 4.79E-02 |
| 40s ribosomal protein s2 | A_05_P366007 | 2.188 | 1.52E-02 |
| interferon-induced transmembrane protein^c^ | A_05_P275264 | 2.160 | 1.42E-02 |
| glutamate decarboxylase-like protein 1 | A_05_P409992 | 2.151 | 1.42E-02 |
| Thioredoxin | A_05_P465152 | 2.151 | 4.19E-02 |
| fatty acid-binding heart | A_05_P263974 | 2.119 | 3.61E-02 |
| ubiquitin-conjugating enzyme e2 d2 | A_05_P273004 | 2.119 | 1.18E-02 |
| middle subunit | A_05_P377417 | 2.119 | 2.62E-02 |
| vasodilator-stimulated phosphoprotein | A_05_P471632 | 2.119 | 4.94E-02 |
| translocating chain-associated membrane protein 2 | A_05_P366322 | 2.110 | 4.84E-02 |
| type i iodothyronine deiodinase | A_05_P437087 | 2.105 | 3.62E-02 |
| fatty acid-binding heart | A_05_P424767 | 2.101 | 2.56E-02 |
| Ig mu chain C region membrane-bound form^c^ | A_05_P403197 | 2.092 | 1.28E-02 |
| epididymal secretory protein e1 precursor | A_05_P424497 | 2.092 | 3.11E-02 |
| collagenase 3 precursor | A_05_P327062 | 2.088 | 2.05E-02 |
| nadp-dependent malic enzyme | A_05_P419472 | 2.083 | 3.02E-02 |
| putative lymphocyte G0/G1 switch protein 2^c^ | A_05_P364577 | 2.075 | 1.23E-02 |
| isochorismatase domain-containing protein mitochondrial precursor | A_05_P471182 | 2.075 | 2.86E-02 |
| c-type lysozyme | A_05_P364387 | 2.058 | 3.08E-02 |
| calcium and integrin-binding protein 1 | A_05_P271234 | 2.053 | 4.18E-02 |
| beta-2-microglobulin precursor | A_05_P249274 | 2.049 | 2.19E-02 |
| glutamate dehydrogenase | A_05_P246334 | 2.045 | 2.41E-02 |
| ap-1 complex subunit gamma-1 | A_05_P427757 | 2.041 | 1.14E-02 |
| thioredoxin-interacting protein | A_05_P364437 | 2.037 | 3.55E-02 |
| transmembrane protein 50a | A_05_P407172 | 2.037 | 3.59E-02 |
| calponin 2 | A_05_P411697 | 2.024 | 1.58E-02 |
| sequestosome 1 | A_05_P453857 | 2.016 | 3.60E-02 |
| Peflin | A_05_P434382 | 2.012 | 2.15E-02 |
| heat shock protein 90 | A_05_P407712 | 2.008 | 3.33E-02 |
| serine incorporator 1 | A_05_P417282 | 2.004 | 3.83E-02 |
| ef-hand domain-containing protein d2 | A_05_P251544 | 1.996 | 2.16E-02 |
| kruppel-like factor 2 | A_05_P414782 | 1.992 | 3.03E-02 |
| uridine-cytidine kinase 1 | A_05_P441157 | 1.984 | 3.67E-02 |
| cofilin-2 | A_05_P414627 | 1.976 | 2.81E-02 |
| gdp dissociation inhibitor 2 | A_05_P424192 | 1.976 | 4.65E-02 |
| flna protein | A_05_P251164 | 1.972 | 3.79E-02 |
| NADH dehydrogenase | A_05_P253124 | 1.972 | 1.72E-02 |
| ADP/ATP translocase 2 | A_05_P276614 | 1.972 | 2.82E-02 |
| high mobility group-t protein | A_05_P340112 | 1.957 | 4.62E-02 |
| nedd4 family-interacting protein 2 | A_05_P437412 | 1.957 | 3.23E-02 |
| membrane-associated transporter protein | A_05_P426482 | 1.953 | 1.40E-02 |
| aminopeptidase n-like | A_05_P490602 | 1.953 | 4.54E-02 |
| serine protease 23 precursor | A_05_P425157 | 1.942 | 4.93E-02 |
| ADP-ribosylation factor 5^d^ | A_05_P419712 | 1.938 | 2.27E-02 |
| cathepsin b precursor | A_05_P425312 | 1.938 | 1.71E-02 |
| nf-kappa-b inhibitor zeta | A_05_P301692 | 1.919 | 2.08E-02 |
| matrix metalloproteinase 2^d^ | A_05_P409312 | 1.919 | 3.29E-02 |
| ubiquitin-conjugating enzyme e2 d2 | A_05_P459017 | 1.919 | 4.49E-02 |
| nedd4 family-interacting protein 1 | A_05_P369097 | 1.916 | 3.72E-02 |
| 40S ribosomal protein S27^c^ | A_05_P475507 | 1.905 | 2.00E-02 |
| arachidonate 5-lipoxygenase-activating protein | A_05_P404592 | 1.901 | 2.35E-02 |
| h-2 class ii histocompatibility antigen gamma chain | A_05_P416477 | 1.901 | 3.40E-02 |
| leukocyte surface antigen cd53 | A_05_P251864 | 1.898 | 3.94E-02 |
| protein phosphatase 1 regulatory subunit 1b | A_05_P474467 | 1.883 | 2.77E-02 |
| Ig mu chain C region membrane-bound form^c^ | A_05_P250274 | 1.880 | 1.42E-02 |
| Na+ K+ alpha 1 polypeptide | A_05_P390667 | 1.873 | 4.22E-02 |
| proteasome subunit beta type-9 precursor | A_05_P459622 | 1.869 | 2.08E-02 |
| glutathione s-transferase p | A_05_P470422 | 1.869 | 2.46E-02 |
| coagulation factor vii^d^ | A_05_P333597 | 1.859 | 2.08E-02 |
| tsc22 domain member 3 | A_05_P457747 | 1.859 | 5.00E-02 |
| fatty acid-binding heart | A_05_P249654 | 1.838 | 3.81E-02 |
| plasminogen activator inhibitor 1 precursor | A_05_P285012 | 1.821 | 2.52E-02 |
| dystonin isoform 2^d^ | A_05_P440912 | 1.815 | 4.53E-02 |
| glutaryl-coenzyme a dehydrogenase | A_05_P367057 | 1.805 | 4.97E-02 |
| coactosin-like 1 | A_05_P369682 | 1.805 | 1.77E-02 |
| ADP-ribosylation factor 4 | A_05_P413937 | 1.805 | 4.87E-02 |
| probable e3 ubiquitin-protein ligase mycbp2 | A_05_P420912 | 1.805 | 4.68E-02 |
| transcription factor jun-d | A_05_P473177 | 1.802 | 3.29E-02 |
| collagen alpha-1 chain-like | A_05_P335067 | 1.799 | 4.26E-02 |
| sh3 domain-binding glutamic acid-rich-like protein 3 | A_05_P457412 | 1.792 | 3.77E-02 |
| erythrocyte membrane protein band -like 2^d^ | A_05_P258819 | 1.789 | 2.60E-02 |
| ccaat enhancer-binding protein beta | A_05_P412813 | 1.789 | 2.49E-02 |
| matrix metalloproteinase 2^d^ | A_05_P249954 | 1.773 | 3.68E-02 |
| retinoid x receptor beta | A_05_P436752 | 1.773 | 3.03E-02 |
| nuclear pore complex protein nup133 | A_05_P304537 | 1.770 | 2.19E-02 |
| Latexin | A_05_P407002 | 1.761 | 2.49E-02 |
| carnitine acetyltransferase | A_05_P453022 | 1.757 | 3.53E-02 |
| platelet-derived growth factor receptor-like | A_05_P254039 | 1.754 | 4.15E-02 |
| ras GTPase-activating-like protein iqgap1 | A_05_P408317 | 1.754 | 3.69E-02 |
| splicing arginine serine-rich 7 | A_05_P447952 | 1.754 | 4.53E-02 |
| ATP citrate lyase | A_05_P411167 | 1.751 | 2.43E-02 |
| cytochrome b- beta polypeptide^d^ | A_05_P255974 | 1.748 | 3.86E-02 |
| collagen type i alpha 1 | A_05_P430717 | 1.748 | 4.01E-02 |
| selenoprotein u^d^ | A_05_P420492 | 1.745 | 3.00E-02 |
| microtubule-associated protein 1 light chain 3 beta | A_05_P415832 | 1.742 | 3.45E-02 |
| phosphoglycerate mutase 2 | A_05_P431037 | 1.742 | 1.81E-02 |
| cathepsin l | A_05_P364557 | 1.739 | 3.68E-02 |
| aspartate beta-hydroxylase | A_05_P466432 | 1.739 | 4.47E-02 |
| glutathione peroxidise | A_05_P389542 | 1.730 | 4.82E-02 |
| 40s ribosomal protein s5 | A_05_P250994 | 1.715 | 3.11E-02 |
| methionine-r-sulfoxide reductase mitochondrial like | A_05_P273554 | 1.715 | 3.97E-02 |
| collagen type i alpha 2 | A_05_P413407 | 1.709 | 2.85E-02 |
| mannose-binding 2^d^ | A_05_P412842 | 1.701 | 3.02E-02 |
| protein transport protein sec24d | A_05_P370467 | 1.698 | 3.59E-02 |
| hydroxypyruvate isomerise | A_05_P265969 | 1.692 | 4.60E-02 |
| t- immune regulator 1^d^ | A_05_P389977 | 1.689 | 3.21E-02 |
| u4 tri-snrnp-associated protein 2 | A_05_P381297 | 1.684 | 2.77E-02 |
| glioblastoma amplified sequence^d^ | A_05_P369487 | 1.681 | 4.95E-02 |
| brain protein I3^c^ | A_05_P455412 | 1.681 | 4.08E-02 |
| proline-rich nuclear receptor coactivator 2 | A_05_P436962 | 1.672 | 4.25E-02 |
| 60s ribosomal protein l21 | A_05_P388122 | 1.667 | 3.95E-02 |
| OTU domain-containing protein 3^c^ | A_05_P426682 | 1.664 | 4.25E-02 |
| hematological and neurological expressed 1 protein^c^ | A_05_P452873 | 1.664 | 2.94E-02 |
| transmembrane protein 33 | A_05_P448652 | 1.650 | 4.08E-02 |
| Ig mu chain C region membrane-bound form^c^ | A_05_P414707 | 1.645 | 3.35E-02 |
| transcriptional repressor ctcf-like | A_05_P420422 | 1.639 | 3.16E-02 |
| type alpha 2^d^ | A_05_P377682 | 1.637 | 4.34E-02 |
| vacuolar atp synthase 16 kda proteolipid subunit | A_05_P413453 | 1.637 | 3.79E-02 |
| grancalcin | A_05_P418347 | 1.634 | 3.58E-02 |
| selenium-binding protein 1 | A_05_P272174 | 1.631 | 2.76E-02 |
| proline-rich nuclear receptor coactivator 2 | A_05_P424262 | 1.629 | 3.11E-02 |
| heme oxygenase | A_05_P253919 | 1.623 | 4.97E-02 |
| ferritin high chain | A_05_P307532 | 1.613 | 4.73E-02 |
| ubr3 protein | A_05_P414617 | 1.613 | 5.00E-02 |
| protein bat5 | A_05_P421177 | 1.608 | 4.89E-02 |
| choline-phosphate cytidylyltransferase a | A_05_P433032 | 1.605 | 3.97E-02 |
| thymosin beta-12 | A_05_P436397 | 1.605 | 4.86E-02 |
| isoform cra_a^d^ | A_05_P439867 | 1.605 | 4.15E-02 |
| caprin-1-like isoform 2 | A_05_P410832 | 1.600 | 4.03E-02 |
| arp2 actin-related protein 2 homolog | A_05_P458032 | 1.577 | 4.64E-02 |
| h2a histone member y2 | A_05_P414427 | 1.575 | 4.53E-02 |
| ubiquitin fusion degradation protein 1 homolog | A_05_P261369 | 1.572 | 4.80E-02 |
| ubiquitin-conjugating enzyme e2 r1 | A_05_P473022 | 1.572 | 4.82E-02 |
| proline-rich nuclear receptor coactivator 1 | A_05_P379727 | 1.570 | 4.84E-02 |
| polyadenylate-binding protein-interacting protein 2 | A_05_P411962 | 1.567 | 4.79E-02 |
| lactate dehydrogenase-a | A_05_P469692 | 1.565 | 4.24E-02 |
| probable atp-dependent rna helicase ddx5 | A_05_P480527 | 1.565 | 4.34E-02 |
| Moesin | A_05_P366287 | 1.563 | 3.78E-02 |
| nucleolysin tiar isoform 1 | A_05_P435862 | 1.563 | 4.95E-02 |
| latent-transforming growth factor beta-binding protein 1 precursor | A_05_P424867 | 1.555 | 4.45E-02 |
| nedd4 family-interacting protein 1 | A_05_P419047 | 1.553 | 4.53E-02 |
| protein kinase c inhibitor aswz variant 5^d^ | A_05_P274604 | 1.527 | 4.75E-02 |
| b-cell translocation gene anti-proliferative | A_05_P415552 | 1.522 | 3.91E-02 |
| tetraspanin-8 | A_05_P429832 | 1.522 | 3.68E-02 |
| methylmalonate-semialdehyde dehydrogenase | A_05_P413822 | 1.504 | 4.59E-02 |
| protein c20orf11 homolog | A_05_P419862 | 1.495 | 4.04E-02 |
| cystathionine beta-synthase | A_05_P269379 | 1.484 | 4.83E-02 |
| 40s ribosomal protein s5 | A_05_P249309 | 1.473 | 4.93E-02 |
| cd63 antigen | A_05_P263177 | 1.466 | 3.98E-02 |
| farnesyl pyrophosphate synthetase | A_05_P266579 | 1.460 | 4.10E-02 |
| atlastin 3 | A_05_P375827 | 1.422 | 4.95E-02 |
| adenylosuccinate synthase like 1 | A_05_P265444 | 1.420 | 4.92E-02 |
| pdz and lim domain protein 3 | A_05_P248884 | 1.406 | 3.38E-02 |

^a^Fold change is the average difference in expression as measured by the microarray

^b^ Measures the significance of the difference in expression between the small and large fish.

^c^ Sequence was unnamed by Blast2go but named by Agilent

^d^ Identified as a different gene by Agilent

Genes with significant up-regulation in large fish across seasons are highlighted in green

Genes with significant up-regulation in large fish (but different probe Ids) across seasons are highlighted in yellow
