# Supplementary material for: Differential gene expression in small and large rainbow trout derived from two seasonal spawning groups
Source: BMC Genomics. 2014 Jan 22;15:57. doi: 10.1186/1471-2164-15-57 (PMC3931318; doi:10.1186/1471-2164-15-57)
Supplement: Additional file 10: Table S10 — Genes up-regulated in the white muscle of small rainbow trout compared to large rainbow trout. [file 1471-2164-15-57-S10.docx]

| **Supplementary Table 10: Genes up-regulated in the white muscle of small rainbow trout compared to large rainbow trout** | | | |
| --- | --- | --- | --- |
| **Gene Name** | **Gene Number** | **Fold change^a^** | **p-value^b^** |
| ***Sept Fish*** |  |  |  |
| parvalbumin 2 | A_05_P423182 | 6.722 | 8.14E-04 |
| calcium-binding and coiled-coil domain-containing protein 1 isoform 2 | A_05_P276634 | 5.305 | 3.25E-05 |
| parvalbumin beta-2 | A_05_P429727 | 3.595 | 1.67E-03 |
| glyceraldehyde-3-phosphate dehydrogenase | A_05_P419277 | 3.542 | 1.31E-04 |
| thrombospondin 2 | A_05_P322687 | 3.014 | 2.58E-02 |
| calcium binding and coiled-coil domain like^c^ | A_05_P276654 | 2.971 | 8.67E-04 |
| myosin heavy chain | A_05_P393052 | 2.682 | 1.37E-02 |
| alpha actin | A_05_P449267 | 2.550 | 9.65E-04 |
| creatine kinase | A_05_P442387 | 2.107 | 3.18E-02 |
| RNA-binding protein 45 | A_05_P441127 | 2.066 | 1.49E-02 |
| response gene to complement 32 protein | A_05_P268239 | 2.059 | 8.77E-04 |
| tyrosine-protein phosphatase non-receptor type 12 | A_05_P478272 | 2.013 | 7.26E-03 |
| myelin basic protein | A_05_P368497 | 1.867 | 5.38E-04 |
| creatine kinase | A_05_P388107 | 1.853 | 1.16E-02 |
| acyl- dehydrogenase –like^d^ | A_05_P445852 | 1.827 | 1.24E-02 |
| calcium calmodulin-dependent protein kinase (kinase) ii delta 2 | A_05_P304402 | 1.819 | 3.67E-02 |
| myelin basic protein | A_05_P472647 | 1.768 | 8.90E-03 |
| peptidase (mitochondrial processing) alpha | A_05_P378807 | 1.750 | 3.03E-02 |
| peripheral myelin protein 22 | A_05_P413907 | 1.674 | 1.62E-02 |
| 80 kda mcm3-associated protein | A_05_P351672 | 1.667 | 2.15E-02 |
| nuclease harbi1-like^d^ | A_05_P419577 | 1.651 | 2.97E-03 |
| heterogeneous nuclear ribonucleoprotein g | A_05_P432852 | 1.621 | 6.06E-03 |
| eukaryotic translation initiation factor 5b | A_05_P420767 | 1.608 | 4.86E-02 |
| septin 9 | A_05_P324627 | 1.605 | 1.32E-02 |
| myelin protein zero | A_05_P393932 | 1.601 | 2.88E-02 |
| 60s ribosomal export protein nmd3 | A_05_P411892 | 1.592 | 4.21E-02 |
| guanidinoacetate n-methyltransferase | A_05_P326907 | 1.567 | 3.73E-03 |
| voltage-dependent anion-selective channel protein 2 | A_05_P411937 | 1.560 | 3.30E-02 |
| ribosomal protein l3-like | A_05_P276644 | 1.557 | 1.81E-03 |
| 39s ribosomal protein mitochondrial precursor | A_05_P337442 | 1.556 | 5.34E-03 |
| pre-rrna-processing protein tsr1 homolog | A_05_P449717 | 1.552 | 3.93E-03 |
| 6- muscle type | A_05_P467772 | 1.532 | 2.06E-02 |
| aspartyl-tRNA synthetase | A_05_P413257 | 1.502 | 3.35E-02 |
| myelin proteolipid protein | A_05_P450522 | 1.495 | 3.20E-03 |
| DNA polymerase III subunits gamma and tau^c^ | A_05_P378092 | 1.493 | 9.33E-03 |
| chaperonin containing subunit 6a (zeta 1) | A_05_P412867 | 1.492 | 6.60E-03 |
| alpha 8 like 2^d^ | A_05_P453422 | 1.477 | 1.98E-02 |
| RING finger protein 170^c^ | A_05_P462112 | 1.471 | 2.88E-02 |
| vesicle-associated membrane protein-associated protein a | A_05_P260534 | 1.467 | 4.49E-02 |
| protein fam13a | A_05_P332747 | 1.467 | 2.42E-02 |
| myelin basic protein | A_05_P375352 | 1.459 | 1.02E-03 |
| proteasome maturation protein | A_05_P441892 | 1.457 | 5.16E-03 |
| wsc domain-containing protein 2 | A_05_P443422 | 1.453 | 9.65E-03 |
| eukaryotic translation initiation factor 3 subunit e | A_05_P439237 | 1.451 | 2.73E-03 |
| cytochrome c | A_05_P419817 | 1.449 | 2.00E-02 |
| histone h2a-like | A_05_P474392 | 1.445 | 1.97E-02 |
| platelet glycoprotein 4 | A_05_P270029 | 1.442 | 2.62E-02 |
| nucleolar gtp-binding protein 1 | A_05_P408677 | 1.440 | 2.46E-02 |
| proteasome subunit alpha type-6 | A_05_P436627 | 1.435 | 2.36E-02 |
| zinc finger protein 576 | A_05_P255134 | 1.435 | 1.86E-02 |
| nuclease harbi1-like^e^ | A_05_P258769 | 1.435 | 4.12E-02 |
| electron-transferring-flavoprotein dehydrogenase | A_05_P308382 | 1.433 | 4.30E-03 |
| voltage-dependent anion-selective channel protein 2 | A_05_P420122 | 1.432 | 1.37E-02 |
| ribosome production factor 2 homolog^d^ | A_05_P410877 | 1.431 | 5.60E-03 |
| proteasome subunit alpha type-5 | A_05_P409612 | 1.430 | 7.45E-03 |
| n-terminal acetyltransferase complex ard1 subunit homolog a | A_05_P408157 | 1.428 | 3.87E-02 |
| t-complex protein 1 subunit beta | A_05_P418142 | 1.426 | 2.27E-02 |
| nf-kappa-b inhibitor alpha | A_05_P422557 | 1.425 | 1.30E-02 |
| der1-like domain member 2 | A_05_P478862 | 1.420 | 4.00E-02 |
| solute carrier family 22 member 6-a | A_05_P377262 | 1.419 | 2.27E-02 |
| proteasome subunit beta type-4 | A_05_P410972 | 1.417 | 1.51E-02 |
| transcription factor 25 (basic helix-loop-helix) | A_05_P436937 | 1.415 | 4.55E-02 |
| sh3 domain-binding glutamic acid-rich-like protein 3 | A_05_P430953 | 1.413 | 2.72E-02 |
| t-complex protein 1 subunit gamma | A_05_P370762 | 1.412 | 2.04E-02 |
| lim domain binding 3 | A_05_P437592 | 1.411 | 2.19E-02 |
| iq motif and wd repeats 1 | A_05_P345082 | 1.410 | 9.06E-03 |
| aminoacyl trna synthase complex-interacting multifunctional protein 1^d^ | A_05_P407602 | 1.410 | 3.00E-02 |
| ras-related protein rab-2a | A_05_P391272 | 1.409 | 1.50E-02 |
| guanidinoacetate n-methyltransferase | A_05_P437233 | 1.408 | 1.77E-02 |
| dual specificity phosphatase 1 | A_05_P383357 | 1.406 | 2.28E-02 |
| peptidyl-prolyl cis-trans isomerase nima interacting 4 | A_05_P465787 | 1.404 | 2.50E-02 |
| smc3 protein | A_05_P385862 | 1.396 | 3.59E-03 |
| thioredoxin interacting protein | A_05_P395257 | 1.393 | 4.04E-02 |
| ccaat enhancer-binding protein delta | A_05_P431672 | 1.392 | 9.35E-03 |
| sh3 domain-containing protein 19-like | A_05_P336442 | 1.389 | 1.43E-02 |
| 60s ribosome subunit biogenesis protein nip7 homolog | A_05_P377642 | 1.386 | 4.54E-03 |
| glyceraldehyde-3-phosphate dehydrogenase | A_05_P463477 | 1.384 | 4.49E-03 |
| polyadenylate-binding protein 4 isoform 3 | A_05_P450567 | 1.380 | 3.48E-02 |
| proteolipid protein 1b | A_05_P453127 | 1.377 | 1.89E-02 |
| myh9 partial | A_05_P472392 | 1.376 | 4.18E-02 |
| zinc finger protein 490 | A_05_P403132 | 1.376 | 1.87E-02 |
| protein cappuccino homolog | A_05_P421022 | 1.374 | 3.19E-02 |
| nsfl1 cofactor p47-like | A_05_P410612 | 1.370 | 3.98E-02 |
| inosine-5 -monophosphate dehydrogenase 2 | A_05_P482917 | 1.366 | 3.24E-02 |
| arginine- mutated in early stage tumors | A_05_P367577 | 1.365 | 2.47E-02 |
| transcription factor | A_05_P308537 | 1.363 | 1.88E-02 |
| thrombospondin 4 | A_05_P300817 | 1.362 | 2.32E-02 |
| malate mitochondrial precursor | A_05_P418827 | 1.362 | 3.33E-02 |
| member ras oncogene family | A_05_P475987 | 1.358 | 3.73E-02 |
| activator of basal transcription 1 | A_05_P322402 | 1.351 | 6.10E-03 |
| protein lsm12 homolog | A_05_P455689 | 1.350 | 1.31E-02 |
| t-complex protein 1 subunit gamma | A_05_P481482 | 1.350 | 1.88E-02 |
| mature parasite-infected erythrocyte surface antigen or 2^d^ | A_05_P479972 | 1.349 | 1.77E-02 |
| negative elongation factor e | A_05_P440907 | 1.349 | 2.93E-02 |
| glutathione synthetase | A_05_P489127 | 1.349 | 4.67E-02 |
| adenylate kinase isoenzyme 6-like | A_05_P455812 | 1.348 | 1.83E-02 |
| coagulation factor x precursor | A_05_P457147 | 1.347 | 3.04E-02 |
| transcription factor 15-like | A_05_P334712 | 1.345 | 2.90E-02 |
| lim domain-containing protein 2 | A_05_P368452 | 1.340 | 1.73E-02 |
| splicing arginine serine-rich 11 | A_05_P450082 | 1.340 | 4.65E-02 |
| mediator of rna polymerase ii transcription subunit 4 | A_05_P325657 | 1.337 | 4.08E-02 |
| atp synthase subunit mitochondrial precursor | A_05_P409472 | 1.335 | 5.83E-03 |
| splicing factor 3b subunit 3 | A_05_P447182 | 1.334 | 2.84E-02 |
| heat shock protein hsp 90-alpha | A_05_P479602 | 1.332 | 4.09E-02 |
| myotubularin-related protein 2 | A_05_P476167 | 1.331 | 3.95E-02 |
| transposase homolog | A_05_P395384 | 1.331 | 4.30E-02 |
| cdc-like kinase 4 | A_05_P346932 | 1.330 | 1.38E-02 |
| 60s ribosomal protein l11 | A_05_P440812 | 1.330 | 4.52E-02 |
| hyperosmotic glycine rich protein^d^ | A_05_P484862 | 1.329 | 3.78E-02 |
| probable glutamate receptor precursor | A_05_P302932 | 1.327 | 1.49E-02 |
| high mobility group protein b2 | A_05_P253324 | 1.325 | 1.27E-02 |
| eukaryotic translation initiation factor 4e type 2 | A_05_P303322 | 1.325 | 2.27E-02 |
| actin-related protein 2 3 complex subunit 5-like protein | A_05_P482984 | 1.325 | 3.96E-02 |
| wd repeat domain 74 | A_05_P426607 | 1.324 | 1.41E-02 |
| membrane-bound transcription factor site 1 | A_05_P489444 | 1.323 | 1.60E-02 |
| ubiquinone biosynthesis protein coq7 homolog | A_05_P344462 | 1.323 | 2.01E-02 |
| 26s proteasome non-ATPase regulatory subunit 3 | A_05_P488577 | 1.321 | 4.31E-02 |
| ubiquitin c | A_05_P418397 | 1.320 | 3.25E-02 |
| mitochondrial ribosomal protein s16^d^ | A_05_P443957 | 1.319 | 3.38E-02 |
| tRNA (uracil-5-)-methyltransferase homolog^d^ | A_05_P347722 | 1.319 | 2.82E-02 |
| calcium calmodulin-dependent protein kinase iv | A_05_P455732 | 1.318 | 2.40E-02 |
| xin actin-binding repeat-containing protein 2^c^ | A_05_P430497 | 1.318 | 2.66E-02 |
| guanine nucleotide-binding protein g g g subunit gamma-13-like | A_05_P262499 | 1.316 | 3.68E-02 |
| probetacellulin precursor | A_05_P305372 | 1.315 | 2.27E-02 |
| proteasomal ubiquitin receptor adrm1-like | A_05_P410502 | 1.315 | 4.78E-02 |
| voltage-dependent anion-selective channel protein 2 | A_05_P251854 | 1.314 | 2.93E-02 |
| basic transcription factor 3 | A_05_P453997 | 1.313 | 3.50E-02 |
| cyclic amp-dependent transcription factor atf-4 | A_05_P253389 | 1.311 | 1.93E-02 |
| far upstream element-binding protein 3 | A_05_P414717 | 1.311 | 2.60E-02 |
| proteasome subunit alpha type-2 | A_05_P389557 | 1.309 | 3.47E-02 |
| nuclease harbi1-like^d^ | A_05_P455162 | 1.308 | 4.86E-02 |
| calmodulin | A_05_P377098 | 1.301 | 3.56E-02 |
| diphthamide biosynthesis protein 2 | A_05_P293207 | 1.300 | 2.35E-02 |
| apoptosis-enhancing nuclease=e | A_05_P417057 | 1.300 | 2.90E-02 |
| phosducin-like protein 3 | A_05_P430992 | 1.299 | 2.74E-02 |
| adenylate kinase 1 | A_05_P375922 | 1.298 | 2.36E-02 |
| Nocturnin | A_05_P269834 | 1.297 | 3.19E-02 |
| actin-related protein 3 | A_05_P446092 | 1.297 | 3.46E-02 |
| dermal papilla-derived protein 6 homolog | A_05_P448682 | 1.296 | 2.51E-02 |
| NADPH--cytochrome p450 reductase | A_05_P358812 | 1.295 | 3.98E-02 |
| mapk mak mrk overlapping kinase | A_05_P416647 | 1.294 | 4.95E-02 |
| Prohibitin | A_05_P414182 | 1.293 | 1.67E-02 |
| crystallin J1A^c^ | A_05_P273559 | 1.293 | 1.51E-02 |
| protoporphyrinogen oxidase | A_05_P302012 | 1.291 | 1.37E-02 |
| pleckstrin homology domain-containing family a member 7 | A_05_P301072 | 1.291 | 4.37E-02 |
| carnitine o-acetyltransferase | A_05_P272599 | 1.288 | 4.03E-02 |
| transcription initiation factor tfiid subunit 11 | A_05_P362287 | 1.287 | 3.93E-02 |
| NEFA-interacting nuclear protein NIP30^c^ | A_05_P285242 | 1.287 | 3.10E-02 |
| vitamin d receptor a | A_05_P249114 | 1.286 | 2.31E-02 |
| monoglyceride lipase | A_05_P392532 | 1.286 | 3.42E-02 |
| platelet-activating factor acetylhydrolase ib subunit alpha^d^ | A_05_P368509 | 1.281 | 3.94E-02 |
| Enolase | A_05_P407587 | 1.280 | 3.33E-02 |
| DNA-directed RNA polymerases and iii subunit rpabc1 | A_05_P257684 | 1.279 | 4.57E-02 |
| proteasome ( macropain) 26s 6^d^ | A_05_P406557 | 1.279 | 4.02E-02 |
| homeobox protein six2 | A_05_P346397 | 1.277 | 2.82E-02 |
| NEFA-interacting nuclear protein NIP30^c^ | A_05_P382382 | 1.277 | 3.94E-02 |
| intercellular adhesion molecule 1 precursor^c^ | A_05_P391022 | 1.276 | 4.23E-02 |
| von hippel-lindau binding protein 1^d^ | A_05_P255639 | 1.276 | 4.01E-02 |
| serpine1 mrna binding protein 1^d^ | A_05_P368687 | 1.275 | 2.01E-02 |
| cd209 antigen-like protein d^d^ | A_05_P418102 | 1.275 | 4.81E-02 |
| 39s ribosomal protein mitochondrial | A_05_P265274 | 1.274 | 3.79E-02 |
| cdp-diacylglycerol--serine o-phosphatidyltransferase-like^d^ | A_05_P375197 | 1.273 | 4.90E-02 |
| eukaryotic translation initiation factor 3 subunit d like | A_05_P364432 | 1.272 | 2.85E-02 |
| wd repeat-containing protein 55 | A_05_P308367 | 1.271 | 3.54E-02 |
| RNA-binding protein 4b | A_05_P388452 | 1.270 | 1.67E-02 |
| DNA-directed rna polymerase ii subunit rpb2 | A_05_P418202 | 1.270 | 4.73E-02 |
| outer dense fiber protein 3-like protein 2-like | A_05_P396722 | 1.270 | 4.50E-02 |
| selenoprotein s | A_05_P276549 | 1.270 | 4.49E-02 |
| zinc finger protein | A_05_P389667 | 1.269 | 1.87E-02 |
| mannosyl-oligosaccharide glucosidase-like^d^ | A_05_P396632 | 1.268 | 4.42E-02 |
| graves disease carrier | A_05_P368732 | 1.267 | 3.35E-02 |
| small ubiquitin-related modifier 3 precursor | A_05_P418747 | 1.265 | 2.91E-02 |
| eukaryotic translation initiation factor 2 subunit 3 | A_05_P422947 | 1.264 | 1.99E-02 |
| fast myotomal muscle tropomyosin | A_05_P464747 | 1.263 | 3.19E-02 |
| 5-aminolevulinate mitochondrial | A_05_P467616 | 1.262 | 4.84E-02 |
| NADH dehydrogenase subunit 6^c^ | A_05_P453417 | 1.260 | 2.65E-02 |
| 26s protease regulatory subunit 8 | A_05_P252159 | 1.260 | 3.39E-02 |
| ubiquitin-conjugating enzyme e2 k | A_05_P420187 | 1.260 | 4.44E-02 |
| methionine aminopeptidase 2 | A_05_P453492 | 1.259 | 3.03E-02 |
| RNA-binding protein 45 | A_05_P384167 | 1.258 | 3.73E-02 |
| cleavage and polyadenylation specificity factor subunit 1 | A_05_P347667 | 1.258 | 3.57E-02 |
| musculoskeletal embryonic nuclear protein 1^c^ | A_05_P252374 | 1.258 | 4.18E-02 |
| mediator of RNA polymerase ii transcription subunit 12 | A_05_P332977 | 1.258 | 4.95E-02 |
| small glutamine-rich tetratricopeptide repeat-containing protein a | A_05_P369727 | 1.257 | 3.17E-02 |
| ubiquitin-like protein fubi | A_05_P251364 | 1.257 | 3.78E-02 |
| guanine nucleotide-binding protein subunit beta-2-like 1 | A_05_P434467 | 1.257 | 3.70E-02 |
| coiled-coil domain-containing protein 65^c^ | A_05_P327832 | 1.256 | 3.96E-02 |
| 2,4-dienoyl-CoA reductase, mitochondrial precursor^c^ | A_05_P301357 | 1.255 | 4.96E-02 |
| leucine-rich repeat-containing protein 2^c^ | A_05_P398457 | 1.253 | 3.68E-02 |
| regulator of g-protein signaling 14 | A_05_P309402 | 1.252 | 2.48E-02 |
| capping protein (actin filament) muscle z- beta | A_05_P410762 | 1.251 | 4.63E-02 |
| dtw domain-containing protein 2-like | A_05_P491457 | 1.248 | 3.37E-02 |
| GTP-binding protein 1 | A_05_P488657 | 1.247 | 2.91E-02 |
| DNA damage-binding protein 1 | A_05_P453112 | 1.247 | 4.30E-02 |
| RNA-binding protein 5 | A_05_P458607 | 1.247 | 3.49E-02 |
| AUTS2-like protein^c^ | A_05_P465562 | 1.247 | 4.13E-02 |
| thymidine phosphorylase | A_05_P411652 | 1.246 | 3.92E-02 |
| ataxin 2-binding protein 1-like^d^ | A_05_P492062 | 1.246 | 4.73E-02 |
| mediator of rna polymerase ii transcription subunit 11 | A_05_P265464 | 1.242 | 3.78E-02 |
| splicing factor 3b subunit 1 | A_05_P421097 | 1.241 | 4.91E-02 |
| protein lin-37 homolog | A_05_P473082 | 1.239 | 4.20E-02 |
| mitochondrial ribosomal protein l28 | A_05_P265014 | 1.238 | 3.48E-02 |
| phosphatidylinositol-4-phosphate 5- type beta | A_05_P319407 | 1.238 | 4.99E-02 |
| g protein beta subunit-like^d^ | A_05_P303962 | 1.234 | 3.19E-02 |
| lamina-associated polypeptide 2 isoform beta | A_05_P372652 | 1.230 | 3.75E-02 |
| zinc-binding alcohol dehydrogenase domain-containing protein 2 | A_05_P258989 | 1.227 | 4.75E-02 |
| death-associated protein kinase 2 | A_05_P378897 | 1.224 | 4.99E-02 |
| serine threonine-protein kinase mst4 | A_05_P437777 | 1.222 | 4.70E-02 |
| tho complex subunit 5 homolog | A_05_P423802 | 1.216 | 4.69E-02 |
| beta-galactoside-binding lectin | A_05_P441152 | 1.215 | 4.74E-02 |
| protein fat-free homolog^d^ | A_05_P372287 | 1.211 | 4.94E-02 |
| interferon-induced helicase c domain-containing protein 1 | A_05_P486287 | 1.205 | 4.53E-02 |
| ***Dec Fish*** |  |  |  |
| rRNA promoter binding protein^c^ | A_05_P425972 | 5.474 | 7.07E-03 |
| myosin light chain 3-like | A_05_P248844 | 4.690 | 1.29E-03 |
| myosin heavy chain | A_05_P277167 | 4.167 | 3.87E-03 |
| rRNA promoter binding protein^c,^ | A_05_P393707 | 3.572 | 9.75E-04 |
| tropomyosin 2^d^ | A_05_P488277 | 3.453 | 2.01E-02 |
| troponin T, cardiac muscle^c^ | A_05_P255254 | 3.386 | 1.04E-02 |
| troponin slow skeletal muscle | A_05_P277927 | 3.332 | 7.86E-03 |
| tropomyosin 3 | A_05_P377692 | 3.075 | 1.33E-02 |
| parvalbumin 2 | A_05_P423182 | 3.032 | 3.13E-02 |
| parvalbumin beta-2 | A_05_P429727 | 2.982 | 1.82E-02 |
| eukaryotic translation elongation factor 1 epsilon 1 | A_05_P468757 | 2.899 | 4.73E-03 |
| tropomyosin partial | A_05_P336882 | 2.894 | 7.12E-03 |
| senescence-associated protein^d^ | A_05_P262709 | 2.819 | 1.64E-03 |
| myosin binding protein cardiac | A_05_P277492 | 2.688 | 3.53E-03 |
| keratin-associated protein 10-4^c^ | A_05_P485782 | 2.441 | 3.35E-03 |
| formiminotransferase cyclodeaminase | A_05_P413832 | 2.344 | 1.85E-02 |
| troponin slow skeletal muscle-like | A_05_P353137 | 2.340 | 2.11E-02 |
| protein cln8 | A_05_P374957 | 2.340 | 1.42E-02 |
| serine hydroxymethyltransferase 2 | A_05_P305947 | 2.319 | 1.74E-02 |
| zinc finger protein 22 | A_05_P399592 | 2.242 | 4.27E-02 |
| signal peptide peptidase-like 2b | A_05_P375042 | 2.239 | 1.45E-02 |
| novel protein^d^ | A_05_P297287 | 2.219 | 2.28E-02 |
| glyceraldehyde-3-phosphate dehydrogenase | A_05_P419277 | 2.200 | 1.09E-02 |
| det1- and ddb1-associated protein 1 | A_05_P372807 | 2.182 | 4.26E-02 |
| lymphatic vessel endothelial hyaluronic acid receptor 1 precursor | A_05_P391972 | 2.159 | 3.14E-02 |
| actinin alpha 2 | A_05_P352192 | 2.155 | 2.40E-03 |
| det1- and ddb1-associated protein 1 | A_05_P450312 | 2.127 | 2.94E-02 |
| 60 kda heat shock mitochondrial | A_05_P424877 | 2.084 | 3.52E-02 |
| rab3 GTPase-activating protein non catalytic subunit | A_05_P315167 | 2.015 | 5.90E-03 |
| ribosomal protein s15a | A_05_P398402 | 1.996 | 3.99E-02 |
| hemagglutinin invasin | A_05_P451112 | 1.969 | 2.81E-02 |
| polq-like^d^ | A_05_P268299 | 1.930 | 4.88E-05 |
| 40S ribosomal protein S27^c^ | A_05_P390192 | 1.925 | 1.63E-02 |
| protein cappuccino homolog | A_05_P421022 | 1.923 | 2.44E-02 |
| middle subunit | A_05_P392927 | 1.921 | 1.28E-02 |
| eukaryotic translation initiation factor 5b | A_05_P420767 | 1.898 | 3.04E-02 |
| camp responsive element binding 2 | A_05_P327372 | 1.889 | 3.89E-03 |
| lon protease mitochondrial-like | A_05_P294807 | 1.878 | 6.92E-03 |
| proline dehydrogenase 1 | A_05_P480672 | 1.868 | 4.12E-02 |
| HEAT repeat-containing protein KIAA1833^c^ | A_05_P451422 | 1.865 | 1.42E-02 |
| splicing factor 3a subunit 1 | A_05_P347292 | 1.855 | 2.16E-02 |
| protein-l-isoaspartate(d-aspartate) o-methyltransferase-like | A_05_P488617 | 1.852 | 2.09E-02 |
| rcc2 homolog | A_05_P337597 | 1.849 | 1.48E-03 |
| nitrilase homolog 1 | A_05_P292827 | 1.849 | 2.92E-03 |
| oxoglutarate (alpha-ketoglutarate) dehydrogenase | A_05_P306767 | 1.843 | 4.27E-02 |
| bifunctional methylenetetrahydrofolate dehydrogenase mitochondrial precursor | A_05_P437152 | 1.838 | 2.41E-02 |
| serine threonine-protein kinase mst4 | A_05_P374852 | 1.832 | 2.36E-02 |
| cytochrome c oxidase subunit vib isoform 1 | A_05_P406022 | 1.829 | 7.14E-03 |
| lily-type lectin^d^ | A_05_P355002 | 1.821 | 4.69E-03 |
| Myoglobin | A_05_P252139 | 1.816 | 3.77E-02 |
| RNA-binding protein fus^d^ | A_05_P306342 | 1.791 | 1.94E-02 |
| beta- -n-acetylglucosaminyltransferase lunatic fringe-like | A_05_P455052 | 1.784 | 1.16E-02 |
| complement C1q-like protein 2 precursor^c^ | A_05_P435182 | 1.770 | 1.75E-02 |
| snrna-activating protein complex subunit 1 | A_05_P385407 | 1.770 | 3.46E-02 |
| nucleoside diphosphate kinase a | A_05_P376177 | 1.765 | 4.53E-02 |
| ras homolog gene member q | A_05_P346977 | 1.758 | 1.37E-02 |
| adrenomedullin 5 | A_05_P315072 | 1.757 | 3.71E-02 |
| member of ras oncogene family | A_05_P257444 | 1.754 | 4.78E-02 |
| transposable element tcb2 transposase | A_05_P432747 | 1.754 | 4.10E-02 |
| distal-less homeobox 3 | A_05_P402292 | 1.752 | 2.30E-02 |
| general transcription factor 3c polypeptide 5 | A_05_P328252 | 1.743 | 3.21E-02 |
| stonustoxin subunit beta^c^ | A_05_P369457 | 1.732 | 2.59E-02 |
| peroxisome proliferator-activated receptor beta | A_05_P486222 | 1.732 | 1.75E-02 |
| anaphase-promoting complex subunit 1 | A_05_P486122 | 1.724 | 1.94E-02 |
| f-actin-capping protein subunit beta | A_05_P370552 | 1.724 | 3.94E-02 |
| protein transport protein Sec31A^c^ | A_05_P375907 | 1.718 | 6.77E-03 |
| protein | A_05_P297762 | 1.712 | 7.21E-04 |
| apoptotic protease-activating factor 1 isoform 2 | A_05_P311582 | 1.711 | 1.08E-02 |
| oocyte zinc finger protein 6-like^d^ | A_05_P411372 | 1.710 | 7.11E-03 |
| late secretory pathway protein avl9 homolog^d^ | A_05_P482597 | 1.699 | 3.84E-02 |
| protein dgcr14-like | A_05_P320347 | 1.698 | 7.55E-03 |
| WD repeat-containing protein 21A^c^ | A_05_P292092 | 1.692 | 2.32E-02 |
| fructose-bisphosphate aldolase a | A_05_P394817 | 1.689 | 1.32E-02 |
| RNA-binding protein 45 | A_05_P441127 | 1.688 | 2.24E-02 |
| 4-aminobutyrate aminotransferase | A_05_P372142 | 1.681 | 5.44E-03 |
| soluble guanylyl cyclase beta-3 | A_05_P307497 | 1.679 | 4.09E-02 |
| laminin subunit alpha-3 isoform 3 | A_05_P491367 | 1.675 | 1.31E-03 |
| ribosomal protein l13 | A_05_P361627 | 1.672 | 3.18E-02 |
| der1-like domain member 2 | A_05_P478862 | 1.672 | 4.72E-02 |
| zinc finger bed domain-containing protein 1-like | A_05_P483207 | 1.668 | 9.38E-03 |
| cell division cycle and apoptosis regulator protein 1 | A_05_P294202 | 1.668 | 3.50E-02 |
| gastrula zinc finger^d^ | A_05_P398927 | 1.662 | 2.87E-02 |
| glycogen debranching enzyme | A_05_P447892 | 1.660 | 2.96E-02 |
| eps8-like 1 | A_05_P342252 | 1.660 | 1.73E-02 |
| proline-rich nuclear receptor coactivator 2 | A_05_P470787 | 1.654 | 1.15E-02 |
| ribosomal protein l19 | A_05_P432907 | 1.654 | 3.21E-02 |
| zinc finger c3h1 domain-containing protein^d^ | A_05_P457937 | 1.652 | 1.12E-02 |
| transitional endoplasmic reticulum atpase | A_05_P259499 | 1.652 | 3.29E-02 |
| DNA (cytosine-5)-methyltransferase 1 | A_05_P468507 | 1.650 | 3.34E-02 |
| filamin-c isoform 3 | A_05_P386422 | 1.648 | 2.37E-02 |
| coiled-coil domain-containing protein 84^c^ | A_05_P325672 | 1.648 | 4.31E-02 |
| non-LTR (long terminal repeat) retrotransposon and non-LTR retrovirus reverse transcriptase (RT)^c^ | A_05_P448569 | 1.643 | 3.42E-02 |
| 60 kda heat shock mitochondrial | A_05_P257429 | 1.642 | 4.06E-03 |
| dym protein | A_05_P267324 | 1.639 | 1.97E-03 |
| b-cell translocation gene anti-proliferative | A_05_P278457 | 1.635 | 1.19E-02 |
| peptidylprolyl isomerase -like 2 | A_05_P355237 | 1.632 | 9.58E-03 |
| myelin protein zero | A_05_P393932 | 1.632 | 1.50E-02 |
| cardiolipin synthase-like | A_05_P319947 | 1.632 | 3.48E-02 |
| solute carrier family 35 member e1 | A_05_P322192 | 1.627 | 3.14E-02 |
| ring finger protein 128 | A_05_P450607 | 1.623 | 2.47E-02 |
| protein arginine methyltransferase 3 | A_05_P294392 | 1.617 | 8.81E-03 |
| phosphatase and tensin-like protein^d^ | A_05_P368412 | 1.615 | 1.98E-02 |
| orf2-encoded protein^d^ | A_05_P487487 | 1.615 | 4.99E-02 |
| probable RNA-directed DNA polymerase from transposon BS^c^ | A_05_P469397 | 1.614 | 4.54E-02 |
| titin^c^ | A_05_P354062 | 1.612 | 3.14E-02 |
| v-maf musculoaponeurotic fibrosarcoma oncogene-like | A_05_P459727 | 1.612 | 4.77E-02 |
| signal peptide peptidase-like 2a-like | A_05_P477012 | 1.610 | 2.01E-02 |
| serine threonine-protein kinase nek6-like | A_05_P257584 | 1.610 | 1.65E-02 |
| transposable element tcb1 transposase | A_05_P491342 | 1.609 | 3.82E-02 |
| friend leukemia integration 1 transcription factor | A_05_P369227 | 1.608 | 3.79E-02 |
| interleukin 17d | A_05_P346642 | 1.607 | 4.74E-02 |
| nuclear transcription factor y subunit beta | A_05_P332347 | 1.606 | 4.68E-02 |
| CD209 antigen-like protein D^c^ | A_05_P290987 | 1.605 | 3.14E-02 |
| riboflavin transporter 2-like^d^ | A_05_P317197 | 1.604 | 4.47E-02 |
| RNA-binding protein 5 | A_05_P391362 | 1.603 | 4.47E-02 |
| transposable element tcb1 transposase^d^ | A_05_P479967 | 1.603 | 3.16E-02 |
| opioid-binding protein cell adhesion molecule-like | A_05_P347602 | 1.600 | 2.68E-02 |
| snare-associated protein snapin | A_05_P314107 | 1.595 | 2.86E-02 |
| anterior gradient protein 3 homolog precursor | A_05_P357117 | 1.590 | 2.37E-02 |
| troponin I, fast skeletal muscle^c^ | A_05_P432892 | 1.589 | 4.67E-02 |
| transmembrane protein 198-b-like | A_05_P329512 | 1.587 | 1.18E-02 |
| novel protein cytochrome family subfamily j^d^ | A_05_P303502 | 1.587 | 2.40E-02 |
| leukocyte elastase inhibitor | A_05_P290237 | 1.580 | 2.50E-02 |
| proteinase-activated receptor 1-like | A_05_P339627 | 1.579 | 4.19E-02 |
| FGF2 [*Salmo salar*] | A_05_P494417 | 1.577 | 7.50E-03 |
| gem (nuclear organelle) associated protein 8 | A_05_P441267 | 1.577 | 4.76E-02 |
| glutamine synthetase | A_05_P278117 | 1.577 | 4.01E-02 |
| ccaat enhancer-binding protein zeta | A_05_P384642 | 1.576 | 2.57E-02 |
| swi snf matrix actin dependent regulator of subfamily member 1 | A_05_P391157 | 1.572 | 2.74E-02 |
| calmodulin | A_05_P377098 | 1.572 | 4.55E-02 |
| 3 (2 ) -bisphosphate nucleotidase 1-like | A_05_P462572 | 1.572 | 2.83E-02 |
| ADP-ribosylation factor 1 | A_05_P261274 | 1.572 | 4.83E-02 |
| 2 -cyclic-nucleotide 3 -phosphodiesterase | A_05_P392677 | 1.572 | 5.00E-02 |
| mapk mak mrk overlapping kinase | A_05_P416647 | 1.570 | 1.62E-03 |
| plectin isoform 1hij | A_05_P287812 | 1.569 | 5.01E-03 |
| inactive dipeptidyl peptidase 10 | A_05_P479097 | 1.568 | 3.23E-02 |
| pleckstrin homology domain-containing family g member 4b-like | A_05_P486407 | 1.568 | 2.92E-02 |
| chromosome 7 open reading frame 30 | A_05_P259644 | 1.567 | 6.72E-03 |
| peptidase (mitochondrial processing) alpha | A_05_P378807 | 1.566 | 2.40E-02 |
| pla2g15 protein^d^ | A_05_P453667 | 1.566 | 2.01E-02 |
| herpes virus major outer envelope glycoprotein (BLLF1)^c^ | A_05_P416597 | 1.564 | 8.16E-03 |
| septin 2 | A_05_P437982 | 1.560 | 1.08E-03 |
| zinc metalloproteinase c | A_05_P334262 | 1.557 | 1.42E-02 |
| novel protein e-cadherin^d^ | A_05_P332787 | 1.556 | 2.30E-02 |
| rho gtpase-activating protein 27 | A_05_P450792 | 1.554 | 2.21E-02 |
| ADP/ATP translocase 2 | A_05_P421709 | 1.553 | 2.28E-02 |
| regulator of g-protein signaling 5 | A_05_P412337 | 1.553 | 4.94E-02 |
| serine threonine-protein kinase nek9 | A_05_P331252 | 1.548 | 1.09E-03 |
| insulin-like growth factor i receptor | A_05_P249834 | 1.548 | 3.32E-02 |
| cell division cycle 42 (gtp binding 25kda) | A_05_P394867 | 1.547 | 3.71E-02 |
| nucleic acid binding protein | A_05_P388433 | 1.545 | 3.53E-02 |
| transposable element tcb1 transposase | A_05_P483047 | 1.545 | 2.72E-02 |
| serologically defined colon cancer antigen 1 homolog | A_05_P379882 | 1.544 | 2.48E-02 |
| stromal cell-derived factor 2-like 1 | A_05_P267349 | 1.544 | 3.45E-02 |
| sfrs11 partial | A_05_P260054 | 1.543 | 2.48E-02 |
| anthrax toxin receptor 1-like | A_05_P401197 | 1.543 | 4.97E-02 |
| transcription factor sox-17 | A_05_P341482 | 1.541 | 5.89E-03 |
| ataxin 2-binding protein 1-like^d^ | A_05_P342597 | 1.541 | 3.70E-02 |
| transcription termination factor Rho^c^ | A_05_P466552 | 1.540 | 4.98E-02 |
| polymerase delta-interacting protein 2 | A_05_P380842 | 1.535 | 3.85E-02 |
| lymphoid-restricted membrane protein^c^ | A_05_P392017 | 1.535 | 3.50E-02 |
| fk506-binding protein 1a | A_05_P381982 | 1.531 | 2.50E-02 |
| si:ch211-147a11.3^c^ | A_05_P443717 | 1.530 | 4.39E-03 |
| insulin gene enhancer protein isl-1 | A_05_P489328 | 1.530 | 4.94E-02 |
| spidroin-2^c^ | A_05_P440387 | 1.529 | 4.58E-02 |
| cdc45 cell division cycle 45-like ( cerevisiae) | A_05_P304202 | 1.528 | 3.08E-02 |
| sam domain-containing protein samsn-1 | A_05_P318082 | 1.528 | 2.81E-02 |
| activating signal cointegrator 1 | A_05_P491347 | 1.526 | 3.44E-02 |
| probable peptidyl-trna hydrolase 2-like | A_05_P305592 | 1.524 | 3.78E-03 |
| solute carrier family 22 member 17 | A_05_P330097 | 1.523 | 1.89E-02 |
| transposable element tcb1 transposase | A_05_P389052 | 1.523 | 4.90E-02 |
| transposable element tcb1 transposase | A_05_P435707 | 1.523 | 3.46E-02 |
| esophageal cancer-related gene 4 protein precursor | A_05_P278247 | 1.521 | 1.86E-02 |
| filamin A-interacting protein 1-like^c^ | A_05_P286067 | 1.521 | 4.56E-02 |
| loc100007612 protein^d^ | A_05_P448092 | 1.520 | 1.30E-02 |
| tumor necrosis factor receptor superfamily member 14 precursor | A_05_P314437 | 1.520 | 4.67E-02 |
| ATP-binding cassette sub-family f member 3-like | A_05_P260179 | 1.519 | 1.84E-02 |
| solute carrier family member 5 | A_05_P370427 | 1.518 | 1.09E-02 |
| dym protein | A_05_P458847 | 1.518 | 2.83E-02 |
| Transposase | A_05_P334786 | 1.518 | 3.08E-02 |
| protein arginine methyltransferase 5 | A_05_P307472 | 1.518 | 3.75E-02 |
| HEAT repeat-containing protein 3^c^ | A_05_P465312 | 1.517 | 1.46E-02 |
| transcription factor | A_05_P490902 | 1.516 | 7.85E-03 |
| matrix metalloproteinase 9 | A_05_P280437 | 1.515 | 2.79E-02 |
| splicing factor u2af 26 kda subunit | A_05_P396177 | 1.514 | 3.60E-02 |
| eukaryotic translation initiation factor subunit d | A_05_P312572 | 1.514 | 4.15E-02 |
| telomerase reverse transcriptase | A_05_P456152 | 1.513 | 1.18E-02 |
| dynamin 1 | A_05_P400202 | 1.513 | 2.97E-02 |
| peptide methionine sulfoxide reductase | A_05_P450442 | 1.512 | 3.78E-02 |
| otu domain containing 5 | A_05_P262019 | 1.511 | 3.38E-02 |
| cchain crystal structure of the moesin ferm domain complex | A_05_P431812 | 1.510 | 1.61E-02 |
| WD repeat-containing protein 24^c^ | A_05_P430097 | 1.508 | 5.86E-03 |
| interferon-induced guanylate-binding protein 1 | A_05_P486782 | 1.505 | 6.20E-03 |
| adenosylhomocysteinase 3 | A_05_P370477 | 1.505 | 5.90E-03 |
| ADP-ribosylation factor 1 | A_05_P377937 | 1.504 | 3.34E-02 |
| dnaj homolog subfamily b member 6 | A_05_P454877 | 1.503 | 1.29E-02 |
| antizyme inhibitor 1 | A_05_P320487 | 1.502 | 1.88E-02 |
| hemoglobin subunit beta^c^ | A_05_P395622 | 1.498 | 1.42E-02 |
| solute carrier family 35 member b1 | A_05_P432152 | 1.497 | 3.21E-02 |
| myosin ic | A_05_P330217 | 1.497 | 2.20E-02 |
| histone-lysine n- h3 lysine-9 specific 3 | A_05_P421082 | 1.493 | 2.32E-02 |
| sry-box containing gene 1a | A_05_P305597 | 1.493 | 2.83E-02 |
| zinc finger bed domain-containing protein 1-like | A_05_P434247 | 1.490 | 2.03E-02 |
| mitochondrial import receptor subunit tom40 homolog | A_05_P424447 | 1.490 | 2.29E-02 |
| ap-2 complex subunit mu | A_05_P317747 | 1.490 | 4.28E-02 |
| structural maintenance of chromosomes protein 1a | A_05_P493055 | 1.489 | 8.74E-03 |
| phosphoglycerate mutase 1 | A_05_P435247 | 1.489 | 2.62E-02 |
| transient receptor potential cation subfamily member 7 | A_05_P382862 | 1.487 | 2.37E-03 |
| protein s100-a5-like^d^ | A_05_P354107 | 1.487 | 2.20E-02 |
| nucleoporin seh1 isoform 2 | A_05_P258344 | 1.485 | 1.73E-02 |
| transcription termination factor Rho; provisional^c^ | A_05_P399112 | 1.485 | 9.71E-03 |
| HIV TAT specific factor 1 | A_05_P273339 | 1.484 | 2.34E-02 |
| Methyltransferase kiaa1456-like | A_05_P331002 | 1.484 | 3.63E-02 |
| vomeronasal type-2 receptor 1-like | A_05_P461677 | 1.483 | 4.84E-02 |
| ATP-binding cassette sub-family a member 2 | A_05_P317612 | 1.482 | 4.46E-02 |
| nop2 sun domain member 5 | A_05_P440947 | 1.482 | 3.44E-02 |
| paired box protein pax-1 | A_05_P353807 | 1.481 | 2.46E-02 |
| snail homolog sna | A_05_P310067 | 1.481 | 4.02E-02 |
| mitotic spindle assembly checkpoint protein mad2a | A_05_P449377 | 1.475 | 6.63E-03 |
| transposable element tcb1 transposase | A_05_P389042 | 1.475 | 4.61E-02 |
| surp and g-patch domain-containing protein 1-like^d^ | A_05_P374417 | 1.474 | 3.14E-02 |
| integrin-linked kinase-associated serine threonine phosphatase 2c | A_05_P331542 | 1.469 | 3.42E-02 |
| rab11 family-interacting protein 2 | A_05_P344947 | 1.467 | 4.95E-02 |
| uncharacterized zinc finger protein 814-like^d^ | A_05_P395527 | 1.465 | 3.14E-03 |
| mitochondrial ribosomal protein l11 | A_05_P264434 | 1.463 | 3.51E-02 |
| OX-2 membrane glycoprotein precursor^c^ | A_05_P441047 | 1.462 | 1.83E-02 |
| myelin basic protein | A_05_P368497 | 1.462 | 4.10E-02 |
| 39s ribosomal protein mitochondrial precursor^d^ | A_05_P483092 | 1.462 | 3.06E-02 |
| protein rcc2 | A_05_P261909 | 1.461 | 2.69E-02 |
| e3 ubiquitin-protein ligase rnf14 | A_05_P424817 | 1.456 | 3.42E-02 |
| nucleolysin tia-1 isoform p40 isoform 2 | A_05_P321132 | 1.455 | 9.92E-03 |
| HERV-H LTR-associating protein 2 precursor^c^ | A_05_P357817 | 1.455 | 4.47E-02 |
| uncharacterized protein MGC35361^c^ | A_05_P383322 | 1.453 | 3.51E-03 |
| cdp-diacylglycerol--serine o-phosphatidyltransferase-like^d^ | A_05_P375197 | 1.453 | 8.54E-03 |
| udp- c:betagal beta- -n-acetylglucosaminyltransferase 5 | A_05_P437782 | 1.453 | 3.76E-02 |
| transposase homolog | A_05_P401652 | 1.453 | 1.34E-02 |
| transposase homolog | A_05_P388127 | 1.452 | 2.24E-02 |
| solute carrier family 35 member b1 | A_05_P448242 | 1.452 | 3.72E-02 |
| glutamine-rich protein 1^c^ | A_05_P323127 | 1.451 | 2.34E-02 |
| microtubule-associated protein 1 light chain 3 beta | A_05_P414062 | 1.451 | 3.70E-02 |
| major facilitator superfamily domain-containing protein 1 | A_05_P274359 | 1.448 | 2.22E-02 |
| transposable element tcb1 transposase | A_05_P469462 | 1.448 | 1.28E-02 |
| eukaryotic translation initiation factor 3 subunit j | A_05_P261124 | 1.447 | 3.28E-02 |
| probable lysosomal cobalamin transporter-like^d^ | A_05_P375862 | 1.447 | 3.59E-02 |
| dolichyl-phosphate beta-glucosyltransferase | A_05_P272994 | 1.446 | 2.52E-02 |
| zinc finger BED domain-containing protein 5^c^ | A_05_P365917 | 1.446 | 2.90E-02 |
| splicing arginine serine-rich 2 | A_05_P371507 | 1.446 | 3.06E-02 |
| NAD-dependent deacetylase sirtuin-7 | A_05_P458897 | 1.445 | 3.27E-02 |
| legumain | A_05_P275684 | 1.444 | 4.00E-03 |
| transmembrane protein 165 | A_05_P344892 | 1.444 | 2.65E-02 |
| interferon-induced guanylate-binding protein 1 | A_05_P379977 | 1.443 | 9.16E-03 |
| protein argonaute-3^d^ | A_05_P476512 | 1.441 | 4.66E-03 |
| LINE-1 reverse transcriptase homolog^c^ | A_05_P325222 | 1.441 | 3.07E-02 |
| fish virus induced trim protein^d^ | A_05_P470762 | 1.439 | 1.63E-02 |
| mixed lineage kinase domain-like | A_05_P424592 | 1.439 | 1.80E-02 |
| dexamethasone-induced ras-related protein 1-like | A_05_P410782 | 1.439 | 1.70E-02 |
| gem-associated protein 5 | A_05_P494697 | 1.438 | 3.35E-02 |
| 60s ribosomal protein l17 | A_05_P482232 | 1.437 | 2.13E-02 |
| loc734178 protein^d^ | A_05_P344677 | 1.436 | 2.03E-02 |
| origin recognition subunit 3-like | A_05_P473122 | 1.436 | 4.33E-02 |
| myoblast determination protein 2 | A_05_P249699 | 1.435 | 2.06E-02 |
| Deoxyribonuclease gamma precursor | A_05_P443902 | 1.435 | 4.60E-02 |
| leukotriene b4 receptor | A_05_P434107 | 1.434 | 1.96E-02 |
| rho gtpase-activating protein 20 | A_05_P413097 | 1.434 | 2.49E-02 |
| claudin 7^d^ | A_05_P364717 | 1.431 | 6.73E-03 |
| selenium-binding protein 1 | A_05_P459452 | 1.430 | 3.66E-02 |
| sarcosine mitochondrial | A_05_P426427 | 1.429 | 4.11E-02 |
| dihydrolipoyllysine-residue succinyltransferase component of 2-oxoglutarate dehydrogenase mitochondrial | A_05_P407852 | 1.428 | 2.28E-02 |
| eukaryotic translation initiation factor 4 gamma 1 | A_05_P374942 | 1.428 | 2.94E-02 |
| neurogenic locus notch homolog protein 1 | A_05_P312082 | 1.427 | 3.13E-02 |
| ubiquitin-like modifier activating enzyme 1 | A_05_P400067 | 1.426 | 3.70E-03 |
| arrestin domain containing 2 | A_05_P377207 | 1.426 | 1.70E-02 |
| transmembrane protein 43 | A_05_P251459 | 1.426 | 2.26E-02 |
| differentially expressed in fdcp 6 homolog | A_05_P284227 | 1.424 | 4.11E-03 |
| g-protein coupled receptor 182 | A_05_P413177 | 1.424 | 5.20E-03 |
| scy1-like 3 ( cerevisiae)^d^ | A_05_P481847 | 1.423 | 2.36E-02 |
| deoxyribonuclease gamma precursor | A_05_P494027 | 1.421 | 1.54E-02 |
| tp53-regulating kinase | A_05_P378322 | 1.420 | 4.27E-02 |
| oxysterol-binding protein 2-like | A_05_P485247 | 1.418 | 4.37E-02 |
| recoverin-like | A_05_P272204 | 1.417 | 1.64E-02 |
| rho gtpase activating protein 9 | A_05_P438802 | 1.417 | 2.21E-02 |
| solute carrier family 25 member 40 | A_05_P423242 | 1.416 | 4.65E-02 |
| casein kinase gamma 2 | A_05_P378027 | 1.415 | 1.07E-02 |
| novel protein (zgc:110579)^d^ | A_05_P297502 | 1.415 | 4.56E-02 |
| homolog subfamily b member 6 | A_05_P387667 | 1.414 | 3.65E-02 |
| b-cell lymphoma 6 protein | A_05_P316607 | 1.412 | 2.50E-02 |
| transmembrane and tpr repeat-containing protein 4 | A_05_P388932 | 1.410 | 1.19E-02 |
| nucleoporin seh1 isoform 2 | A_05_P447997 | 1.410 | 2.92E-02 |
| serine threonine-protein phosphatase 2a catalytic subunit alpha isoform | A_05_P448577 | 1.409 | 8.61E-03 |
| glutamate dehydrogenase | A_05_P249224 | 1.409 | 2.56E-02 |
| guanine nucleotide-binding 3 | A_05_P473337 | 1.409 | 2.48E-02 |
| serine threonine-protein kinase plk2 | A_05_P383012 | 1.409 | 2.19E-02 |
| sentrin-specific protease 2 | A_05_P381032 | 1.409 | 4.63E-02 |
| myosin heavy chain, fast skeletal muscle^c^ | A_05_P481967 | 1.409 | 4.21E-02 |
| arf-gap with sh3 ank repeat and ph domain-containing protein 3^d^ | A_05_P386347 | 1.408 | 3.60E-02 |
| RNA-directed dna polymerase from mobile element jockey-like | A_05_P404707 | 1.407 | 3.58E-02 |
| transposable element tcb1 transposase | A_05_P486137 | 1.406 | 1.02E-02 |
| complement c1q-like protein 4 precursor^d^ | A_05_P388222 | 1.406 | 1.36E-02 |
| heat shock protein beta-8 | A_05_P348612 | 1.406 | 3.98E-02 |
| thrombospondin 2 | A_05_P322687 | 1.405 | 1.72E-02 |
| iq motif containing gtpase activating protein 1 | A_05_P321622 | 1.404 | 2.90E-02 |
| bromodomain-containing protein 4 | A_05_P473657 | 1.403 | 4.07E-02 |
| prostaglandin reductase 1-like^d^ | A_05_P372057 | 1.402 | 3.62E-02 |
| zinc mym-type 4 | A_05_P289627 | 1.399 | 8.36E-03 |
| lysosome-associated membrane glycoprotein 2 precursor | A_05_P438677 | 1.398 | 1.00E-02 |
| membrane-bound transcription factor site 1 | A_05_P489444 | 1.397 | 9.99E-03 |
| protein kinase c theta type | A_05_P291572 | 1.397 | 3.05E-02 |
| multiple inositol polyphosphate phosphatase 1-like | A_05_P296712 | 1.396 | 1.59E-02 |
| zinc finger protein 135-like^d^ | A_05_P395532 | 1.396 | 2.53E-02 |
| protein tbrg4 | A_05_P283682 | 1.395 | 1.16E-02 |
| beta-hexosaminidase subunit alpha precursor | A_05_P274319 | 1.395 | 4.70E-02 |
| hemagglutinin/amebocyte aggregation factor precursor^c^ | A_05_P439102 | 1.395 | 3.98E-02 |
| bifunctional protein ncoat isoform 2 | A_05_P480287 | 1.394 | 2.72E-02 |
| interferon inducible mx protein | A_05_P248684 | 1.392 | 1.45E-02 |
| protein kinase c binding protein 1 | A_05_P398232 | 1.392 | 3.98E-02 |
| mannose-p-dolichol utilization defect 1 | A_05_P376442 | 1.392 | 2.86E-02 |
| lambda-crystallin homolog | A_05_P274089 | 1.389 | 9.50E-03 |
| coiled-coil domain-containing protein 56 | A_05_P268819 | 1.388 | 1.43E-02 |
| period homolog 1 | A_05_P462872 | 1.387 | 6.29E-03 |
| transposase | A_05_P483949 | 1.387 | 8.51E-03 |
| homeobox-containing protein 1-like | A_05_P490657 | 1.387 | 4.25E-02 |
| beta-mannosidase | A_05_P286677 | 1.386 | 2.47E-02 |
| protein apcdd1 | A_05_P385847 | 1.385 | 4.84E-02 |
| transposase | A_05_P254799 | 1.384 | 2.09E-02 |
| 7-dehydrocholesterol reductase | A_05_P271219 | 1.384 | 2.97E-02 |
| tyrosine-protein phosphatase non-receptor type 22 isoform 2 | A_05_P441537 | 1.384 | 3.81E-02 |
| protein osteopotentia homolog^d^ | A_05_P483477 | 1.382 | 2.40E-02 |
| myelin basic protein | A_05_P375352 | 1.382 | 1.65E-02 |
| sialic acid-binding ig-like lectin 5-like | A_05_P304307 | 1.382 | 4.25E-02 |
| netrin receptor dcc | A_05_P404687 | 1.381 | 1.05E-02 |
| zinc finger protein 703 | A_05_P266129 | 1.381 | 1.13E-02 |
| transposable element tcb1 transposase | A_05_P398395 | 1.381 | 9.76E-03 |
| tissue-type plasminogen activator | A_05_P464162 | 1.381 | 2.76E-02 |
| ccaat enhancer-binding protein zeta | A_05_P328807 | 1.379 | 2.49E-02 |
| transmembrane protein 182-like | A_05_P352887 | 1.379 | 3.41E-02 |
| probable cation-transporting atpase 13a1 | A_05_P470082 | 1.379 | 2.47E-02 |
| oocyte zinc finger protein 20 | A_05_P474562 | 1.378 | 2.30E-02 |
| 4-hydroxyphenylpyruvate dioxygenase | A_05_P363322 | 1.378 | 3.53E-02 |
| wd repeat and fyve domain containing 2 | A_05_P476217 | 1.376 | 1.75E-02 |
| dipeptidyl peptidase 9 | A_05_P369257 | 1.376 | 2.97E-02 |
| eukaryotic translation initiation factor 5a-1 | A_05_P368122 | 1.375 | 1.10E-02 |
| eukaryotic translation initiation factor 3 subunit A^c^ | A_05_P331582 | 1.375 | 2.66E-02 |
| tropomyosin alpha-3 chain isoform 2 | A_05_P266969 | 1.375 | 4.53E-02 |
| thrombomodulin precursor | A_05_P297137 | 1.375 | 4.08E-02 |
| carbonyl reductase family member 4 | A_05_P293122 | 1.373 | 2.50E-02 |
| myomesin-3^c^ | A_05_P313542 | 1.372 | 4.34E-02 |
| v-type proton atpase subunit s1-like | A_05_P425212 | 1.372 | 4.03E-02 |
| rcc2 homolog | A_05_P401557 | 1.371 | 1.70E-02 |
| ribosomal protein s18 | A_05_P364822 | 1.370 | 9.95E-03 |
| glutathione peroxidase 3 precursor | A_05_P368527 | 1.370 | 3.54E-02 |
| dual specificity testis-specific protein kinase 1-like | A_05_P330902 | 1.370 | 4.91E-02 |
| active regulator of sirt1-like^d^ | A_05_P330757 | 1.369 | 3.53E-02 |
| sodium-coupled neutral amino acid transporter 2 | A_05_P383582 | 1.368 | 4.18E-02 |
| active regulator of sirt1-like^d^ | A_05_P383403 | 1.368 | 2.21E-02 |
| zinc finger protein | A_05_P389667 | 1.367 | 9.07E-03 |
| minor histocompatibility protein ha-1^d^ | A_05_P374902 | 1.367 | 1.22E-02 |
| noggin | A_05_P345957 | 1.367 | 4.65E-02 |
| kiaa0232 gene product | A_05_P486872 | 1.365 | 1.84E-02 |
| cytochrome c oxidase polypeptide mitochondrial precursor | A_05_P366972 | 1.362 | 2.33E-02 |
| poly | A_05_P392602 | 1.361 | 4.72E-02 |
| tgf-beta-activated kinase 1 and map3k7-binding protein 2^d^ | A_05_P382267 | 1.360 | 4.14E-02 |
| probable g-protein coupled receptor 173-like | A_05_P296212 | 1.360 | 3.40E-02 |
| lymphatic vessel endothelial hyaluronic acid receptor 1 precursor | A_05_P385167 | 1.357 | 9.52E-03 |
| dual specificity testis-specific protein kinase 1-like | A_05_P391427 | 1.356 | 3.50E-02 |
| udp-n-acetylglucosamine--dolichyl-phosphate n-acetylglucosaminephosphotransferase | A_05_P341337 | 1.356 | 4.94E-02 |
| polyadenylate-binding protein 2 | A_05_P488777 | 1.354 | 3.52E-02 |
| zinc finger protein 135^c^ | A_05_P283912 | 1.352 | 3.62E-02 |
| ap-1 complex subunit gamma-like 2 | A_05_P307107 | 1.352 | 4.42E-02 |
| visinin-like protein 1 | A_05_P434012 | 1.351 | 2.80E-02 |
| translocon-associated protein subunit beta precursor | A_05_P380892 | 1.350 | 2.71E-02 |
| 40s ribosomal protein s14 | A_05_P318497 | 1.350 | 2.67E-02 |
| cc chemokine | A_05_P351477 | 1.349 | 3.59E-02 |
| serine threonine-protein kinase mrck beta | A_05_P488102 | 1.349 | 2.57E-02 |
| zinc finger protein 521 | A_05_P304682 | 1.349 | 2.22E-02 |
| lysine-specific demethylase 4b^d^ | A_05_P425492 | 1.348 | 4.31E-02 |
| ATP citrate lyase | A_05_P412117 | 1.346 | 4.06E-02 |
| dolichyl-diphosphooligosaccharide--protein glycosyltransferase subunit stt3b | A_05_P333072 | 1.345 | 3.54E-02 |
| ecto-nox disulfide-thiol exchanger 2 | A_05_P477907 | 1.345 | 3.86E-02 |
| ring finger protein 122 | A_05_P296417 | 1.342 | 1.81E-02 |
| asparagine synthetase | A_05_P468147 | 1.340 | 3.08E-02 |
| calcitonin gene-related peptide type 1 receptor | A_05_P384017 | 1.340 | 1.95E-02 |
| beta-glucuronidase precursor | A_05_P307552 | 1.339 | 3.89E-02 |
| tyrosyl-tRNA mitochondrial | A_05_P338732 | 1.338 | 2.38E-02 |
| excitatory amino acid transporter 2-like | A_05_P322857 | 1.338 | 3.69E-02 |
| ubiquitin-conjugating enzyme e2 a | A_05_P472252 | 1.337 | 3.31E-02 |
| tropomyosin 2 | A_05_P261754 | 1.337 | 2.57E-02 |
| wd repeat domain 36 | A_05_P276174 | 1.336 | 3.97E-02 |
| calcium channel flower homolog^d^ | A_05_P321957 | 1.335 | 1.70E-02 |
| sarcalumenin precursor | A_05_P386127 | 1.333 | 3.17E-02 |
| DNA-3-methyladenine glycosylase | A_05_P320177 | 1.332 | 1.82E-02 |
| member ras oncogene family | A_05_P390927 | 1.332 | 4.52E-02 |
| nucleoside diphosphate-linked moiety x motif mitochondrial-like | A_05_P385867 | 1.332 | 4.93E-02 |
| PWWP domain-containing protein 2A^c^ | A_05_P418377 | 1.331 | 1.08E-02 |
| cdgsh iron-sulfur domain-containing protein 2-like | A_05_P427527 | 1.331 | 1.29E-02 |
| threonine aldolase 1^d^ | A_05_P331357 | 1.331 | 2.60E-02 |
| protein FAM8A1^c^ | A_05_P382847 | 1.330 | 2.08E-02 |
| steroidogenic acute regulatory protein | A_05_P315137 | 1.329 | 2.47E-02 |
| dual specificity phosphatase 6 | A_05_P303567 | 1.328 | 2.69E-02 |
| protein c-ets-2 | A_05_P471982 | 1.328 | 2.12E-02 |
| vimentin | A_05_P368522 | 1.325 | 1.58E-02 |
| aminopeptidase n | A_05_P270459 | 1.321 | 2.59E-02 |
| forkhead box p1 | A_05_P316972 | 1.321 | 4.02E-02 |
| c6orf64 homolog | A_05_P427697 | 1.319 | 2.68E-02 |
| anaphase-promoting complex subunit 11 | A_05_P252594 | 1.318 | 3.90E-02 |
| max-interacting protein 1 isoform a | A_05_P285327 | 1.317 | 2.37E-02 |
| acyl-protein thioesterase 2 | A_05_P428852 | 1.316 | 2.02E-02 |
| signal recognition particle 68 kda protein | A_05_P327287 | 1.316 | 4.26E-02 |
| cat eye syndrome chromosome candidate 5 homolog | A_05_P407832 | 1.315 | 3.93E-02 |
| translation initiation factor eif-2b subunit alpha | A_05_P484827 | 1.315 | 2.81E-02 |
| transmembrane protein 87A precursor^c^ | A_05_P330037 | 1.312 | 2.76E-02 |
| beta -glucosyltransferase | A_05_P449072 | 1.311 | 2.77E-02 |
| hormone-sensitive lipase | A_05_P336397 | 1.310 | 1.92E-02 |
| torsin family member b (torsin b) | A_05_P418417 | 1.307 | 3.93E-02 |
| zinc finger protein 576^d^ | A_05_P428482 | 1.306 | 1.63E-02 |
| trafficking protein particle complex subunit 2-like protein | A_05_P410297 | 1.306 | 4.90E-02 |
| interleukin 10 beta | A_05_P313527 | 1.300 | 4.94E-02 |
| CD83 antigen^c^ | A_05_P303347 | 1.300 | 3.54E-02 |
| cyclin d1 | A_05_P288287 | 1.296 | 4.15E-02 |
| coagulation factor x | A_05_P425897 | 1.295 | 3.35E-02 |
| gamma-glutamyltranspeptidase 1 precursor^c^ | A_05_P429217 | 1.294 | 4.82E-02 |
| adaptor-related protein complex beta 1 subunit | A_05_P382067 | 1.293 | 1.99E-02 |
| mitochondrial import inner membrane translocase subunit tim23 | A_05_P371107 | 1.293 | 2.91E-02 |
| thioredoxin-like protein 1 | A_05_P407997 | 1.292 | 4.61E-02 |
| cd209 antigen-like protein d^d^ | A_05_P395002 | 1.290 | 3.44E-02 |
| non-homologous end-joining factor 1 | A_05_P380652 | 1.289 | 4.28E-02 |
| queuine tRNA-ribosyltransferase | A_05_P412827 | 1.289 | 4.27E-02 |
| inhibitor of growth protein 1 | A_05_P326002 | 1.288 | 4.31E-02 |
| ribosomal protein l7a | A_05_P465882 | 1.288 | 4.57E-02 |
| transport protein sec61 subunit alpha | A_05_P377862 | 1.287 | 2.71E-02 |
| uridine phosphorylase 1 | A_05_P371462 | 1.287 | 4.87E-02 |
| 39s ribosomal protein mitochondrial-like | A_05_P415432 | 1.287 | 4.01E-02 |
| double-stranded RNA-binding protein staufen homolog 1 | A_05_P368277 | 1.286 | 4.54E-02 |
| graves disease carrier | A_05_P368732 | 1.284 | 2.94E-02 |
| phosphatase and tensin-like protein^d^ | A_05_P397977 | 1.284 | 2.88E-02 |
| calcitonin gene-related peptide 2-like | A_05_P331372 | 1.282 | 3.56E-02 |
| dehydrogenase reductase (sdr family) member 7 | A_05_P460132 | 1.279 | 4.17E-02 |
| carbohydrate sulfotransferase 11-like | A_05_P387582 | 1.277 | 3.69E-02 |
| pdz and lim domain protein 2 | A_05_P364727 | 1.276 | 4.93E-02 |
| heterogeneous nuclear ribonucleoprotein g | A_05_P436690 | 1.275 | 3.12E-02 |
| polycomb group protein asxl1 | A_05_P308767 | 1.275 | 4.48E-02 |
| ribonuclease p protein subunit p21 | A_05_P262351 | 1.272 | 4.23E-02 |
| ribokinase | A_05_P263224 | 1.272 | 3.64E-02 |
| lumican precursor | A_05_P276134 | 1.266 | 3.05E-02 |
| jagunal homolog 1 | A_05_P320547 | 1.265 | 3.51E-02 |
| t-cell immunomodulatory | A_05_P296127 | 1.265 | 4.41E-02 |
| phosphoribosylaminoimidazole succinocarboxamide synthetase^d^ | A_05_P434750 | 1.261 | 4.43E-02 |
| sjchgc03032 protein^d^ | A_05_P389924 | 1.261 | 4.72E-02 |
| Troponin I, fast skeletal muscle^c^ | A_05_P441982 | 1.260 | 3.62E-02 |
| lysosomal-associated transmembrane protein 4a | A_05_P481578 | 1.259 | 4.05E-02 |
| kinesin light chain 1 | A_05_P472037 | 1.258 | 4.25E-02 |
| ras-related protein rab-7a | A_05_P472847 | 1.256 | 4.75E-02 |
| Nuclease harbi1^d^ | A_05_P342347 | 1.255 | 3.13E-02 |
| zinc finger protein rfp-like^d^ | A_05_P312727 | 1.255 | 4.69E-02 |
| ADP-ribosylation factor-like protein 3 | A_05_P265169 | 1.255 | 4.75E-02 |
| eh domain-containing protein 1 | A_05_P270034 | 1.254 | 4.49E-02 |
| thioredoxin-related transmembrane protein 2 | A_05_P327352 | 1.254 | 4.29E-02 |
| TRAF3-interacting JNK-activating modulator^c^ | A_05_P418147 | 1.253 | 3.85E-02 |
| vaccinia related kinase 3 | A_05_P414512 | 1.253 | 4.79E-02 |
| brain-specific angiogenesis inhibitor 3 | A_05_P456022 | 1.253 | 4.09E-02 |
| down syndrome cell adhesion molecule | A_05_P383022 | 1.248 | 4.10E-02 |
| transforming protein precursor | A_05_P365637 | 1.248 | 4.10E-02 |
| isoform cra_b^d^ | A_05_P418572 | 1.247 | 4.81E-02 |
| transcription initiation factor tfiid subunit 6 | A_05_P479292 | 1.246 | 4.95E-02 |
| znf98 protein | A_05_P334912 | 1.235 | 4.40E-02 |
| hydroxyacyl-coenzyme a dehydrogenase 3-ketoacyl-coenzyme a thiolase enoyl-coenzyme a hydratase (trifunctional protein) beta subunit | A_05_P418787 | 1.235 | 4.66E-02 |
| ADP-dependent glucokinase | A_05_P279407 | 1.232 | 4.92E-02 |

^a^Fold change is the average difference in expression as measured by the microarray

^b^ Measures the significance of the difference in expression between the small and large fish.

^c^ Sequence was unnamed by Blast2go but named by Agilent

^d^ Identified as a different gene by Agilent

Genes with significant up-regulation in small fish across seasons are highlighted in green
