# Supplementary material for: Differential gene expression in small and large rainbow trout derived from two seasonal spawning groups
Source: BMC Genomics. 2014 Jan 22;15:57. doi: 10.1186/1471-2164-15-57 (PMC3931318; doi:10.1186/1471-2164-15-57)
Supplement: Additional file 11: Table S11 — Genes of unknown function up-regulated in the white muscle of large rainbow trout compared to small rainbow trout. [file 1471-2164-15-57-S11.docx]

| **Supplementary Table 11: Genes of unknown function up-regulated in the white muscle of large rainbow trout compared to small rainbow trout** | | | | | |
| --- | --- | --- | --- | --- | --- |
| **Gene Number** | **Fold change^a^** | **P value^b^** | **Gene Number** | **Fold change^a^** | **P value^b^** |
| ***Sept Fish*** |  |  | ***Sept Fish*** |  |  |
| A_05_P450047 | 2.217 | 1.05E-02 | A_05_P252009 | 2.155 | 6.04E-03 |
| A_05_P426617 | 1.996 | 1.52E-02 | A_05_P393907 | 1.931 | 1.70E-02 |
| A_05_P372697 | 1.898 | 1.95E-02 | A_05_P310457 | 1.761 | 3.67E-02 |
| A_05_P444217 | 1.745 | 3.76E-02 | A_05_P302187 | 1.745 | 2.01E-02 |
| A_05_P483072 | 1.689 | 2.33E-02 | A_05_P466507 | 1.686 | 2.33E-02 |
| A_05_P470532 | 1.658 | 1.88E-02 | A_05_P364857 | 1.656 | 3.95E-02 |
| A_05_P269399 | 1.595 | 3.67E-02 | A_05_P385852 | 1.534 | 2.09E-02 |
| A_05_P378107 | 1.515 | 2.25E-02 | A_05_P361762 | 1.481 | 3.59E-02 |
| A_05_P477497 | 1.471 | 4.56E-02 | A_05_P250769 | 1.451 | 4.00E-02 |
| A_05_P288237 | 1.433 | 3.15E-02 | A_05_P344722 | 1.422 | 4.63E-02 |
| A_05_P286427 | 1.401 | 4.42E-02 | A_05_P431462 | 1.387 | 4.82E-02 |
| ***Dec Fish*** |  |  | ***Dec Fish*** |  |  |
| A_05_P450247 | 3.125 | 2.68E-02 | A_05_P345477 | 3.012 | 4.88E-02 |
| A_05_P432727 | 2.994 | 2.46E-02 | A_05_P447112 | 2.849 | 1.35E-02 |
| A_05_P426322 | 2.833 | 4.18E-02 | A_05_P486327 | 2.793 | 7.78E-03 |
| A_05_P335672 | 2.747 | 3.75E-02 | A_05_P360187 | 2.500 | 1.64E-02 |
| A_05_P411087 | 2.469 | 4.68E-02 | A_05_P425227 | 2.415 | 3.77E-02 |
| A_05_P458722 | 2.299 | 2.00E-02 | A_05_P337642 | 2.278 | 1.85E-02 |
| A_05_P269559 | 2.247 | 4.32E-02 | A_05_P470872 | 2.179 | 1.09E-02 |
| A_05_P270899 | 2.114 | 4.57E-02 | A_05_P446727 | 2.110 | 4.69E-02 |
| A_05_P415087 | 2.075 | 1.97E-02 | A_05_P411587 | 2.070 | 3.53E-02 |
| A_05_P344087 | 2.049 | 4.30E-02 | A_05_P330247 | 2.028 | 4.63E-02 |
| A_05_P450992 | 2.024 | 4.20E-02 | A_05_P420182 | 2.008 | 2.26E-02 |
| A_05_P428717 | 2.000 | 2.76E-02 | A_05_P430677 | 1.972 | 3.00E-02 |
| A_05_P426512 | 1.972 | 2.21E-02 | A_05_P433452 | 1.965 | 3.46E-02 |
| A_05_P412947 | 1.946 | 2.56E-02 | A_05_P423732 | 1.938 | 2.65E-02 |
| A_05_P277887 | 1.931 | 3.27E-02 | A_05_P475397 | 1.927 | 3.52E-02 |
| A_05_P442462 | 1.916 | 1.96E-02 | A_05_P272429 | 1.908 | 3.20E-02 |
| A_05_P476246 | 1.898 | 2.84E-02 | A_05_P421117 | 1.890 | 4.70E-02 |
| A_05_P468332 | 1.890 | 4.61E-02 | A_05_P372252 | 1.887 | 2.30E-02 |
| A_05_P463222 | 1.883 | 3.42E-02 | A_05_P459382 | 1.876 | 3.24E-02 |
| A_05_P455442 | 1.862 | 3.07E-02 | A_05_P424692 | 1.838 | 4.47E-02 |
| A_05_P252574 | 1.828 | 2.61E-02 | A_05_P450837 | 1.828 | 2.32E-02 |
| A_05_P445287 | 1.799 | 4.51E-02 | A_05_P411172 | 1.795 | 4.19E-02 |
| A_05_P411657 | 1.792 | 2.35E-02 | A_05_P353087 | 1.786 | 4.90E-02 |
| A_05_P461187 | 1.776 | 3.31E-02 | A_05_P417707 | 1.761 | 3.00E-02 |
| A_05_P482472 | 1.754 | 2.98E-02 | A_05_P470912 | 1.745 | 3.46E-02 |
| A_05_P438317 | 1.739 | 3.47E-02 | A_05_P435957 | 1.733 | 4.86E-02 |
| A_05_P439627 | 1.733 | 3.79E-02 | A_05_P417482 | 1.733 | 2.27E-02 |
| A_05_P418037 | 1.721 | 4.51E-02 | A_05_P354167 | 1.718 | 4.80E-02 |
| A_05_P459282 | 1.715 | 2.37E-02 | A_05_P376437 | 1.712 | 2.40E-02 |
| A_05_P352727 | 1.706 | 4.40E-02 | A_05_P432602 | 1.704 | 3.55E-02 |
| A_05_P471082 | 1.704 | 3.18E-02 | A_05_P360572 | 1.701 | 3.80E-02 |
| A_05_P464287 | 1.686 | 4.94E-02 | A_05_P333917 | 1.686 | 4.31E-02 |
| A_05_P444122 | 1.684 | 4.79E-02 | A_05_P286807 | 1.678 | 3.15E-02 |
| A_05_P440577 | 1.675 | 4.99E-02 | A_05_P421997 | 1.675 | 4.79E-02 |
| A_05_P459572 | 1.672 | 4.34E-02 | A_05_P447362 | 1.672 | 4.59E-02 |
| A_05_P468742 | 1.669 | 2.60E-02 | A_05_P463957 | 1.664 | 4.43E-02 |
| A_05_P305652 | 1.664 | 4.84E-02 | A_05_P432032 | 1.661 | 3.32E-02 |
| A_05_P461777 | 1.656 | 2.64E-02 | A_05_P425197 | 1.653 | 4.29E-02 |
| A_05_P331242 | 1.653 | 2.28E-02 | A_05_P303652 | 1.642 | 4.65E-02 |
| A_05_P411952 | 1.634 | 4.86E-02 | A_05_P411662 | 1.629 | 4.78E-02 |
| A_05_P417352 | 1.629 | 3.99E-02 | A_05_P384997 | 1.626 | 2.66E-02 |
| A_05_P460242 | 1.610 | 3.29E-02 | A_05_P266724 | 1.600 | 4.39E-02 |
| A_05_P424532 | 1.600 | 3.57E-02 | A_05_P439117 | 1.595 | 3.95E-02 |
| A_05_P441827 | 1.592 | 4.62E-02 | A_05_P413082 | 1.587 | 3.88E-02 |
| A_05_P420047 | 1.572 | 4.12E-02 | A_05_P486882 | 1.570 | 4.57E-02 |
| A_05_P325547 | 1.565 | 4.77E-02 | A_05_P454242 | 1.565 | 4.12E-02 |
| A_05_P487977 | 1.565 | 2.82E-02 | A_05_P466292 | 1.546 | 4.15E-02 |
| A_05_P412057 | 1.546 | 4.83E-02 | A_05_P426862 | 1.527 | 4.90E-02 |
| A_05_P441932 | 1.522 | 4.95E-02 | A_05_P346057 | 1.520 | 3.24E-02 |
| A_05_P475032 | 1.515 | 3.84E-02 | A_05_P493831 | 1.513 | 4.60E-02 |
| A_05_P348377 | 1.497 | 4.57E-02 | A_05_P352417 | 1.460 | 4.17E-02 |
| A_05_P346797 | 1.418 | 4.74E-02 |  |  |  |

^a^Fold change is the average difference in expression as measured by the microarray

^b^ Measures the significance of the difference in expression between the small and large fish

Genes that are up-regulated in both large & small fish across seasons are highlighted in red
