# Supplementary material for: Differential gene expression in small and large rainbow trout derived from two seasonal spawning groups
Source: BMC Genomics. 2014 Jan 22;15:57. doi: 10.1186/1471-2164-15-57 (PMC3931318; doi:10.1186/1471-2164-15-57)
Supplement: Additional file 12: Table S12 — Genes of unknown function up-regulated in the white muscle of small rainbow trout compared to large rainbow trout. [file 1471-2164-15-57-S12.docx]

| **Supplementary Table 12: Genes of unknown function up-regulated in the white muscle of small rainbow trout compared to large rainbow trout** | | | | | |
| --- | --- | --- | --- | --- | --- |
| **Gene Number** | **Fold change^a^** | **P value^b^** | **Gene Number** | **Fold change^a^** | **P value^b^** |
| ***Sept Fish*** |  |  | ***Sept Fish*** |  |  |
| A_05_P337307 | 1.686 | 4.42E-02 | A_05_P385782 | 1.633 | 1.12E-02 |
| A_05_P289482 | 1.525 | 7.99E-03 | A_05_P474777 | 1.51 | 3.99E-02 |
| A_05_P476707 | 1.51 | 4.81E-02 | A_05_P300142 | 1.502 | 3.55E-02 |
| A_05_P413142 | 1.486 | 3.26E-02 | A_05_P479482 | 1.472 | 2.01E-02 |
| A_05_P427907 | 1.468 | 6.84E-03 | A_05_P471927 | 1.455 | 4.36E-02 |
| A_05_P456907 | 1.435 | 6.87E-03 | A_05_P432377 | 1.426 | 2.10E-03 |
| A_05_P354002 | 1.421 | 1.56E-02 | A_05_P353982 | 1.415 | 3.43E-02 |
| A_05_P489607 | 1.412 | 2.30E-02 | A_05_P360177 | 1.402 | 1.93E-02 |
| A_05_P345577 | 1.400 | 8.57E-03 | A_05_P312222 | 1.398 | 1.21E-02 |
| A_05_P453302 | 1.395 | 6.12E-03 | A_05_P455322 | 1.391 | 4.74E-02 |
| A_05_P395392 | 1.387 | 3.02E-03 | A_05_P457947 | 1.387 | 1.33E-02 |
| A_05_P437942 | 1.385 | 7.52E-03 | A_05_P281717 | 1.376 | 1.12E-02 |
| A_05_P471447 | 1.374 | 9.54E-03 | A_05_P356032 | 1.374 | 3.64E-02 |
| A_05_P403622 | 1.363 | 1.59E-02 | A_05_P403997 | 1.362 | 3.46E-02 |
| A_05_P341012 | 1.358 | 4.30E-02 | A_05_P485442 | 1.356 | 1.35E-02 |
| A_05_P468792 | 1.354 | 3.24E-02 | A_05_P409417 | 1.353 | 2.70E-02 |
| A_05_P465837 | 1.352 | 2.07E-02 | A_05_P355967 | 1.351 | 3.86E-02 |
| A_05_P340902 | 1.350 | 5.18E-03 | A_05_P463347 | 1.35 | 3.17E-02 |
| A_05_P345547 | 1.343 | 1.78E-02 | A_05_P415022 | 1.341 | 1.80E-02 |
| A_05_P492052 | 1.340 | 2.01E-02 | A_05_P463062 | 1.338 | 2.41E-02 |
| A_05_P441722 | 1.337 | 3.38E-02 | A_05_P413082 | 1.335 | 2.09E-02 |
| A_05_P334807 | 1.333 | 1.83E-02 | A_05_P332027 | 1.328 | 4.98E-02^d^ |
| A_05_P350012 | 1.322 | 2.37E-02 | A_05_P453702 | 1.322 | 2.90E-02 |
| A_05_P457262 | 1.322 | 2.63E-02 | A_05_P346462 | 1.318 | 1.10E-02 |
| A_05_P438262 | 1.314 | 2.02E-02 | A_05_P491797 | 1.313 | 2.00E-02 |
| A_05_P356337 | 1.312 | 4.23E-02 | A_05_P274764 | 1.311 | 1.84E-02 |
| A_05_P473252 | 1.307 | 2.19E-02 | A_05_P352827 | 1.307 | 3.48E-02 |
| A_05_P477252 | 1.305 | 1.58E-02 | A_05_P396987 | 1.305 | 4.93E-02 |
| A_05_P303447 | 1.304 | 2.46E-02 | A_05_P490032 | 1.301 | 1.64E-02 |
| A_05_P284202 | 1.301 | 2.97E-02 | A_05_P362732 | 1.297 | 1.74E-02 |
| A_05_P319582 | 1.296 | 2.96E-02 | A_05_P460882 | 1.293 | 4.82E-02 |
| A_05_P402472 | 1.289 | 2.44E-02 |  |  |  |
| ***Dec Fish*** |  |  | ***Dec Fish*** |  |  |
| A_05_P302187 | 4.352 | 5.19E-03 | A_05_P424372 | 3.052 | 2.79E-02 |
| A_05_P322407 | 2.262 | 2.38E-03 | A_05_P353147 | 2.224 | 8.80E-03 |
| A_05_P494567 | 2.199 | 3.33E-04 | A_05_P466242 | 2.182 | 5.50E-03 |
| A_05_P281317 | 2.177 | 2.64E-02 | A_05_P311427 | 2.119 | 7.98E-03 |
| A_05_P311282 | 2.049 | 1.68E-02 | A_05_P383462 | 2.040 | 1.08E-02 |
| A_05_P310942 | 2.018 | 1.13E-02 | A_05_P464996 | 2.017 | 3.30E-02 |
| A_05_P354507 | 2.010 | 2.27E-04 | A_05_P323532 | 2.003 | 1.25E-02 |
| A_05_P443052 | 1.923 | 3.12E-02 | A_05_P476302 | 1.891 | 3.47E-02 |
| A_05_P356707 | 1.885 | 1.74E-02 | A_05_P317932 | 1.868 | 6.00E-04 |
| A_05_P343552 | 1.865 | 2.80E-02 | A_05_P334177 | 1.865 | 3.90E-02 |
| A_05_P283622 | 1.858 | 4.19E-03 | A_05_P337177 | 1.848 | 5.33E-03 |
| A_05_P486167 | 1.845 | 1.20E-02 | A_05_P314432 | 1.835 | 8.07E-03 |
| A_05_P279427 | 1.819 | 3.39E-02 | A_05_P396307 | 1.811 | 2.55E-02 |
| A_05_P274139 | 1.803 | 4.08E-02 | A_05_P323792 | 1.802 | 3.63E-03 |
| A_05_P399557 | 1.799 | 9.29E-03 | A_05_P491055 | 1.796 | 4.18E-02 |
| A_05_P270679 | 1.791 | 1.88E-02 | A_05_P481437 | 1.784 | 3.51E-02 |
| A_05_P342367 | 1.778 | 2.82E-02 | A_05_P354002 | 1.774 | 3.33E-02 |
| A_05_P477452 | 1.774 | 2.51E-02 | A_05_P443887 | 1.753 | 2.36E-02 |
| A_05_P348222 | 1.747 | 9.38E-03 | A_05_P465697 | 1.747 | 2.55E-02 |
| A_05_P381427 | 1.739 | 2.80E-03 | A_05_P313457 | 1.738 | 2.08E-03 |
| A_05_P358362 | 1.730 | 1.69E-02 | A_05_P313337 | 1.728 | 7.23E-03 |
| A_05_P398452 | 1.719 | 5.49E-03 | A_05_P343832 | 1.718 | 4.61E-02 |
| A_05_P471047 | 1.717 | 6.74E-03 | A_05_P363272 | 1.706 | 3.90E-02 |
| A_05_P264784 | 1.693 | 1.08E-02 | A_05_P413643 | 1.689 | 3.54E-02 |
| A_05_P300487 | 1.684 | 1.13E-02 | A_05_P468462 | 1.681 | 2.63E-02 |
| A_05_P293952 | 1.671 | 1.12E-02 | A_05_P288917 | 1.663 | 7.94E-03 |
| A_05_P459227 | 1.663 | 4.61E-02 | A_05_P352317 | 1.658 | 1.91E-02 |
| A_05_P447442 | 1.658 | 4.03E-02 | A_05_P404117 | 1.657 | 1.12E-02 |
| A_05_P491632 | 1.656 | 3.06E-02 | A_05_P275404 | 1.656 | 4.37E-02 |
| A_05_P356832 | 1.655 | 3.37E-02 | A_05_P486207 | 1.654 | 1.62E-03 |
| A_05_P468142 | 1.651 | 6.26E-03 | A_05_P280412 | 1.646 | 6.45E-03 |
| A_05_P295077 | 1.641 | 7.74E-03 | A_05_P492332 | 1.641 | 7.52E-03 |
| A_05_P336727 | 1.635 | 3.39E-02 | A_05_P402597 | 1.634 | 4.01E-02 |
| A_05_P317047 | 1.633 | 2.60E-02 | A_05_P258834 | 1.631 | 4.21E-02 |
| A_05_P381152 | 1.628 | 1.66E-02 | A_05_P312457 | 1.628 | 2.28E-02 |
| A_05_P346282 | 1.626 | 6.06E-03 | A_05_P382352 | 1.620 | 1.36E-03 |
| A_05_P404682 | 1.618 | 3.13E-02 | A_05_P358522 | 1.617 | 2.01E-02 |
| A_05_P311112 | 1.614 | 1.59E-02 | A_05_P303067 | 1.614 | 1.06E-02 |
| A_05_P417312 | 1.611 | 2.83E-02 | A_05_P484412 | 1.610 | 1.84E-03 |
| A_05_P406307 | 1.610 | 1.10E-02 | A_05_P387712 | 1.609 | 2.25E-02 |
| A_05_P262664 | 1.609 | 4.40E-02 | A_05_P270274 | 1.607 | 4.49E-03 |
| A_05_P482076 | 1.605 | 2.28E-02 | A_05_P403102 | 1.602 | 2.67E-02 |
| A_05_P397137 | 1.601 | 1.41E-02 | A_05_P490697 | 1.601 | 2.58E-02 |
| A_05_P354807 | 1.601 | 3.14E-02 | A_05_P401812 | 1.599 | 1.57E-02 |
| A_05_P406013 | 1.598 | 2.70E-02 | A_05_P349842 | 1.597 | 2.69E-02 |
| A_05_P338902 | 1.596 | 4.42E-02 | A_05_P391197 | 1.596 | 2.10E-02 |
| A_05_P327852 | 1.595 | 1.24E-03 | A_05_P307442 | 1.595 | 6.63E-03 |
| A_05_P456362 | 1.592 | 3.74E-02 | A_05_P479477 | 1.589 | 8.21E-03 |
| A_05_P453862 | 1.586 | 2.15E-03 | A_05_P282712 | 1.586 | 1.22E-02 |
| A_05_P395727 | 1.585 | 1.03E-02 | A_05_P396017 | 1.581 | 2.58E-02 |
| A_05_P283042 | 1.581 | 2.86E-02 | A_05_P372987 | 1.580 | 7.18E-03 |
| A_05_P300777 | 1.580 | 3.60E-02 | A_05_P348152 | 1.579 | 1.26E-02 |
| A_05_P311177 | 1.578 | 1.26E-02 | A_05_P355942 | 1.578 | 1.75E-02 |
| A_05_P261544 | 1.578 | 2.82E-02 | A_05_P327022 | 1.577 | 4.15E-02 |
| A_05_P323492 | 1.577 | 4.28E-02 | A_05_P395966 | 1.573 | 5.73E-03 |
| A_05_P256324 | 1.568 | 7.07E-03 | A_05_P401662 | 1.567 | 2.86E-02 |
| A_05_P470522 | 1.565 | 1.63E-02 | A_05_P280972 | 1.564 | 1.53E-02 |
| A_05_P398977 | 1.561 | 4.97E-02 | A_05_P344472 | 1.560 | 1.72E-03 |
| A_05_P377787 | 1.560 | 2.51E-02 | A_05_P451027 | 1.559 | 4.09E-02 |
| A_05_P252214 | 1.558 | 2.32E-02 | A_05_P353987 | 1.557 | 2.17E-02 |
| A_05_P312802 | 1.556 | 1.40E-02 | A_05_P386752 | 1.555 | 2.76E-02 |
| A_05_P434137 | 1.554 | 3.80E-02 | A_05_P460567 | 1.553 | 4.76E-02 |
| A_05_P395692 | 1.550 | 2.62E-02 | A_05_P447107 | 1.550 | 2.46E-02 |
| A_05_P357622 | 1.550 | 4.28E-02 | A_05_P356432 | 1.550 | 3.20E-02 |
| A_05_P265949 | 1.549 | 2.24E-02 | A_05_P305027 | 1.548 | 2.84E-02 |
| A_05_P458439 | 1.547 | 4.97E-02 | A_05_P428392 | 1.546 | 2.97E-02 |
| A_05_P493902 | 1.545 | 2.51E-03 | A_05_P488792 | 1.545 | 3.82E-02 |
| A_05_P272179 | 1.543 | 4.16E-02 | A_05_P484417 | 1.542 | 3.10E-02 |
| A_05_P486642 | 1.541 | 4.36E-02 | A_05_P376980 | 1.541 | 3.87E-02 |
| A_05_P340882 | 1.540 | 2.51E-02 | A_05_P336432 | 1.538 | 9.32E-03 |
| A_05_P404697 | 1.538 | 4.81E-02 | A_05_P454532 | 1.534 | 1.72E-02 |
| A_05_P337807 | 1.533 | 3.06E-02 | A_05_P368202 | 1.529 | 3.18E-02 |
| A_05_P340122 | 1.527 | 1.46E-02 | A_05_P445159 | 1.525 | 5.12E-03 |
| A_05_P322337 | 1.525 | 1.19E-02 | A_05_P327652 | 1.525 | 4.00E-02 |
| A_05_P402517 | 1.524 | 3.24E-02 | A_05_P363477 | 1.524 | 2.18E-02 |
| A_05_P351482 | 1.523 | 1.55E-03 | A_05_P335352 | 1.523 | 5.43E-03 |
| A_05_P283857 | 1.523 | 1.97E-02 | A_05_P384627 | 1.523 | 4.98E-02 |
| A_05_P492517 | 1.522 | 1.71E-02 | A_05_P290787 | 1.520 | 1.21E-02 |
| A_05_P385382 | 1.520 | 4.72E-02 | A_05_P486452 | 1.518 | 9.06E-03 |
| A_05_P314407 | 1.518 | 4.59E-02 | A_05_P302607 | 1.517 | 3.53E-02 |
| A_05_P327602 | 1.517 | 3.39E-02 | A_05_P400217 | 1.516 | 1.34E-02 |
| A_05_P394392 | 1.516 | 1.56E-02 | A_05_P278977 | 1.516 | 3.15E-02 |
| A_05_P285237 | 1.516 | 3.77E-02 | A_05_P336977 | 1.515 | 3.35E-02 |
| A_05_P444871 | 1.514 | 2.41E-02 | A_05_P357282 | 1.513 | 1.13E-02 |
| A_05_P405612 | 1.512 | 2.13E-02 | A_05_P490987 | 1.511 | 9.23E-03 |
| A_05_P461212 | 1.511 | 1.46E-02 | A_05_P293422 | 1.509 | 4.45E-03 |
| A_05_P285932 | 1.509 | 4.82E-02 | A_05_P421557 | 1.509 | 4.06E-02 |
| A_05_P362667 | 1.508 | 8.61E-03 | A_05_P311252 | 1.508 | 8.33E-03 |
| A_05_P471677 | 1.508 | 3.42E-02 | A_05_P484467 | 1.507 | 1.64E-02 |
| A_05_P460057 | 1.507 | 3.63E-02 | A_05_P365277 | 1.506 | 1.10E-02 |
| A_05_P418772 | 1.504 | 2.18E-02 | A_05_P357252 | 1.503 | 3.85E-02 |
| A_05_P292452 | 1.503 | 3.76E-02 | A_05_P494457 | 1.501 | 4.35E-02 |
| A_05_P300344 | 1.500 | 2.72E-02 | A_05_P265669 | 1.500 | 3.69E-02 |
| A_05_P306522 | 1.499 | 3.41E-02 | A_05_P474812 | 1.499 | 3.54E-02 |
| A_05_P380062 | 1.498 | 1.96E-02 | A_05_P439527 | 1.497 | 2.19E-02 |
| A_05_P361742 | 1.496 | 3.62E-03 | A_05_P301837 | 1.496 | 1.38E-02 |
| A_05_P294277 | 1.496 | 2.30E-02 | A_05_P355987 | 1.496 | 4.40E-02 |
| A_05_P433162 | 1.494 | 4.61E-02 | A_05_P275084 | 1.494 | 4.07E-02 |
| A_05_P459802 | 1.493 | 2.15E-03 | A_05_P250519 | 1.492 | 2.03E-02 |
| A_05_P311987 | 1.492 | 3.94E-02 | A_05_P469412 | 1.491 | 4.71E-02 |
| A_05_P319687 | 1.490 | 3.30E-02 | A_05_P345572 | 1.489 | 1.38E-02 |
| A_05_P315547 | 1.489 | 1.31E-02 | A_05_P395966 | 1.573 | 5.73E-03 |
| A_05_P339862 | 1.486 | 3.84E-03 | A_05_P401662 | 1.567 | 2.86E-02 |
| A_05_P449937 | 1.485 | 4.94E-02 | A_05_P280972 | 1.564 | 1.53E-02 |
| A_05_P354792 | 1.484 | 7.84E-03 | A_05_P344472 | 1.560 | 1.72E-03 |
| A_05_P487187 | 1.482 | 4.49E-02 | A_05_P451027 | 1.559 | 4.09E-02 |
| A_05_P479237 | 1.480 | 4.70E-02 | A_05_P353987 | 1.557 | 2.17E-02 |
| A_05_P306692 | 1.479 | 2.16E-02 | A_05_P386752 | 1.555 | 2.76E-02 |
| A_05_P358447 | 1.478 | 3.97E-02 | A_05_P460567 | 1.553 | 4.76E-02 |
| A_05_P485967 | 1.474 | 4.96E-02 | A_05_P447107 | 1.550 | 2.46E-02 |
| A_05_P459922 | 1.472 | 1.06E-02 | A_05_P356432 | 1.550 | 3.20E-02 |
| A_05_P492842 | 1.470 | 2.26E-02 | A_05_P305027 | 1.548 | 2.84E-02 |
| A_05_P422302 | 1.468 | 3.87E-03 | A_05_P428392 | 1.546 | 2.97E-02 |
| A_05_P479692 | 1.468 | 4.62E-02 | A_05_P488792 | 1.545 | 3.82E-02 |
| A_05_P431132 | 1.464 | 2.47E-02 | A_05_P484417 | 1.542 | 3.10E-02 |
| A_05_P428972 | 1.463 | 1.17E-02 | A_05_P376980 | 1.541 | 3.87E-02 |
| A_05_P420352 | 1.462 | 1.69E-02 | A_05_P336432 | 1.538 | 9.32E-03 |
| A_05_P470593 | 1.461 | 3.33E-02 | A_05_P454532 | 1.534 | 1.72E-02 |
| A_05_P361872 | 1.460 | 4.46E-02 | A_05_P368202 | 1.529 | 3.18E-02 |
| A_05_P291432 | 1.458 | 1.18E-02 | A_05_P445159 | 1.525 | 5.12E-03 |
| A_05_P438512 | 1.457 | 3.41E-02 | A_05_P327652 | 1.525 | 4.00E-02 |
| A_05_P313247 | 1.456 | 2.23E-02 | A_05_P363477 | 1.524 | 2.18E-02 |
| A_05_P311647 | 1.455 | 2.06E-02 | A_05_P335352 | 1.523 | 5.43E-03 |
| A_05_P484547 | 1.454 | 2.89E-02 | A_05_P384627 | 1.523 | 4.98E-02 |
| A_05_P347647 | 1.449 | 4.61E-02 | A_05_P290787 | 1.520 | 1.21E-02 |
| A_05_P459812 | 1.446 | 1.16E-02 | A_05_P486452 | 1.518 | 9.06E-03 |
| A_05_P313667 | 1.443 | 4.36E-03 | A_05_P302607 | 1.517 | 3.53E-02 |
| A_05_P466812 | 1.443 | 2.55E-02 | A_05_P400217 | 1.516 | 1.34E-02 |
| A_05_P434657 | 1.440 | 2.69E-02 | A_05_P278977 | 1.516 | 3.15E-02 |
| A_05_P349282 | 1.436 | 4.38E-02 | A_05_P336977 | 1.515 | 3.35E-02 |
| A_05_P380087 | 1.435 | 2.85E-02 | A_05_P357282 | 1.513 | 1.13E-02 |
| A_05_P443195 | 1.434 | 1.33E-02 | A_05_P490987 | 1.511 | 9.23E-03 |
| A_05_P455517 | 1.433 | 4.85E-02 | A_05_P293422 | 1.509 | 4.45E-03 |
| A_05_P290417 | 1.427 | 1.45E-02 | A_05_P421557 | 1.509 | 4.06E-02 |
| A_05_P432692 | 1.427 | 4.93E-02 | A_05_P311252 | 1.508 | 8.33E-03 |
| A_05_P442127 | 1.425 | 1.39E-02 | A_05_P484467 | 1.507 | 1.64E-02 |
| A_05_P338572 | 1.423 | 4.75E-02 | A_05_P365277 | 1.506 | 1.10E-02 |
| A_05_P486097 | 1.422 | 4.15E-02 | A_05_P357252 | 1.503 | 3.85E-02 |
| A_05_P400812 | 1.420 | 1.23E-02 | A_05_P494457 | 1.501 | 4.35E-02 |
| A_05_P364367 | 1.420 | 3.26E-02 | A_05_P265669 | 1.500 | 3.69E-02 |
| A_05_P404067 | 1.419 | 1.93E-02 | A_05_P474812 | 1.499 | 3.54E-02 |
| A_05_P447697 | 1.419 | 4.80E-02 | A_05_P439527 | 1.497 | 2.19E-02 |
| A_05_P423767 | 1.417 | 3.66E-02 | A_05_P301837 | 1.496 | 1.38E-02 |
| A_05_P302412 | 1.415 | 4.82E-02 | A_05_P355987 | 1.496 | 4.40E-02 |
| A_05_P480792 | 1.413 | 4.42E-02 | A_05_P275084 | 1.494 | 4.07E-02 |
| A_05_P302713 | 1.412 | 4.35E-02 | A_05_P250519 | 1.492 | 2.03E-02 |
| A_05_P485242 | 1.411 | 2.82E-02 | A_05_P469412 | 1.491 | 4.71E-02 |
| A_05_P413377 | 1.410 | 4.37E-02 | A_05_P345572 | 1.489 | 1.38E-02 |
| A_05_P470487 | 1.408 | 4.04E-02 | A_05_P395966 | 1.573 | 5.73E-03 |
| A_05_P326822 | 1.406 | 2.97E-02 | A_05_P401662 | 1.567 | 2.86E-02 |
| A_05_P426407 | 1.405 | 2.48E-02 | A_05_P280972 | 1.564 | 1.53E-02 |
| A_05_P420072 | 1.403 | 1.54E-02 | A_05_P344472 | 1.560 | 1.72E-03 |
| A_05_P450892 | 1.402 | 3.97E-02 | A_05_P451027 | 1.559 | 4.09E-02 |
| A_05_P325002 | 1.401 | 4.13E-02 | A_05_P353987 | 1.557 | 2.17E-02 |
| A_05_P397197 | 1.400 | 4.69E-02 | A_05_P386752 | 1.555 | 2.76E-02 |
| A_05_P491577 | 1.398 | 1.26E-02 | A_05_P460567 | 1.553 | 4.76E-02 |
| A_05_P483872 | 1.396 | 3.60E-02 | A_05_P447107 | 1.550 | 2.46E-02 |
| A_05_P469842 | 1.395 | 3.22E-02 | A_05_P356432 | 1.550 | 3.20E-02 |
| A_05_P349022 | 1.391 | 3.67E-02 | A_05_P305027 | 1.548 | 2.84E-02 |
| A_05_P387627 | 1.389 | 1.07E-02 | A_05_P428392 | 1.546 | 2.97E-02 |
| A_05_P352362 | 1.388 | 3.02E-02 | A_05_P488792 | 1.545 | 3.82E-02 |
| A_05_P376197 | 1.387 | 4.65E-02 | A_05_P484417 | 1.542 | 3.10E-02 |
| A_05_P468538 | 1.387 | 2.17E-02 | A_05_P376980 | 1.541 | 3.87E-02 |
| A_05_P299972 | 1.386 | 2.39E-02 | A_05_P336432 | 1.538 | 9.32E-03 |
| A_05_P361217 | 1.383 | 1.29E-02 | A_05_P454532 | 1.534 | 1.72E-02 |
| A_05_P447152 | 1.382 | 3.26E-02 | A_05_P368202 | 1.529 | 3.18E-02 |
| A_05_P317202 | 1.379 | 3.18E-02 | A_05_P445159 | 1.525 | 5.12E-03 |
| A_05_P272049 | 1.378 | 4.08E-02 | A_05_P327652 | 1.525 | 4.00E-02 |
| A_05_P284117 | 1.378 | 4.21E-02 | A_05_P363477 | 1.524 | 2.18E-02 |
| A_05_P356918 | 1.377 | 2.26E-02 | A_05_P335352 | 1.523 | 5.43E-03 |
| A_05_P443017 | 1.374 | 3.56E-02 | A_05_P384627 | 1.523 | 4.98E-02 |
| A_05_P257279 | 1.372 | 3.85E-02 | A_05_P290787 | 1.520 | 1.21E-02 |
| A_05_P449572 | 1.371 | 3.62E-02 | A_05_P486452 | 1.518 | 9.06E-03 |
| A_05_P493647 | 1.366 | 3.66E-02 | A_05_P302607 | 1.517 | 3.53E-02 |
| A_05_P337222 | 1.362 | 6.43E-03 | A_05_P400217 | 1.516 | 1.34E-02 |
| A_05_P344852 | 1.362 | 9.60E-03 | A_05_P278977 | 1.516 | 3.15E-02 |
| A_05_P289457 | 1.360 | 1.70E-02 | A_05_P336977 | 1.515 | 3.35E-02 |
| A_05_P299157 | 1.359 | 3.30E-02 | A_05_P357282 | 1.513 | 1.13E-02 |
| A_05_P363502 | 1.358 | 4.73E-02 | A_05_P490987 | 1.511 | 9.23E-03 |
| A_05_P403992 | 1.357 | 4.36E-02 | A_05_P293422 | 1.509 | 4.45E-03 |
| A_05_P344492 | 1.357 | 3.42E-02 | A_05_P421557 | 1.509 | 4.06E-02 |
| A_05_P319467 | 1.353 | 3.03E-02 | A_05_P311252 | 1.508 | 8.33E-03 |
| A_05_P462757 | 1.351 | 3.36E-02 | A_05_P484467 | 1.507 | 1.64E-02 |
| A_05_P278542 | 1.349 | 4.39E-02 | A_05_P365277 | 1.506 | 1.10E-02 |
| A_05_P355357 | 1.348 | 9.04E-03 | A_05_P357252 | 1.503 | 3.85E-02 |
| A_05_P258114 | 1.346 | 3.71E-02 | A_05_P494457 | 1.501 | 4.35E-02 |
| A_05_P385787 | 1.342 | 8.86E-03 | A_05_P265669 | 1.500 | 3.69E-02 |
| A_05_P432057 | 1.338 | 4.90E-02 | A_05_P474812 | 1.499 | 3.54E-02 |
| A_05_P343467 | 1.337 | 4.09E-02 | A_05_P439527 | 1.497 | 2.19E-02 |
| A_05_P428797 | 1.335 | 3.14E-02 | A_05_P301837 | 1.496 | 1.38E-02 |
| A_05_P475937 | 1.333 | 1.66E-02 | A_05_P355987 | 1.496 | 4.40E-02 |
| A_05_P275239 | 1.332 | 1.51E-02 | A_05_P275084 | 1.494 | 4.07E-02 |
| A_05_P492717 | 1.329 | 2.40E-02 | A_05_P250519 | 1.492 | 2.03E-02 |
| A_05_P460357 | 1.327 | 4.35E-02 | A_05_P469412 | 1.491 | 4.71E-02 |
| A_05_P278742 | 1.327 | 2.59E-02 | A_05_P345572 | 1.489 | 1.38E-02 |
| A_05_P404342 | 1.323 | 1.49E-02 | A_05_P395966 | 1.573 | 5.73E-03 |
| A_05_P402907 | 1.321 | 2.88E-02 | A_05_P401662 | 1.567 | 2.86E-02 |
| A_05_P283037 | 1.321 | 4.74E-02 | A_05_P280972 | 1.564 | 1.53E-02 |
| A_05_P344212 | 1.317 | 4.89E-02 | A_05_P344472 | 1.560 | 1.72E-03 |
| A_05_P284682 | 1.316 | 2.79E-02 | A_05_P451027 | 1.559 | 4.09E-02 |
| A_05_P353382 | 1.313 | 3.16E-02 | A_05_P353987 | 1.557 | 2.17E-02 |
| A_05_P468137 | 1.311 | 4.59E-02 | A_05_P386752 | 1.555 | 2.76E-02 |
| A_05_P283537 | 1.308 | 2.55E-02 | A_05_P460567 | 1.553 | 4.76E-02 |
| A_05_P465547 | 1.305 | 2.95E-02 | A_05_P447107 | 1.550 | 2.46E-02 |
| A_05_P297112 | 1.301 | 4.80E-02 | A_05_P356432 | 1.550 | 3.20E-02 |
| A_05_P256409 | 1.299 | 4.12E-02 | A_05_P305027 | 1.548 | 2.84E-02 |
| A_05_P399357 | 1.298 | 3.18E-02 | A_05_P428392 | 1.546 | 2.97E-02 |
| A_05_P493882 | 1.292 | 3.77E-02 | A_05_P488792 | 1.545 | 3.82E-02 |
| A_05_P394847 | 1.292 | 4.41E-02 | A_05_P484417 | 1.542 | 3.10E-02 |
| A_05_P403427 | 1.291 | 4.43E-02 | A_05_P376980 | 1.541 | 3.87E-02 |
| A_05_P312972 | 1.288 | 4.38E-02 | A_05_P336432 | 1.538 | 9.32E-03 |
| A_05_P415392 | 1.281 | 2.45E-02 | A_05_P454532 | 1.534 | 1.72E-02 |
| A_05_P350957 | 1.277 | 4.33E-02 | A_05_P368202 | 1.529 | 3.18E-02 |
| A_05_P405132 | 1.275 | 5.00E-02 | A_05_P445159 | 1.525 | 5.12E-03 |
| A_05_P282847 | 1.273 | 3.72E-02 | A_05_P327652 | 1.525 | 4.00E-02 |
| A_05_P264769 | 1.267 | 4.57E-02 | A_05_P363477 | 1.524 | 2.18E-02 |
| A_05_P284597 | 1.265 | 3.47E-02 | A_05_P335352 | 1.523 | 5.43E-03 |
| A_05_P317242 | 1.257 | 4.62E-02 | A_05_P384627 | 1.523 | 4.98E-02 |
| A_05_P303677 | 1.252 | 3.90E-02 | A_05_P290787 | 1.52 | 1.21E-02 |
| A_05_P374862 | 1.244 | 4.45E-02 | A_05_P486452 | 1.518 | 9.06E-03 |
| A_05_P302607 | 1.517 | 3.53E-02 |  |  |  |

^a^Fold change is the average difference in expression as measured by the microarray

^b^Measures the significance of the difference in expression between the small and large fish

**^d^** Guassian p-value < 0.05, t-test p-value > 0.05

Genes with significant up-regulation in small fish across seasons are highlighted in green

Genes that are up-regulated in both large & small fish across seasons are highlighted in red
