# Supplementary material for: Differential gene expression in small and large rainbow trout derived from two seasonal spawning groups
Source: BMC Genomics. 2014 Jan 22;15:57. doi: 10.1186/1471-2164-15-57 (PMC3931318; doi:10.1186/1471-2164-15-57)
Supplement: Additional file 17: Table S17 — Genes Up-regulated in White Muscle in Large Rainbow Trout compared to Small Rainbow Trout within the GO ‘response to external stimulus’ category. [file 1471-2164-15-57-S17.docx]

| **Supplementary Table 17a: Genes Up-regulated in White Muscle in Large Rainbow Trout compared to Small Rainbow Trout within the GO ‘response to external stimulus’ category** | | | |
| --- | --- | --- | --- |
| **Gene Name** | **Gene Number** | **Fold change^a^** | **p-value^b^** |
| ***Sept fish*** |  |  |  |
| max protein | A_05_P478412 | 5.618 | 4.14E-03 |
| fibrinogen gamma chain | A_05_P480537 | 2.625 | 3.06E-02 |
| fibrinogen gamma chain | A_05_P364872 | 2.584 | 3.24E-02 |
| fibrinogen alpha chain | A_05_P332052 | 2.506 | 1.24E-02 |
| beta-enolase-like isoform 1 | A_05_P482637 | 2.500 | 4.06E-02 |
| fibrinogen gamma chain | A_05_P464657 | 2.463 | 1.82E-02 |
| pyruvate kinase | A_05_P253514 | 2.381 | 2.45E-02 |
| fibrinogen gamma polypeptide | A_05_P450362 | 2.045 | 4.47E-02 |
| complement component c3 | A_05_P433922 | 2.024 | 2.44E-02 |
| pyruvate kinase | A_05_P390772 | 2.004 | 2.52E-02 |
| serum albumin precursor | A_05_P368112 | 1.934 | 3.96E-02 |
| coagulation factor ii precursor | A_05_P364912 | 1.919 | 2.10E-02 |
| Ca2+-dependent complex c1r c1s subunit | A_05_P457602 | 1.919 | 3.93E-02 |
| beta-2-glycoprotein 1-like | A_05_P275734 | 1.890 | 3.57E-02 |
| complement component c9 | A_05_P485237 | 1.748 | 4.63E-02 |
| Telethonin | A_05_P263889 | 1.695 | 1.79E-02 |
| Telethonin | A_05_P413737 | 1.616 | 3.15E-02 |
| c1 inhibitor | A_05_P265959 | 1.548 | 4.31E-02 |
| antithrombin-iii precursor | A_05_P445252 | 1.527 | 4.36E-02 |
| fibronectin precursor | A_05_P263834 | 1.458 | 2.50E-02 |
| complement c3-like | A_05_P490817 | 1.439 | 4.24E-02 |
| antithrombin-iii precursor | A_05_P249134 | 1.346 | 2.91E-02 |
| ***Dec Fish*** |  |  |  |
| fibrinogen gamma polypeptide | A_05_P450362 | 25.126 | 1.71E-02 |
| max protein | A_05_P478412 | 2.681 | 2.36E-02 |
| annexin a1 | A_05_P287152 | 2.506 | 1.91E-02 |
| growth factor receptor-bound protein 14 | A_05_P434562 | 2.299 | 2.92E-02 |
| collagenase 3 precursor | A_05_P327062 | 2.088 | 2.05E-02 |
| thioredoxin-interacting protein | A_05_P364437 | 2.037 | 3.55E-02 |
| calponin 2 | A_05_P411697 | 2.024 | 1.58E-02 |
| flna protein | A_05_P251164 | 1.972 | 3.79E-02 |
| nf-kappa-b inhibitor zeta | A_05_P301692 | 1.919 | 2.08E-02 |
| nedd4 family-interacting protein 1 | A_05_P369097 | 1.916 | 3.72E-02 |
| coagulation factor vii | A_05_P333597 | 1.859 | 2.08E-02 |
| plasminogen activator inhibitor 1 precursor | A_05_P285012 | 1.821 | 2.52E-02 |
| dystonin isoform 2 | A_05_P440912 | 1.815 | 4.53E-02 |
| transcription factor jun-d | A_05_P473177 | 1.802 | 3.29E-02 |
| collagen alpha-1 chain-like | A_05_P335067 | 1.799 | 4.26E-02 |
| latexin | A_05_P407002 | 1.761 | 2.49E-02 |
| collagen type i alpha 1 | A_05_P430717 | 1.748 | 4.01E-02 |
| microtubule-associated protein 1 light chain 3 beta | A_05_P415832 | 1.742 | 3.45E-02 |
| collagen type i alpha 2 | A_05_P413407 | 1.709 | 2.85E-02 |
| nedd4 family-interacting protein 1 | A_05_P419047 | 1.553 | 4.53E-02 |
| tetraspanin-8 | A_05_P429832 | 1.522 | 3.68E-02 |
| cystathionine beta-synthase | A_05_P269379 | 1.484 | 4.83E-02 |

| **Supplementary Table 17b: Named Genes Up-regulated in White Muscle in Small Rainbow Trout compared to Large Rainbow Trout within GO ‘response to external stimulus’ category** | | | |
| --- | --- | --- | --- |
| **Gene Name** | **Gene Number** | **Fold change^a^** | **p-value^b^** |
| ***Sept Fish*** |  |  |  |
| tyrosine-protein phosphatase non-receptor type 12 | A_05_P478272 | 2.013 | 7.26E-03 |
| platelet glycoprotein 4 | A_05_P270029 | 1.442 | 2.62E-02 |
| proteasome subunit alpha type-6 | A_05_P436627 | 1.435 | 2.36E-02 |
| aminoacyl trna synthase complex-interacting multifunctional protein 1 | A_05_P407602 | 1.410 | 3.00E-02 |
| dual specificity phosphatase 1 | A_05_P383357 | 1.406 | 2.28E-02 |
| thioredoxin interacting protein | A_05_P395257 | 1.393 | 4.04E-02 |
| polyadenylate-binding protein 4 isoform 3 | A_05_P450567 | 1.380 | 3.48E-02 |
| transcription factor | A_05_P308537 | 1.363 | 1.88E-02 |
| thrombospondin 4 | A_05_P300817 | 1.362 | 2.32E-02 |
| glutathione synthetase | A_05_P489127 | 1.349 | 4.67E-02 |
| coagulation factor x precursor | A_05_P457147 | 1.347 | 3.04E-02 |
| high mobility group protein b2 | A_05_P253324 | 1.325 | 1.27E-02 |
| calmodulin | A_05_P377098 | 1.301 | 3.56E-02 |
| monoglyceride lipase | A_05_P392532 | 1.286 | 3.42E-02 |
| selenoprotein s | A_05_P276549 | 1.270 | 4.49E-02 |
| fast myotomal muscle tropomyosin | A_05_P464747 | 1.263 | 3.19E-02 |
| 5-aminolevulinate mitochondrial | A_05_P467616 | 1.262 | 4.84E-02 |
| capping protein (actin filament) muscle z- beta | A_05_P410762 | 1.251 | 4.63E-02 |
| thymidine phosphorylase | A_05_P411652 | 1.246 | 3.92E-02 |
| interferon-induced helicase c domain-containing protein 1 | A_05_P486287 | 1.205 | 4.53E-02 |
| mapk mak mrk overlapping kinase | A_05_P416647 | 1.570 | 1.62E-03 |

^a^ Fold change is the average difference in expression as measured by the microarray

^b^ Measures the significance of the difference in expression between the small and large fish.

Probe Ids with proportionally higher counts in large Sept. fish within the GO ‘response to external stimulus’ category.

Probe Ids with proportionally lower counts in large Dec. fish and small Sept. Fish within the GO ‘response to external stimulus’ category.
