# Supplementary material for: Differential gene expression in small and large rainbow trout derived from two seasonal spawning groups
Source: BMC Genomics. 2014 Jan 22;15:57. doi: 10.1186/1471-2164-15-57 (PMC3931318; doi:10.1186/1471-2164-15-57)
Supplement: Additional file 18: Table S18 — Genes Up-regulated in White Muscle in Small Rainbow Trout compared to Large Rainbow Trout within the GO ‘establishment of localization’ category. [file 1471-2164-15-57-S18.docx]

| **Supplementary Table 18a: Genes Up-regulated in White Muscle in Small Rainbow Trout compared to Large Rainbow Trout within the GO ‘establishment of localization’ category** | | | |
| --- | --- | --- | --- |
| **Gene Name** | **Gene Number** | **Fold change^a^** | **p-value^b^** |
| ***Sept Fish*** |  |  |  |
| alpha actin | A_05_P449267 | 2.550 | 9.65E-04 |
| calcium calmodulin-dependent protein kinase (kinase) ii delta 2 | A_05_P304402 | 1.819 | 3.67E-02 |
| 80 kda mcm3-associated protein | A_05_P351672 | 1.667 | 2.15E-02 |
| 60s ribosomal export protein nmd3 | A_05_P411892 | 1.592 | 4.21E-02 |
| voltage-dependent anion-selective channel protein 2 | A_05_P411937 | 1.560 | 3.30E-02 |
| 6- muscle type | A_05_P467772 | 1.532 | 2.06E-02 |
| cytochrome c | A_05_P419817 | 1.449 | 2.00E-02 |
| platelet glycoprotein 4 | A_05_P270029 | 1.442 | 2.62E-02 |
| electron-transferring-flavoprotein dehydrogenase | A_05_P308382 | 1.433 | 4.30E-03 |
| voltage-dependent anion-selective channel protein 2 | A_05_P420122 | 1.432 | 1.37E-02 |
| t-complex protein 1 subunit beta | A_05_P418142 | 1.426 | 2.27E-02 |
| der1-like domain member 2 | A_05_P478862 | 1.420 | 4.00E-02 |
| solute carrier family 22 member 6-a | A_05_P377262 | 1.419 | 2.27E-02 |
| ras-related protein rab-2a | A_05_P391272 | 1.409 | 1.50E-02 |
| thioredoxin interacting protein | A_05_P395257 | 1.393 | 4.04E-02 |
| sh3 domain-containing protein 19-like | A_05_P336442 | 1.389 | 1.43E-02 |
| myh9 partial | A_05_P472392 | 1.376 | 4.18E-02 |
| protein cappuccino homolog | A_05_P421022 | 1.374 | 3.19E-02 |
| transcription factor | A_05_P308537 | 1.363 | 1.88E-02 |
| member ras oncogene family | A_05_P475987 | 1.358 | 3.73E-02 |
| splicing arginine serine-rich 11 | A_05_P450082 | 1.340 | 4.65E-02 |
| atp synthase subunit mitochondrial precursor | A_05_P409472 | 1.335 | 5.83E-03 |
| myotubularin-related protein 2 | A_05_P476167 | 1.331 | 3.95E-02 |
| 60s ribosomal protein l11 | A_05_P440812 | 1.330 | 4.52E-02 |
| probable glutamate receptor precursor | A_05_P302932 | 1.327 | 1.49E-02 |
| membrane-bound transcription factor site 1 | A_05_P489444 | 1.323 | 1.60E-02 |
| ubiquitin c | A_05_P418397 | 1.320 | 3.25E-02 |
| calcium calmodulin-dependent protein kinase iv | A_05_P455732 | 1.318 | 2.40E-02 |
| voltage-dependent anion-selective channel protein 2 | A_05_P251854 | 1.314 | 2.93E-02 |
| Calmodulin | A_05_P377098 | 1.301 | 3.56E-02 |
| protoporphyrinogen oxidase | A_05_P302012 | 1.291 | 1.37E-02 |
| carnitine o-acetyltransferase | A_05_P272599 | 1.288 | 4.03E-02 |
| platelet-activating factor acetylhydrolase ib subunit alpha | A_05_P368509 | 1.281 | 3.94E-02 |
| homeobox protein six2 | A_05_P346397 | 1.277 | 2.82E-02 |
| selenoprotein s | A_05_P276549 | 1.270 | 4.49E-02 |
| graves disease carrier | A_05_P368732 | 1.267 | 3.35E-02 |
| fast myotomal muscle tropomyosin | A_05_P464747 | 1.263 | 3.19E-02 |
| cleavage and polyadenylation specificity factor subunit 1 | A_05_P347667 | 1.258 | 3.57E-02 |
| guanine nucleotide-binding protein subunit beta-2-like 1 | A_05_P434467 | 1.257 | 3.70E-02 |
| tho complex subunit 5 homolog | A_05_P423802 | 1.216 | 4.69E-02 |
| protein fat-free homolog | A_05_P372287 | 1.211 | 4.94E-02 |

| **Supplementary Table 18b: Genes Up-regulated in White Muscle in Large Rainbow Trout compared to Small Rainbow Trout within the GO ‘establishment of localization’ category** | | | |
| --- | --- | --- | --- |
| **Gene Name** | **Gene Number** | **Fold change^a^** | **p-value^b^** |
| ***Sept fish*** |  |  |  |
| ADP/ATP translocase 2 | A_05_P276614 | 4.000 | 8.42E-03 |
| fibrinogen gamma chain | A_05_P480537 | 2.625 | 3.06E-02 |
| fibrinogen gamma chain | A_05_P364872 | 2.584 | 3.24E-02 |
| retinol-binding protein 2 | A_05_P254734 | 2.481 | 2.03E-02 |
| fibrinogen gamma chain | A_05_P464657 | 2.463 | 1.82E-02 |
| warm temperature acclimation-related 65 kda protein | A_05_P420737 | 2.227 | 5.84E-03 |
| alpha-1-microglobulin bikunin precursor | A_05_P332632 | 2.137 | 3.93E-02 |
| liver-type fatty acid-binding protein | A_05_P454457 | 2.066 | 2.85E-02 |
| fibrinogen gamma polypeptide | A_05_P450362 | 2.045 | 4.47E-02 |
| warm temperature acclimation-related 65 kda protein | A_05_P251959 | 1.949 | 1.01E-02 |
| liver-type fatty acid-binding protein | A_05_P263874 | 1.949 | 4.58E-02 |
| serum albumin precursor | A_05_P368112 | 1.934 | 3.96E-02 |
| beta-2-glycoprotein 1-like | A_05_P275734 | 1.890 | 3.57E-02 |
| retinol binding protein cellular | A_05_P486737 | 1.890 | 2.23E-02 |
| liver basic fatty acid binding protein | A_05_P455512 | 1.887 | 4.92E-02 |
| cytochrome b | A_05_P486612 | 1.883 | 2.76E-02 |
| apolipoprotein a-i | A_05_P249389 | 1.786 | 1.51E-02 |
| hemoglobin subunit alpha^c^ | A_05_P491417 | 1.776 | 1.43E-02 |
| hemoglobin subunit alpha | A_05_P449312 | 1.745 | 1.45E-02 |
| Telethonin | A_05_P263889 | 1.695 | 1.79E-02 |
| ap-1 complex subunit gamma-1 | A_05_P427757 | 1.686 | 3.83E-02 |
| hemoglobin subunit beta-1 | A_05_P453042 | 1.686 | 1.69E-02 |
| hemoglobin subunit alpha | A_05_P249524 | 1.664 | 3.55E-02 |
| Telethonin | A_05_P413737 | 1.616 | 3.15E-02 |
| hemoglobin subunit beta-1 | A_05_P365862 | 1.550 | 4.28E-02 |
| c1 inhibitor | A_05_P265959 | 1.548 | 4.31E-02 |
| ceruloplasmin | A_05_P365482 | 1.536 | 4.43E-02 |
| ribosomal protein s13 | A_05_P471392 | 1.437 | 3.20E-02 |

^a^ Fold change is the average difference in expression as measured by the microarray

^b^ Measures the significance of the difference in expression between the small and large fish.

^c^ Sequence was unnamed by Blast2go but named by Agilent

Colours in Gene Number column:

Probe Ids with proportionally higher counts in small Sept. fish within the GO ‘establishment of localization’ category.

Probe Ids with proportionally lower counts in large Sept. Fish within the GO ‘establishment of localization’ category.
