# Supplementary material for: Differential gene expression in small and large rainbow trout derived from two seasonal spawning groups
Source: BMC Genomics. 2014 Jan 22;15:57. doi: 10.1186/1471-2164-15-57 (PMC3931318; doi:10.1186/1471-2164-15-57)
Supplement: Additional file 19: Table S19 — Genes Up-regulated in White Muscle in Large Rainbow Trout compared to Small Rainbow Trout within GO ‘response to stress’ category. [file 1471-2164-15-57-S19.docx]

| **Supplementary Table 19a: Genes Up-regulated in White Muscle in Large Rainbow Trout compared to Small Rainbow Trout within GO ‘response to stress’ category** | | | |
| --- | --- | --- | --- |
| **Gene Name** | **Gene Number** | **Fold change^a^** | **p-value^b^** |
| ***Sept fish*** |  |  |  |
| max protein | A_05_P478412 | 5.618 | 4.14E-03 |
| hig1 domain family member 2a | A_05_P433247 | 2.801 | 3.92E-02 |
| fibrinogen gamma chain | A_05_P480537 | 2.625 | 3.06E-02 |
| fibrinogen gamma chain | A_05_P364872 | 2.584 | 3.24E-02 |
| fibrinogen alpha chain | A_05_P332052 | 2.506 | 1.24E-02 |
| beta-enolase-like isoform 1 | A_05_P482637 | 2.500 | 4.06E-02 |
| fibrinogen gamma chain | A_05_P464657 | 2.463 | 1.82E-02 |
| pyruvate kinase | A_05_P253514 | 2.381 | 2.45E-02 |
| alpha-1-microglobulin bikunin precursor | A_05_P332632 | 2.137 | 3.93E-02 |
| fibrinogen gamma polypeptide | A_05_P450362 | 2.045 | 4.47E-02 |
| complement component c3 | A_05_P433922 | 2.024 | 2.44E-02 |
| pyruvate kinase | A_05_P390772 | 2.004 | 2.52E-02 |
| coagulation factor ii precursor | A_05_P364912 | 1.919 | 2.10E-02 |
| Ca2+-dependent complex c1r c1s subunit | A_05_P457602 | 1.919 | 3.93E-02 |
| beta-2-glycoprotein 1-like | A_05_P275734 | 1.890 | 3.57E-02 |
| DNA replication licensing factor mcm7 | A_05_P266444 | 1.838 | 1.63E-02 |
| complement component c9 | A_05_P485237 | 1.748 | 4.63E-02 |
| Telethonin | A_05_P263889 | 1.695 | 1.79E-02 |
| Telethonin | A_05_P413737 | 1.616 | 3.15E-02 |
| glutathione peroxidase | A_05_P365217 | 1.585 | 2.46E-02 |
| c1 inhibitor | A_05_P265959 | 1.548 | 4.31E-02 |
| antithrombin-iii precursor | A_05_P445252 | 1.527 | 4.36E-02 |
| fibronectin precursor | A_05_P263834 | 1.458 | 2.50E-02 |
| complement c3-like | A_05_P490817 | 1.439 | 4.24E-02 |
| antithrombin-iii precursor | A_05_P249134 | 1.346 | 2.91E-02 |

| **Supplementary Table 19b: Genes Up-regulated in White Muscle in Small Rainbow Trout compared to Large Rainbow Trout in GO ‘response to stress’ category** | | | |
| --- | --- | --- | --- |
| **Gene Name** | **Gene Number** | **Fold change^a^** | **p-value^b^** |
| ***Sept Fish*** |  |  |  |
| myosin heavy chain | A_05_P393052 | 2.682 | 1.37E-02 |
| tyrosine-protein phosphatase non-receptor type 12 | A_05_P478272 | 2.013 | 7.26E-03 |
| calcium calmodulin-dependent protein kinase (kinase) ii delta 2 | A_05_P304402 | 1.819 | 3.67E-02 |
| histone h2a-like | A_05_P474392 | 1.445 | 1.97E-02 |
| platelet glycoprotein 4 | A_05_P270029 | 1.442 | 2.62E-02 |
| proteasome subunit alpha type-6 | A_05_P436627 | 1.435 | 2.36E-02 |
| electron-transferring-flavoprotein dehydrogenase | A_05_P308382 | 1.433 | 4.30E-03 |
| proteasome subunit alpha type-5 | A_05_P409612 | 1.430 | 7.45E-03 |
| der1-like domain member 2 | A_05_P478862 | 1.420 | 4.00E-02 |
| aminoacyl trna synthase complex-interacting multifunctional protein 1 | A_05_P407602 | 1.410 | 3.00E-02 |
| dual specificity phosphatase 1 | A_05_P383357 | 1.406 | 2.28E-02 |
| smc3 protein | A_05_P385862 | 1.396 | 3.59E-03 |
| thioredoxin interacting protein | A_05_P395257 | 1.393 | 4.04E-02 |
| polyadenylate-binding protein 4 isoform 3 | A_05_P450567 | 1.380 | 3.48E-02 |
| arginine- mutated in early stage tumors | A_05_P367577 | 1.365 | 2.47E-02 |
| transcription factor | A_05_P308537 | 1.363 | 1.88E-02 |
| glutathione synthetase | A_05_P489127 | 1.349 | 4.67E-02 |
| coagulation factor x precursor | A_05_P457147 | 1.347 | 3.04E-02 |
| heat shock protein hsp 90-alpha | A_05_P479602 | 1.332 | 4.09E-02 |
| high mobility group protein b2 | A_05_P253324 | 1.325 | 1.27E-02 |
| ubiquinone biosynthesis protein coq7 homolog | A_05_P344462 | 1.323 | 2.01E-02 |
| 26s proteasome non-ATPase regulatory subunit 3 | A_05_P488577 | 1.321 | 4.31E-02 |
| ubiquitin c | A_05_P418397 | 1.320 | 3.25E-02 |
| cyclic amp-dependent transcription factor atf-4 | A_05_P253389 | 1.311 | 1.93E-02 |
| Calmodulin | A_05_P377098 | 1.301 | 3.56E-02 |
| apoptosis-enhancing nuclease | A_05_P417057 | 1.300 | 2.90E-02 |
| mapk mak mrk overlapping kinase | A_05_P416647 | 1.294 | 4.95E-02 |
| Prohibitin | A_05_P414182 | 1.293 | 1.67E-02 |
| monoglyceride lipase | A_05_P392532 | 1.286 | 3.42E-02 |
| proteasome ( macropain) 26s 6 | A_05_P406557 | 1.279 | 4.02E-02 |
| DNA-directed rna polymerase ii subunit rpb2 | A_05_P418202 | 1.270 | 4.73E-02 |
| selenoprotein s | A_05_P276549 | 1.270 | 4.49E-02 |
| fast myotomal muscle tropomyosin | A_05_P464747 | 1.263 | 3.19E-02 |
| 5-aminolevulinate mitochondrial | A_05_P467616 | 1.262 | 4.84E-02 |
| 26s protease regulatory subunit 8 | A_05_P252159 | 1.260 | 3.39E-02 |
| regulator of g-protein signaling 14 | A_05_P309402 | 1.252 | 2.48E-02 |
| capping protein (actin filament) muscle z- beta | A_05_P410762 | 1.251 | 4.63E-02 |
| DNA damage-binding protein 1 | A_05_P453112 | 1.247 | 4.30E-02 |

^a^ Fold change is the average difference in expression as measured by the microarray

^b^ Measures the significance of the difference in expression between the small and large fish.

Colours in Gene Number column:

Probe Ids with proportionally higher counts in large Sept. fish within the GO ‘response to stress’ category.

Probe Ids with proportionally lower counts in small Sept. Fish within the GO ‘response to stress’ category.
